# Efficient learning representation of noise-reduced foam effects with convolutional denoising networks

**DOI:** 10.1371/journal.pone.0275117

**Published:** 2022-10-10

**Authors:** Jong-Hyun Kim, YoungBin Kim

**Affiliations:** 1 School of Software Application, Kangnam University, Yongin, Gyeonggi, Republic of Korea; 2 Graduate School of Advanced Imaging Science, Multimedia & Film, Chung-Ang University, Seoul, Republic of Korea; Mae Fah Luang University, THAILAND

## Abstract

This study proposes a neural network framework for modeling the foam effects found in liquid simulation without noise. The position and advection of the foam particles are calculated using the existing screen projection method, and the noise problem that occurs in this process is prevented by using the neural network. A significant problem in the screen projection approach is the noise generated in the projection map during the projecting of momentum onto the discretized screen space. We efficiently solve this problem by utilizing a denoising neural network. Following the selection of the foam generation area using a projection map, the foam particles are generated through the inverse transformation of the 2D space into 3D space. This solves the problem of small-sized foam dissipation that occurs in conventional denoising networks. Furthermore, by integrating the proposed algorithm with the screen-space projection framework, it is able to maintain all the advantages of this approach. In conclusion, the denoising process and clean foam effects enable the proposed network to model the foam effects stably.

## Introduction

Physics-based fluid simulation has been used to realize various visual special effects to simulate water [1, 2], fire [3–5], smoke [6–8], fire-flake [9–11], foam [12, 13], bubble [14, 15], and mist (or spray) [16, 17]. When expressing water, the associated secondary effects such as foam, bubble, and splash are caused by oscillating movements, and various approaches have been proposed to efficiently model these characteristics [18, 19]. In general, splashes, foams, and bubbles are modeled by analyzing the flow of the underlying fluids, and movement is controlled by adopting a different advection method depending on the type of material. This approach, however, requires a large amount of computation to consider the movement of 3D fluid, and various screen-space projection approaches have been proposed to process them efficiently [20, 21]. Although these approaches use a depth map and a normal map to model the bubble effect in real time, they are inefficient in accurately modeling foam or splash in 3D space because they use only 2D space. Failure to model the advection of bubbles results in a sense of heterogeneity. Consequently, the screen projection approach has primarily been applied to real-time applications, such as games.

**Fig 1:**
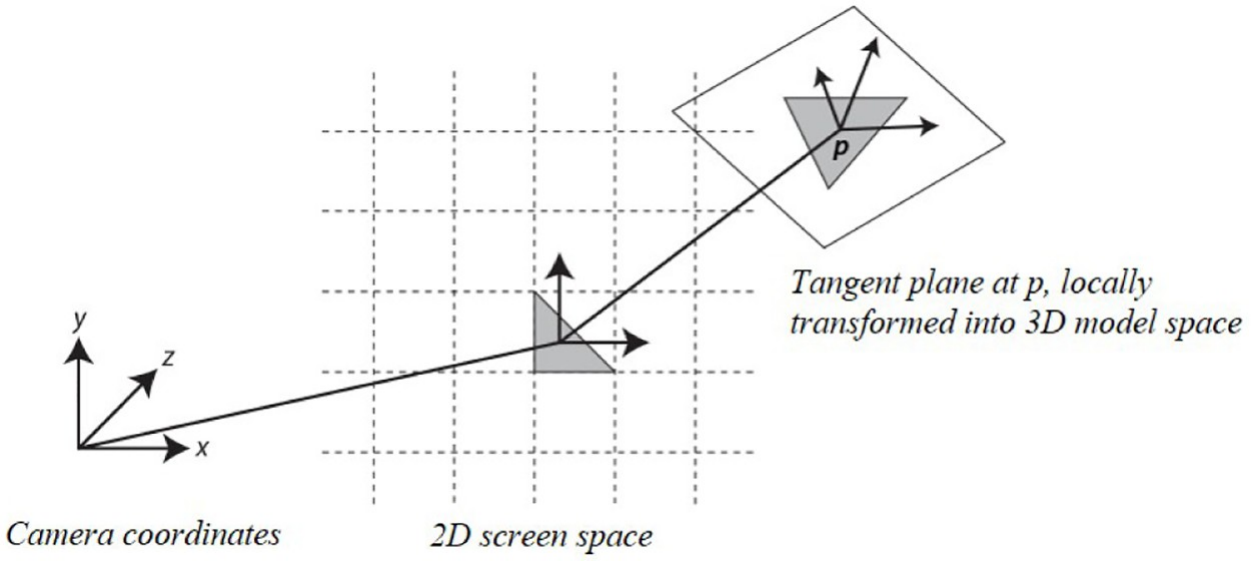
Illustration of the inverse transformation of 2D foam-sourcing triangle into 3D space [22, 23].

**Fig 2:**
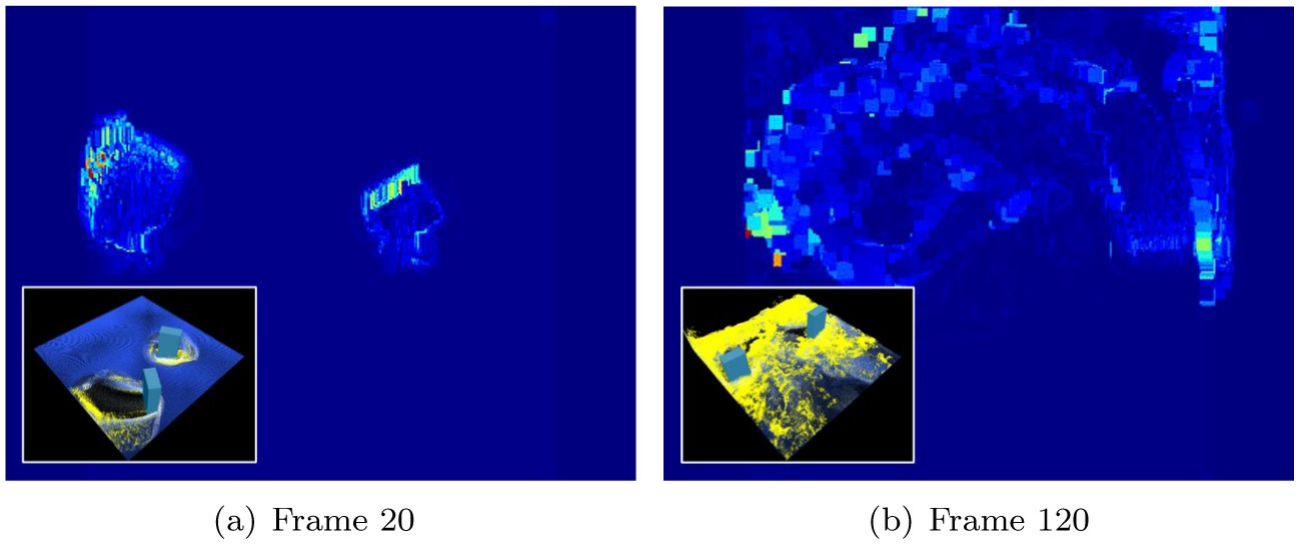
Aliasing problem that occurred while projecting 3D particles into 2D space (red: Faster acceleration). (a) Frame 20, (b) Frame 120.

**Fig 3:**
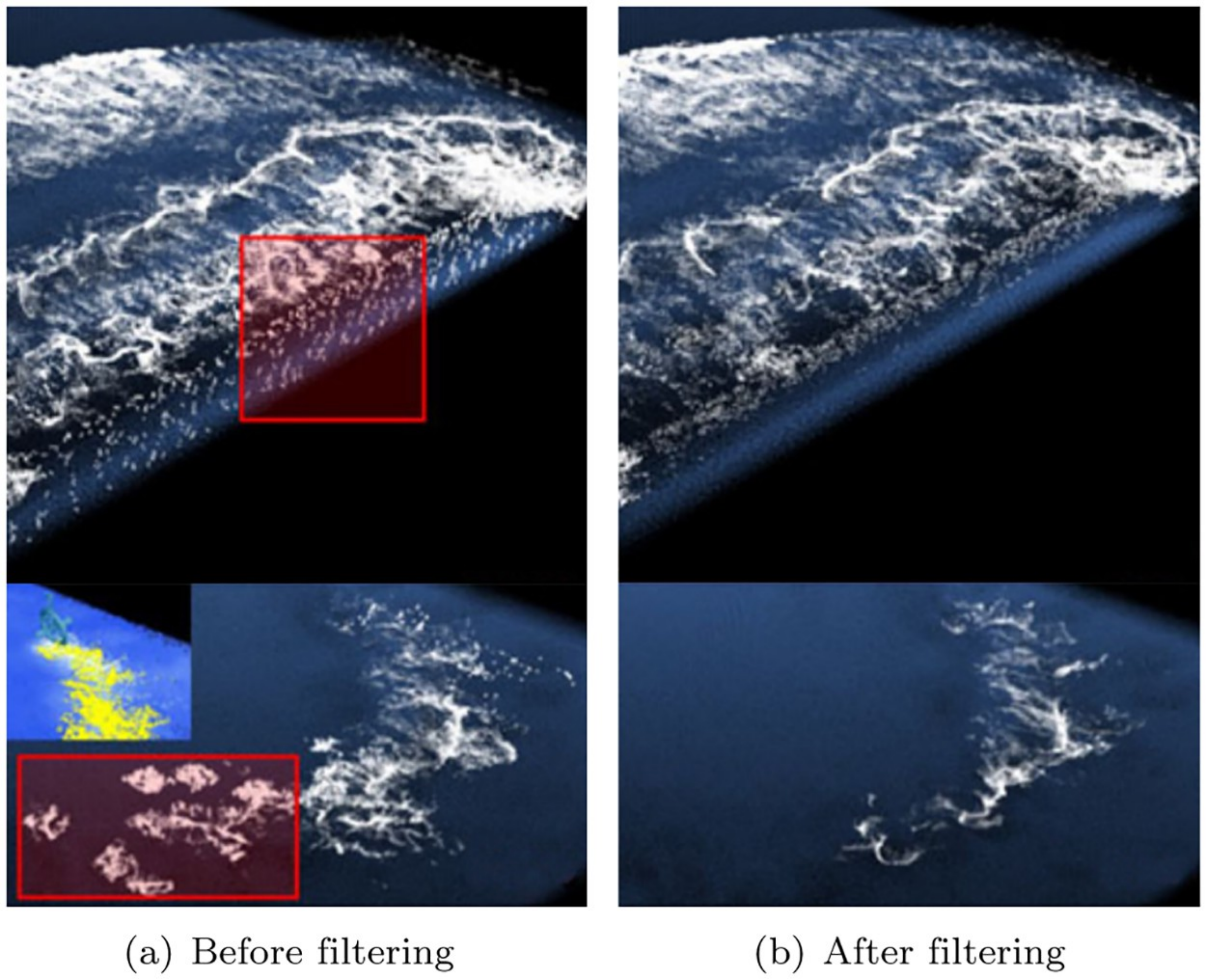
The filtering technique used in the previous methods [22, 23] (red box: Noise area). (a) Before filtering, (b) After filtering.

Kim et al. recently improved the efficiency and quality of foam visualization using both 2D and 3D space [22, 23]. As shown in Fig 1, by projecting the motion of the 3D fluid onto the 2D screen space, candidate groups that have a high probability of generating bubbles are selected. Subsequently, the 2D space is inverse-transformed to 3D space to generate bubbles. This alleviates the heterogeneity felt when only 2D space is used and the bubble movement is modeled realistically. The process of projecting particle motion into 2D space, however, causes an aliasing problem, which may affect the quality of the foam. Fig 2 shows the foam generated by rotating two boxes and a model of the resultant acceleration map. The map projected into 2D space, as shown in Fig 2, causes aliasing problems near the boundary as fast and slow particles are mixed. A separable binomial filter is used to solve this problem; however, it causes foam loss. The noise problem that occurs in the projection map also significantly affects the creation of foam (see Fig 3A). In particular, when fluid particles are sourced or particles are splattered in the air, such as when splashes collide with the water surface at high speed, noisy foam and flickering problems in continuous frames may occur. Although this problem may be solved by applying a separable binomial filter, significant foam loss can occur in the process (see Fig 3B).

This study proposes a framework that models foam without loss using a neural network-based projection map refinement technique. The implementation of the proposed technique requires solving the following sub-problems from the motion of the input fluid:

1. **Collection of Projection map data**. This is a process of collecting data for learning the projection map. In this study, after projecting the movement of particles into 2D space using a technique proposed by Kim et al. [22, 23], the projection map data after refinement are collected through an adaptive binomial filter.
2. **Neural network design for noise reduction**. The noise reduction network is modeled using a convolutional neural network (CNN) based on residual compensation.
3. **Integrated foam framework using neural network**. A new method for integrating the noise-removed projection map into the existing foam creation framework is proposed.

Solving the first problem allows us to collect the data needed to train the network. This data-construction step is an important process to refine the projection map without loss of foam. A solution for the second problem is essential to learn a noise-reduced weight from the data, that is, a noise-reduced projection map is obtained in the test stage using the weight. Solving the last problem allows us to express the foam generation by integrating the network process into the existing bubble generation algorithm efficiently.

In general, foam is a product of fluid motion calculated in 3D. Consequently, splashes, foam, and bubbles are called secondary effects. Notably, owing to the nature of 3D, the amount of computation required to calculate fluid motion is enormous, and the amount increases exponentially in the calculation of secondary effects. In this study, we avoid computational problems and efficiently generate foam through a foam generation approach that reduces the amount of computation by projecting fluid motion onto a screen. However, because the fluid motion is projected onto a 2D screen, not 3D space, it is sensitive to even small movements, and a noise pattern appears during the foam generation process. Owing to dimension reduction, the foam generation may flicker in the continuous time changes, or noisy results may be produced because of a small number of water particles with large momentum (see Fig 3). To solve the above problems, this study proposes a new denoising network architecture that generates foam efficiently without noise.

### Related work

This section briefly reviews the physics-based foam generation technique, the foam visualization technique using screen space, the simulation technique using AI, and the artificial neural network technique for noise removal, which are closely related to this study.

### Foam modeling with physically based approach

Physics-based simulations are continuously being studied to develop methods to model foam and motion. Takahashi et al. analyzed the motion of the underlying fluids based on curvature, and modeled the foam effect by applying the state change rule to the area with large motion [24]. However, this approach is unable to model various foam effects because it has a particle motion similar to a grain of sand as opposed to foam. Geiger et al. proposed a multilayering method to effectively handle the foam-generation process [25]. In this method, each layer that can express foam, splash, water drop, and mist is calculated; however, this method results in foam clumping. In addition, the method focuses solely on foam creation and rendering, which is not sufficient to realistically represent foam movement, thereby making it difficult to use in various scenes. In the method using the Eulerian model, secondary effects, such as splash are modeled using particle level-set or maker-particle methods. Kim et al. introduced a method to model splashes and water droplets without ignoring maker-particles falling from the liquid surface [19]. However, because most of them have a ballistic movement, there is a limit to expressing detailed foams. Losasso et al. developed a particle level-set technique to improve the aforementioned method to express splashes, and applied the smoothed particle hydrodynamics (SPH) technique to splash particles to improve ballistic movement [26]. This enabled the modeling of diffuse phenomena existing between air and splash particles, which was difficult to model using conventional ballistic motion. This method focused on the splash and the foams were expressed using textures.

Mihalef et al. solved the problem of dissolved gas based on SPH and realistically visualized carbonated drinks, which were difficult to express in the past [27]. Ihmsen et al. expressed phenomena such as splash, foam, and bubble using only the SPH framework [18]. However, because the quality depends on the particle number, achieving high quality results requires a large number of SPH particles, which increases the computation time. In addition, SPH kernel causes a problem of foam particles agglomeration. Wang et al. improved Ihmsen et al.’s method efficiently by proposing a hybrid technique that mixes SPH and Lattice Boltzmann techniques [28]. However, this method mostly models and expresses splashes and the visualization of other effects such as foams and bubbles is significantly limited or almost imperceptible.

Yue et al. modeled continuum foam using the material point method (MPM), by which shear-dependent flows of shaving cream or whipped cream can be generated. However, the foam targeted by their method differs from the sea foam targeted in this study as it generates dense foams composed of microscopic bubbles [29]. Similar to Yue et al., Ram et al. modeled foam effects based on MPM. However, they generated viscoelastic fluids of sponges rather than sea foam [30]. Although MPM is more accurate numerically than FLIP or SPH, it requires more computation. The previous studies applied it mainly to shear motion or viscoelastic fluids generated in large-scale foam that does not have many foam particles. The application of this method to generate sea foam, which has numerous foam particles, thus requires more computational resources and is not efficient. Furthermore, these methods are not applicable to sea foam that has characteristics different from those of shaving cream or cake.

### Foam modeling with screen space approach

Foam modeling using the screen space approach is commonly employed in real-time applications, such as games and virtual reality. To avoid the tessellation problem found in grid-based approaches, Van der Laan et al. proposed a new framework for rendering particles in screen space [20]. The noise that occurs during projection onto the 2D screen space was mitigated through the curvature flow filtering technique. Although in this method, the foam effect was modeled using a simple noise texture, it was difficult to expect a realistic foam effect. Bagar et al. processed different types of fluids with separate layers to create a more realistic and 3D foam effect [21]. Although the screen rendering technique has been modified in other various ways [31–33], it is not sufficient to visualize even the movement of foam because it uses only 2D space.

To solve this problem, Kim et al. utilized both 2D and 3D spaces to improve the efficiency and quality of foam [22, 23]. In this method, the location where foams are to be generated was quickly found using 2D space, and the foam particles were advected by inverse-transformation of this space into 3D space.

### Fluid modeling with artificial neural networks

Recently, artificial neural networks have been used to improve physics-based simulations. Specifically, in the field of fluid simulation, the Poisson equation, which must be solved when calculating high-resolution pressure, has been replaced with the super-resolution (SR) process to ensure efficiency maximization [34–36]. More recently, the use of deep learning to directly process high-resolution simulations has also been proposed; specifically, approaches to obtain simulation details using ConvNet or generative adversarial network (GAN) [37] have been proposed. Tompson et al. [38] and Xiao et al. [39] solved the Navier-Stokes equation efficiently by using a ConvNet-based approach. These studies performed SR using previously obtained low-resolution smoke simulation results as opposed to directly solving the simulation equation. Recently, Hong et al. proposed a method to efficiently perform SR with an octree-based adaptive structure [40].

ConvNet and GAN are deep learning methods that are widely used in SR studies. Dong et al. proposed a ConvNet-based solution for processing a single image SR [41], and Ledig et al. [42] and Chu et al. [43] proposed a GAN-based method for image SR. Chu and Thüerey used ConvNet models to synthesize high-resolution smoke simulations [35] and Werhah et al. used GAN to simulate SR. Bai et al. proposed an SR that generates high-resolution smoke details using deep learning-based dynamic features [44]. Notwithstanding these various approaches, there have been no attempts to apply deep learning to splash or foam. This study proposes a neural network-based method that alleviates noisy projection maps (a problem that frequently arises in conventional frameworks) to generate foam based on screens. Our method was found to cleanly model the details of the high-resolution foam effect without losing foam.

### Artificial neural network for noise removal

This section reviews artificial neural network-based denoising techniques used for images and videos. Deep learning methods use a large set of image pairs instead of presetting the image data to directly learn noise removal from images containing noise through a deep neural network. Jain and Seung proposed a noise removal method using a five-layers ConvNet [45], and some studies extended this method to create an autoencoder-based method [46, 47]. Burger et al. presented a method that has performance similar to block-matching and 3D filtering (BM3D) [48] using multi-layer perceptron [49]. Zhang et al. proposed denoising ConvNet (DnCNN) and introduced a method to solve the Gaussian denoising problem [50]. Mao et al. proposed a very deep convolutional encoder-decoder networks technique using a symmetric skip connection [51]. Tai et al. proposed very deep persistent memory networks (MemNets) to continuously learn memory through an adaptive learning process that can be used for image restoration [52]. More recently, NLRN [53], N3Net [54], and UDNet [55] have included non-local properties of image in DNN to facilitate denoising tasks. In addition, to increase flexibility for spatial variant noise, a technique called FFDNet, which pre-evaluates the noise level and uses it as an input to the network along with images containing noise, has been introduced [56]. Guo et al. [57] and Brooks et al. [58] attempted to simulate the denoising process of camera images. However, most denoising methods are created based on image data, and application of these techniques to 3D simulation can cause detail loss. This study shows the foam loss caused by using a denoising network that uses image data as input, and demonstrates through experiments how accurately the technique proposed in this study models foam particles.

## Proposed framework

This study computes the underlying fluid simulation using the fluid-implicit particle (FLIP) method, and a hybrid method that uses the grid-particle method is employed to advect the fluid particles. A previously proposed screen-space projection technique is used for foam generation [22, 23]. The methods proposed in this study are described in detail following the review of the basic foam generation methods in Section “Foam Representation with Screen-Space Approach”. The algorithm proposed in this study is executed in the following order. (A list of symbols is available in Table 1).

- Preprocessing
  1. 3D fluid particles are projected onto screen space and data are built to be used as a network through an adaptive binomial filter.
  2. Projected map data are learned through ConvNet-based denoising network. This process is repeated during the projection map refinement (used as a test in the Online-2 process).
- Online
  1. Fluid particles advected by FLIP are projected onto screen space through a projection matrix. In this process, acceleration and depth, which are the physical quantities of the particles, are projected.
  2. Two projection maps: the acceleration map and the depth map are refined through the denoising network.
  3. Using a refined acceleration map, the space in 2D screen where foams are likely to be generated are quickly located.
  4. A 2D position is converted to a 3D position by performing inverse-transformation on candidate positions found in screen space. Subsequently, foam particles are created and advected in 3D space.

**Table 1:** Simulation parameters.

Name	Description	Value
**Z**	Depth map	–
Z*	Refined depth map	–
**D**	Acceleration map	–
D*	Refined acceleration map	–
**P**	Projection matrix	–
**Q**	Inverse projection matrix	–
**C**	Candidate region in 2D	–
C*	Refined candidate region in 2D	–
**F**	Curvature map with **D**	–
F*	Curvature map with D*	–
*r*	Radius of water particle	–
*x*_*p*_, *y*_*p*_, *z*_*p*_	Projected coordinate	–
*r*_*x*_, *r*_*y*_, *r*_*z*_	Projected radius	–
*d*_*p*_	Projected acceleration	–
Δ t	Time-step	0.006
*α*	Weighting for binomial filter	12.0
*β*	Curvature threshold	0.1
*h*	Projective spacing	2.0
*N*_*x*_ × *N*_*y*_	Projective space res.	400×300
*n*_filter_	Size of binomial filter	3

**Fig 4:**
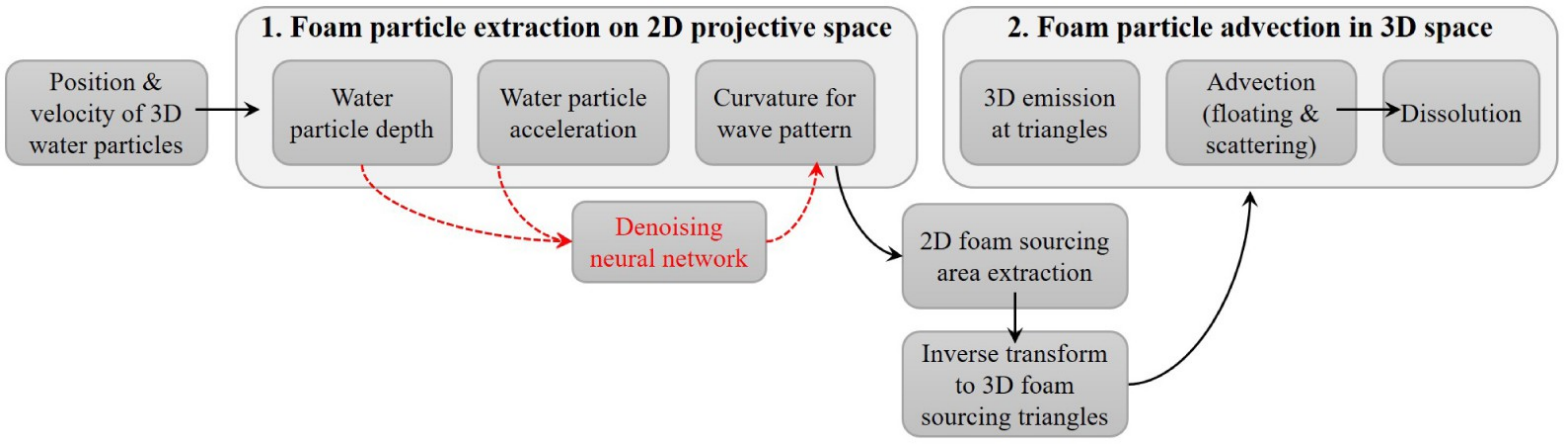
Algorithm overview.

Fig 4 shows an overview of the algorithm proposed in this study. After the position and velocity of water particles in 3D space are determined using the Navier-Stokes equation, the physical quantities of the particles are projected onto 2D space (*Step* 1 in Fig 4). Because a noise problem occurs in this process, a denoising stage is added to solve it. The main purpose of our study is to use a neural network to solve the noisy artifacts that appear during the process of water particles projection. After the waver pattern is extracted through physical quantities subjected to representation learning by the neural network, it is inversely transformed to generate foam particles in 3D space and advected (*Step* 2 in Fig 4). For more detailed explanations, please see [22, 23].

### Foam representation with screen-space approach

#### Projection map generation from fluid particles

In this section, the screen space-based foam method used in this study is briefly described [22, 23]. First, the acceleration and depth maps of the fluid particles existing in 3D space are calculated through screen projection. *W* and *H* represent the horizontal and vertical pixel resolution in screen space, respectively. *N*_*x*_ × *N*_*y*_ is the resolution of the regular grid divided by the projection interval *h*, and *r* is the radius of the fluid particle. *r*, which is also a user-adjustable value, is the range used to obtain a smooth projection map during projection. The depth value *z*_*ij*_ and the acceleration value *d*_*ij*_ of the fluid particle are converted into projection coordinates, and each map is composed as follows: depth map $Z\in R^{N_{x}\times N_{y}}$, acceleration map $D\in R^{N_{x}\times N_{y}}$. The particle’s acceleration *d*_*ij*_ is simply calculated from the difference in velocity between frames: |**v**_*t*+Δ*t*_ − **v**_*t*_|.

Particles **x** in 3D space [*x*, *y*, *z*, 1]^*T*^ are transformed into 2D projection space using the projection matrix **P** (see Eq 1).

$$\begin{array}{c} \left[\begin{array}{c} x^{\prime }\\ y^{\prime }\\ z^{\prime }\\ w \end{array}]=P[\begin{array}{c} x\\ y\\ z\\ 1 \end{array}\right] \end{array}$$ (1)

To avoid distortion of the *z* values during projection, perspective division is applied to *x*, *y*, except for the *z* values. Using this method, the projected coordinates (*x*_*p*_, *y*_*p*_), *z*_*p*_ and the projected acceleration *d*_*p*_ of the 3D fluid particle are calculated (see Eq 2).

$$\begin{array}{c} \underset{projected\;coordinates\;and\;depth}{\underset{︸}{\left[\begin{array}{c} x_{p}\\ y_{p}\\ z_{p} \end{array}]=[\begin{array}{c} W\cdot (\frac{1}{2}+\frac{1}{2}x^{\prime }/w)\\ H\cdot (\frac{1}{2}+\frac{1}{2}y^{\prime }/w)\\ z^{\prime } \end{array}\right]}},\underset{acceleration}{\underset{︸}{\left[\begin{array}{c} x_{d}\\ y_{d}\\ d_{p} \end{array}]=[\begin{array}{c} x_{p}\\ y_{p}\\ d \end{array}\right]}}, \end{array}$$ (2)

(*x*_*d*_, *y*_*d*_) is the index of the array in which the acceleration *d*_*p*_ in screen space is stored.

The particle radius *r* in 3D space is projected using the following method (see Eq 3):

$$\begin{array}{c} {}[\begin{array}{c} r_{x}\\ r_{y}\\ r_{z} \end{array}]=[\begin{array}{c} rW\sqrt{p_{1,1}^{2}+p_{1,2}^{2}+p_{1,3}^{2}}/w\\ rH\sqrt{p_{2,1}^{2}+p_{2,2}^{2}+p_{2,3}^{2}}/w\\ r\sqrt{p_{3,1}^{2}+p_{3,2}^{2}+p_{3,3}^{2}} \end{array}], \end{array}$$ (3)

*p*_*i*,*j*_ is an element of the projection matrix **P**. To obtain isotropically projected radius values, *r*_*x*_ and *r*_*y*_ are set to *r*_*p*_.

Because numerous fluid particles may be projected onto a node in screen space, the depth and acceleration values are updated as follows (see Eq 4):

$$\begin{array}{c} z_{ij}\leftarrow \text{min}\left(z_{ij},z_{p}-r_{z}h_{ij}\right),\;d_{ij}\leftarrow \text{argmin}\left(z_{ij}\right) \end{array}$$ (4)

where

$$\begin{array}{c} h_{ij}=\sqrt{1-\frac{{\left(ih-x_{p}\right)}^{2}+{\left(jh-y_{p}\right)}^{2}}{r_{p}^{2}}} \end{array}$$ (5)

${\left(ih-x_{p}\right)}^{2}+{\left(jh-y_{p}\right)}^{2}\leq r_{p}^{2}$ is a term used to determine whether the projected coordinates are affected by each node in the projection space. If *z*_*p*_ − *r*_*z*_*h*_*ij*_, which is the depth value of the projected coordinates, is less than *z*_*ij*_, *z*_*ij*_, and *d*_*ij*_ are updated using Eq 4. Fig 2 shows the acceleration map in which 3D particles are projected onto screen space. However, as previously mentioned, an aliasing problem can occur. Section “Preprocessing: Collection of Projection Map Data”∼“Refinement of Projection Map with Denoising Neural Networks” describes an efficient means of handling this problem, and transfers the proposed technique. A new foam particle generation framework integrated with the technique is described in Section “Integration with Existing Solver”.

### Preprocessing: Collection of projection map data

**Fig 5:**
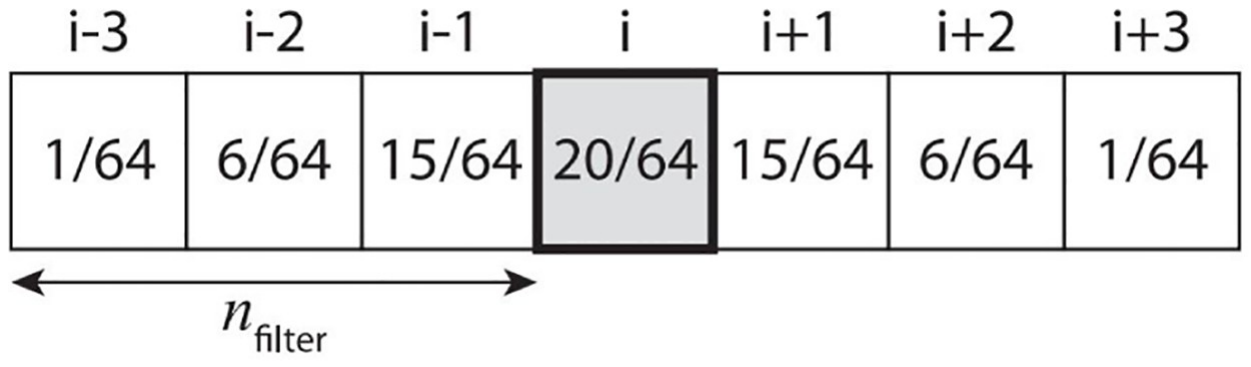
Separable binomial filter with size *n*_filter_ = 3. The acceleration value (*i*, *j*) shown in the center is the weighted sum of the neighboring values.

The data required for network learning in this study were acquired through physics-based simulation. Among the various approaches available to model the bubble effect, the method proposed by Kim et al. was applied in this study because it uses 2D space together with 3D space. Learning was performed using an artificial neural network. [22, 23]. To alleviate aliasing, we refined the data using an adaptive binomial filter (see Fig 5).

This filter aims to alleviate the aliasing problem of the projection map through the smoothing process. The smoothing parameter *n*_filter_ is user-adjustable and applied to all cases where *d*_*ij*_ ≠ ∞. In this study, the filter size is controlled according to the size of *d*_*ij*_ to minimize foam loss due to oversmoothing (see Eq 6).

$$\begin{array}{c} n_{\text{filter}}^{*}=\{\begin{array}{cc} |d_{ij}|\alpha & \text{,}\;\text{if}\;|d_{ij}|>\eta \\ 3 & \text{,}\;\text{else}\; \end{array} \end{array}$$ (6)

*α*, a user-adjustable variable, is set to 12 in this study.

**Fig 6:**
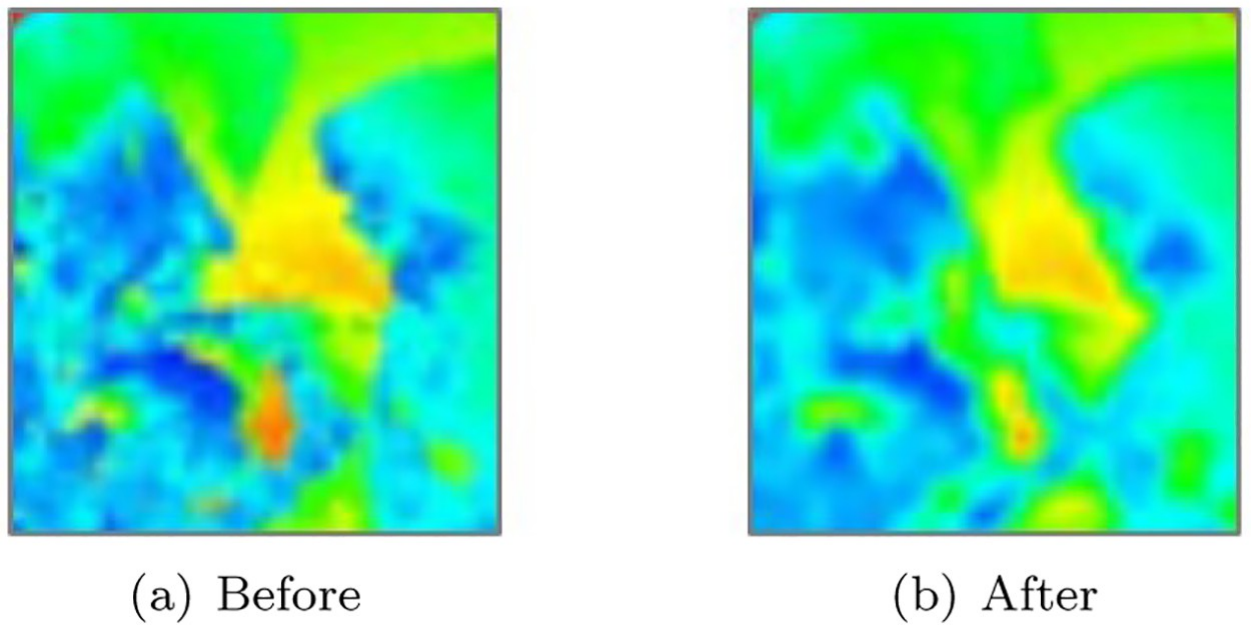
Comparison of aliasing artifacts. The pixels are colored according to the magnitude of *d*_*ij*_ from red (high) to the blue (low). (a) Before, (b) After.

Fig 6 shows the acceleration map before and after using the adaptive binomial filter. The acceleration map showing the rough shape is smoothed with the application of the filter (see Fig 6A), while maintaining the characteristic shape (see Fig 6B). In this study, pairs of projection maps were constructed for various foam scenes in the following process.

**Fig 7:**
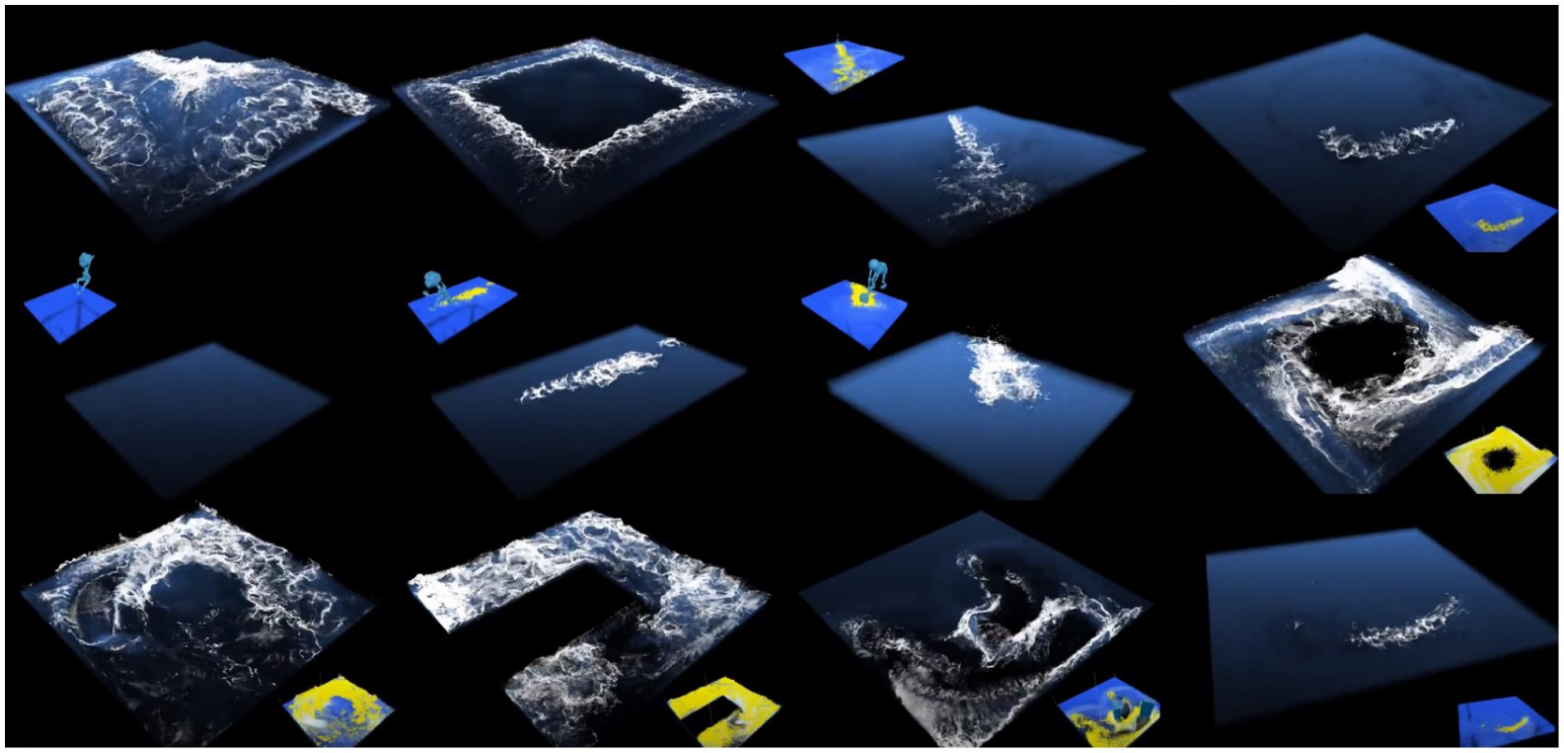
Examples by simulation.

Fig 7 shows the scenes used to construct the data. The data were constructed using projection maps (acceleration maps and depth maps) calculated for various scenes, which were created by collisions with solids and foam simulations by fluids. In the data employed for this study, 20,000 unfiltered projection maps existed. However, 5,000 projection maps, consisting of acceleration and depth maps, were used.

### Refinement of projection map with denoising neural networks

After obtaining the foam data {*F*^0^, *F*^1^, …} using physics-based simulation, a projection map onto which an adaptive binomial filter is applied using the method described in the previous section$\left\{\delta_{f_{w}}^{0},\delta_{f_{w}}^{1},...\right\}$ and the map onto which it is unapplied $\left\{\delta_{f_{wo}}^{0},\delta_{f_{wo}}^{1},...\right\}$ are generated. *δ* represents **D** and **Z** describes the above. The projection maps are partitioned into patches before being used in the training network. Our goal in using the training data is to find a mapping function *f*(**x**) that minimizes the loss between the predicted value *ν*_*s*_ and the ground truth (GT) $\delta_{f_{w}}$. The loss function for performing this process is the mean squared error (MSE) between the predicted projection map and the GT projection map. Our goal is to minimize *ν*_*s*_ = *f*(**x**).

Because the application of many weighting layers requires a large memory in the super-resolution CNN) technique (SRCNN), there is a limit to constructing a deep network [41]. To avoid this problem, this study trains and tests foam data through residual learning. The residual map of the input/output projection map is calculated as follows: $r=\delta_{f_{w}}-\delta_{f_{wo}}$. Although the loss function in the SRCNN technique is $\frac{1}{2}\left|\right|\delta_{f_{w}}-f\left(x\right){\left|\right|}^{2}$, this study computes the final loss function *L* as follows because we aim to predict the residual map (see Eq 7).

$$\begin{array}{c} L\left(r,x\right)=\frac{1}{2}\|r-{x\|}^{2} \end{array}$$ (7)

**r** is the residual and *x* is the value of *f*(**x**). In the network process, the loss layer is calculated using three factors: residual estimation, $\delta_{f_{wo}}$, and $\delta_{f_{w}}$. Loss is calculated as the Euclidean distance between the map reconstructed through the network and $\delta_{f_{w}}$, where the reconstructed map is the sum of the input and output maps of the network.

**Fig 8:**
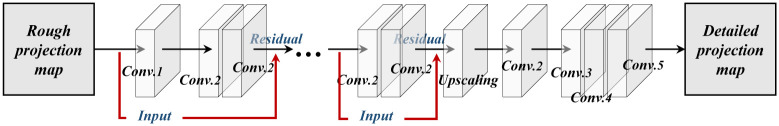
Feature extraction via denoising convolutional neural network (red arrow: Residual process).

This network is modeled based on ConvNet and has the following configuration (see Fig 8): When the feature map that has undergone the first ConvNet operation is re-added to the result value obtained through the two convolutions, residual compensation method is used. In this process, the error lost through the convolution operation is mitigated through residual compensation. In this study, this process is repeated ten times and a total of 20 convolution operations are performed because two convolutions are performed per cycle. The value that undergoes the first convolution is initially added. Subsequently, the previous result is repeatedly added. The size is then doubled through upscaling, and four convolution operations are completed before completion. The projection maps D* and Z* obtained in this manner are used to determine the generation of foam particles, which is explained in detail in the next section.

### Integration with existing solver

#### Generation and advection of foam particles

Using the refined projection map, a 2D candidate area with a potential for foam generation is extracted (see Eq 8).

$$\begin{array}{c} C=\{\left(i,j,k\right)|d>\gamma ,z\in Z^{*},d\in D^{*},\left(i,j\right)\in R^{N_{x}\times N_{y}}\;\}, \end{array}$$ (8)

*γ* is the threshold used to find the fast flow region, which is set to 0.0001 in this study. Z* and D* are depth maps and acceleration maps refined through the network. Reducing the value of *γ* makes it possible to create foam even in areas with slow flow, and allows the users to easily control the number of foams. The Marching squares algorithm is used to compose the extracted 2D foam area into a triangle, transforming the shape of the candidate group of foam particles into 2D triangles. These triangles are then transformed into 3D model space through the inverse transformation of Eqs 1 and 2. The coordinates of the transformed triangle are calculated as follows (see Eq 9).

$$\begin{array}{c} {}[\begin{array}{c} x\\ y\\ z\\ 1 \end{array}]=Q[\begin{array}{c} (-1+2x_{p}/W)w\\ (-1+2y_{p}/H)w\\ z_{p}\\ w \end{array}], \end{array}$$ (9)

where

$$\begin{array}{c} w=\frac{1-q_{4,3}z_{p}}{q_{4,1}\left(-1+2x_{p}/W\right)+q_{4,2}\left(-1+2y_{p}/H\right)+q_{4,4}}, \end{array}$$ (10)

*q*_*i*,*j*_ is an element of the inverse projection matrix **Q** and, as previously mentioned, perspective division is not applied to *z*_*p*_. The number of foam particles is determined using the Weber number. It is recommended to read Kim et al. for more detail information on this method [22, 23].

Next, the wave pattern of the foam is calculated using the refined projection map. Utilizing the method proposed by Van der Lann et al., the flow-based curvature is calculated at all values of the projected space [20] (see Eq 11).

$$\begin{array}{c} \frac{\partial z}{\partial t}=H, \end{array}$$ (11)

*t* is the time-step and *H* is the mean curvature of the depth map calculated in the 2D projection space. The depth map used in this process is Z*. The method used by Van der Lann et al. contains a noise pattern [20] mitigation, which requires iterative curvature calculation. Because the change pattern of the original curvature is lost in this approach, the wave pattern of the foam is calculated by modifying Eq 8 as follows (see Eq 12):

$$\begin{array}{c} C^{*}=\{\left(i,j,k\right)|d>\gamma \;\cap \;f>\beta ,f\in F^{*},\left(i,j\right)\in R^{N_{x}\times N_{y}}\;\}, \end{array}$$ (12)

F* is the curvature of the depth map, which is Z* refined through the network in this study. In addition, *β* is a threshold value for finding a high curvature region, which is set to 0.1 in this study. Advection for foam particles utilizes the lattice velocity field and fluid particles calculated through FLIP. This method uses the previous approach. For a detailed explanation, please see the previous study [22, 23].

## Implementation

**Details of simulation and rendering**. The proposed framework was implemented on a computer with an Intel i7–7700k 4.20 GHz CPU, 32 GB RAM, and an NVIDIA GeForce GTX 1080 Ti graphics card. A FLIP-based fluid solution was used as the underlying fluid simulation [59], and a GPU-based preconditioned conjugate gradient was used as a numerical solution to calculate the pressure [60]. For the FLIP grid, all momentum was stored using the Staggered Marker-and-Cell method [61], and the boundary particle technique proposed by Akinci et al. was used for collision handling between fluids and solids [62].

**Algorithm 1**: Pseudocode of our algorithm as an extension to the previous foam simulator [22, 23]

// Base water solver

1. Advect 3D water particles using FLIP

2. Compute the interaction of water & solids

// Our foam solver

3. Project 3D water particles on screen space

4. Denoise projection maps with neural network

5. Extract wave patterns by curvature

6. Find 2D foam-sourcing areas

7. Find 3D foam-sourcing triangles

8. Emit foam particles

9. Advect foam particles

10. Compute the interaction of foam & solids

11. Eliminate expired foam particles

For the final product, the particles were rendered by ray tracing without reconstructing the surface of the fluid. The colors of the fluid particle and foam particle were set to (0.8, 0.5, 0.3) and (1.0, 1.0, 1.0), respectively. The alpha value was set to 0.07. Each particle was projected onto the image space to be rendered, and 3×3 pixels were updated at the projected position as follows (see Eq 13).

$$\begin{array}{c} p_{i,j}=\{\begin{array}{c} p_{i,j}^{r}\leftarrow c_{a}c_{r}+\left(1-c_{a}\right)p_{i,j}^{r}\\ p_{i,j}^{g}\leftarrow c_{a}c_{g}+\left(1-c_{a}\right)p_{i,j}^{g}\\ p_{i,j}^{b}\leftarrow c_{a}c_{b}+\left(1-c_{a}\right)p_{i,j}^{b} \end{array}, \end{array}$$ (13)

($p_{i,j}^{r},p_{i,j}^{g},p_{i,j}^{b}$) represents the color of the pixel, and (*c*_*r*_, *c*_*g*_, *c*_*b*_) represents the color of the projected particle in image space. *c*_*a*_ is the alpha value. The pseudocode of the foam simulation algorithm is as follows (see Algorithm 1).

**Details of the denoising convolutional neural network architecture**. This study used a CNN architecture to design the denoising network. This section presents the details of the denoising convolutional neural network architecture. The denoising convolutional neural network comprises two main stages: feature extraction and feature reconstruction.

Assume that the input **x** is a three-channel image with a resolution of 128×128. The first ConvNet1 consists of two types of layers. First, we use 64 5×5 filters in a convolution operation with the ReLU activation function for better capturing of receptive fields. The pooling layer then operates on each depth slice of the input independently and resizes it spatially using the MAX operation. The pooling layer, which uses a filter size of 2×2 and a stride of 2, downsamples every depth slice in the input by 2 in both width and height. The output feature map is 64×64×64.

**Fig 9:**
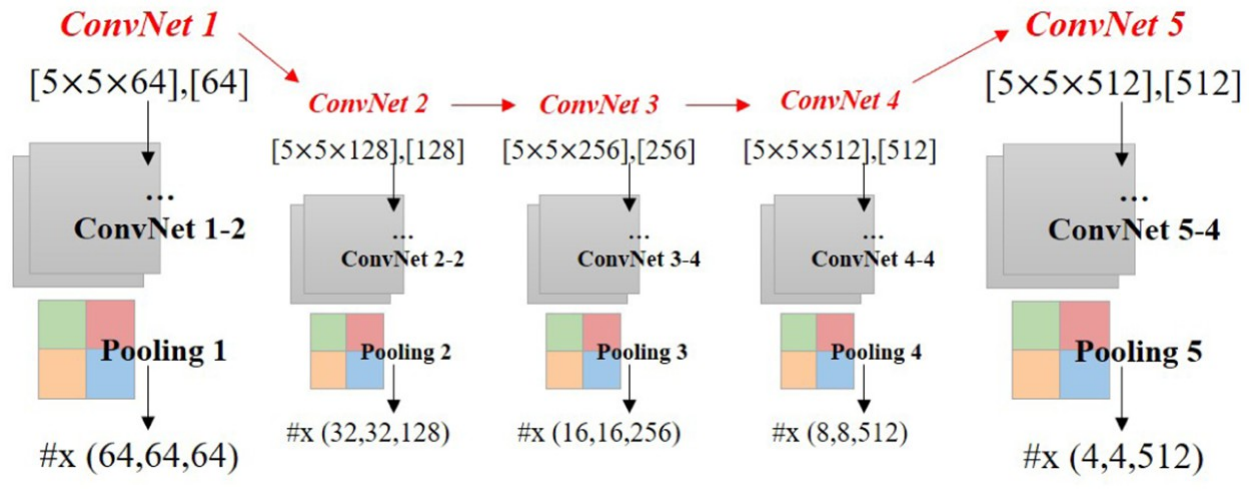
Denoising convolutional neural network architecture (input: x(128, 128, 3), [weight], [bias], #x(width, height, depth), final output: x(4, 4, 512)).

The output feature map of ConvNet1 is used as the input of ConvNet2, and the overall denoising convolutional neural network flow is as follows (see Fig 9). The ConvNet 1–2 in this figure denotes that two ConvNet layers are used in the ConvNet 1 process, and similarly, ConvNet 5–4 denotes that four ConvNet layers are used in the ConvNet 5 process.

**Fig 10:**
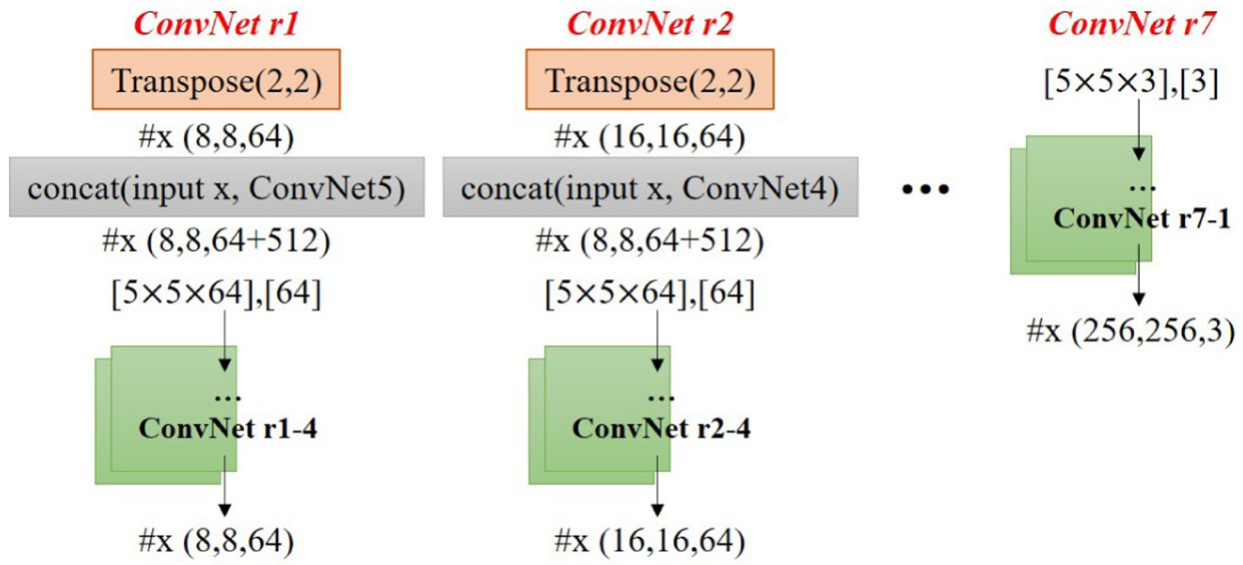
Feature reconstruction (input: x(4, 4, 512), output: x(256, 256, 3)).

**Fig 11:**
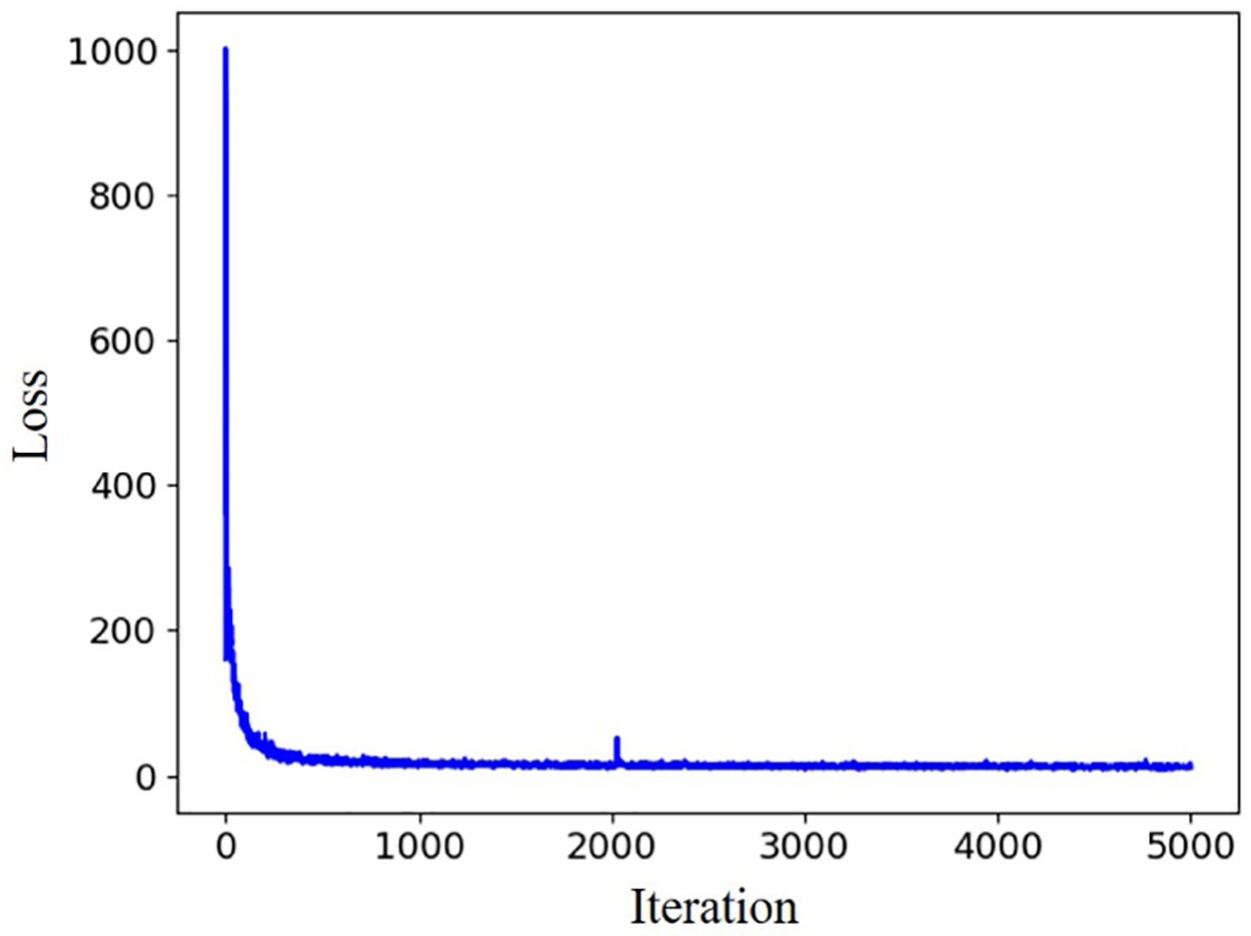
Training loss.

The reconstruction step is a process of restoring the result through transposed convolution of the feature map of the network (see Fig 10). The details of ConvNet r1 to ConvNet r7 are listed in Table 2. The input of the neural network is the size of $\frac{1}{2}$ of the original map, and when it is subsequently added to the residual map, the size is similar to that of the reconstruction output. This network was implemented in TensorFlow [63]. The adaptive moment estimation (Adam) was used as the optimizer. The curve of the MSE loss for 5,000 iterations is shown in Fig 11.

**Table 2:** Details of the denoising convolutional neural network architecture.

	ConvNet r1	ConvNet r2	ConvNet r3	ConvNet r4	ConvNet r5	ConvNet r6	ConvNet r7
Transpose(…)	(2,2)	(2,2)	(2,2)	(2,2)	(2,2)	(2,2)	–
#x(w,h,d)	(8,8,64)	(16,16,64)	(32,32,64)	(64,64,64)	(128,128,64)	(256,256,64)	–
concat(…)	(input x, ConvNet5)	(input x, ConvNet4)	(input x, ConvNet3)	(input x, ConvNet2)	(input x, ConvNet1)	–	–
#x(w,h,d)	(8,8,64+512)	–	–	–	–	–	–
[weight],[bias]	5×5×64,64	5×5×64,64	5×5×64,64	5×5×64,64	5×5×64,64	5×5×64,64	5×5×3,3
Num. ConvNet	ConvNet r1–4	ConvNet r2–4	ConvNet r3–4	ConvNet r4–2	ConvNet r5–2	ConvNet r6–2	ConvNet r7–1
#x(w,h,d)	(8,8,64)	(16,16,64)	(32,32,64)	(64,64,64)	(128,128,64)	(256,256,64)	(256,256,3)

In this study, the learning rate was set differently for each epoch using a learning rate schedular (LRS), specifically, exponential LRS [64], in which the rate of current step is taken exponentially: the exponent is set to lower [higher] than 1 when the length of the current step is shorter [longer] than the predetermined stage-length. In the data-pretreatment process, data were obtained without additive data augmentation through the separable binomial filter method explained above. The amount of data obtained in the pretreatment process is sufficient to produce the results of this study. Furthermore, more datasets, if needed, can be obtained easily only by adjusting the value of *n*_*f*_*ilter*. The values of beta1 and beta2 used in the Adam optimizer were set to 0.9 and 0.999, respectively.

The proposed technique did not cause overfitting or underfitting problems. Therefore, we believe that the amount of training data was sufficient to prevent overfitting. Furthermore, we believe that if it does occur, it can be solved by methods such as model capacity reduction, dropout techniques, L1 or L2 regularization, or data augmentation. The occurrence of underfitting was not sensitive, as shown by the training loss.

## Results and discussion

For a comprehensive analysis of the technique proposed in this study, the differences with the image-based denoising technique in terms of quality and continuity of the generated bubbles are analyzed.

**Fig 12:**
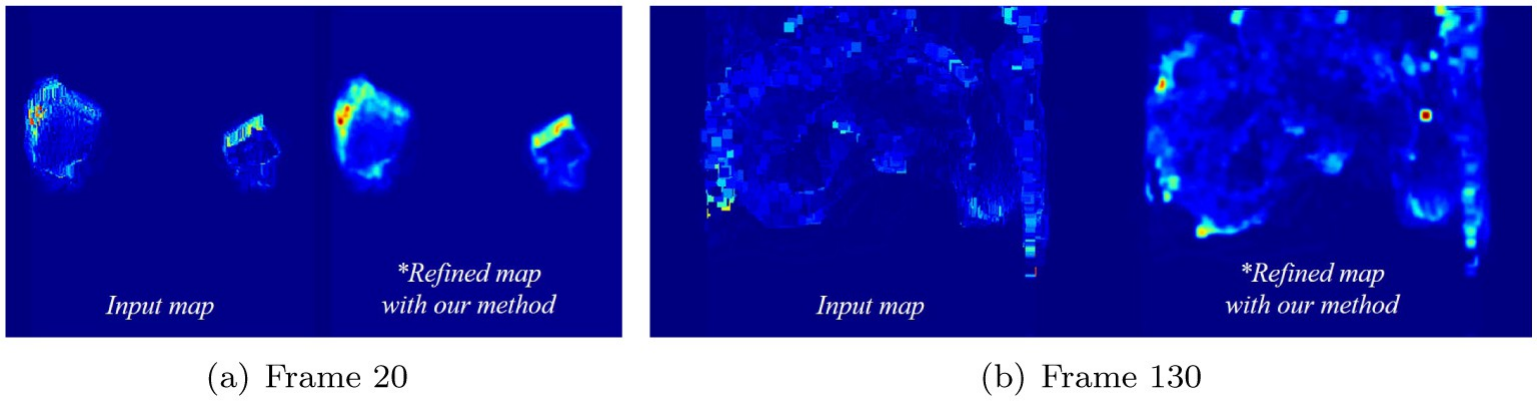
Refinement of acceleration map with our method (red: Fast flow zone). (a) Frame 20, (b) Frame 130.

To provide support for the quality of the proposed method, a scene in which two boxes stir a liquid is produced as a simple test scenario. To model the fluid in this scene, the time-step is set to 0.006 and approximately one million fluid particles are used. Fig 12 shows the refined acceleration map. The projection map used as input has aliasing and coarse noise in the result projected onto the screen. The refinement of the projection map through the proposed network not only alleviates the aliasing problem, but also maintains the shape of the original acceleration map.

**Fig 13:**
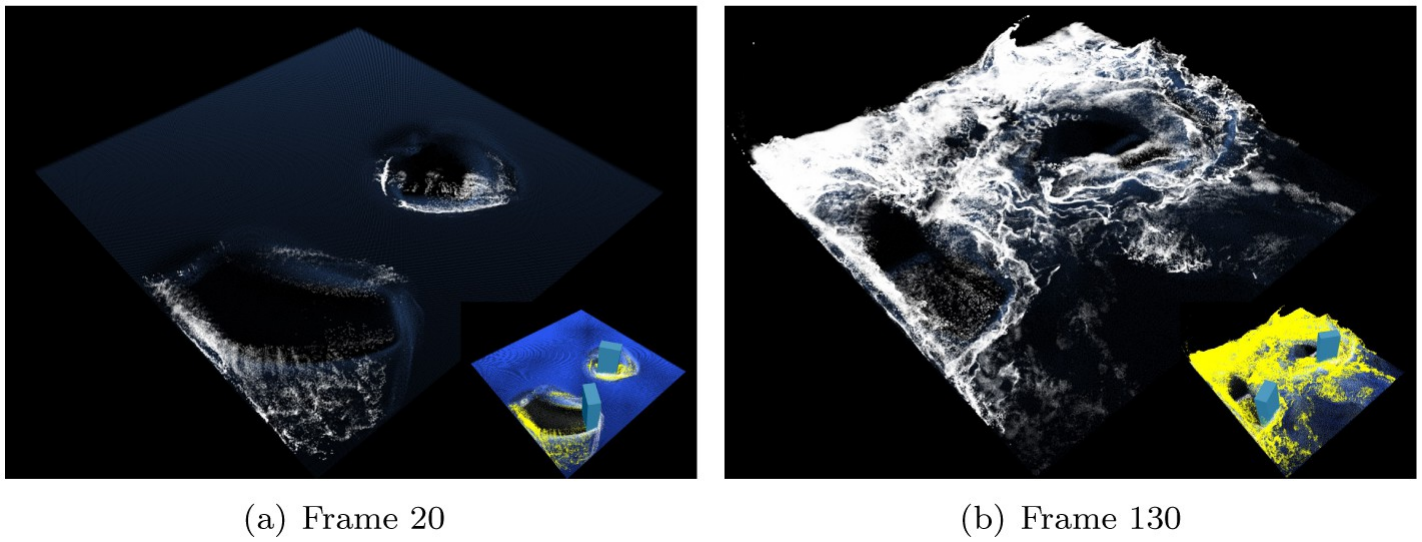
Foam effects with our method in two rotating boxes (inset image: Simulation view). (a) Frame 20, (b) Frame 130.

Fig 13 shows the foam effect created using the refined projection map. The refinement process produces stable foaming results without noise or foam loss. In particular, Fig 13B shows that the misty surface foam effects are accurately visualized. In the production of this scene, the foam advection technique suggested by Kim et al. is used [22], and the refinement technique suggested in this study realistically expresses thin and misty surface foam without loss.

**Fig 14:**
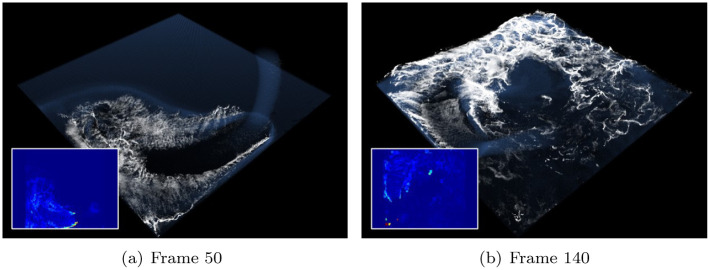
Foam effects with our method in a rotating-emitter (inset image: Acceleration map). (a) Frame 50, (b) Frame 140.

Fig 14 shows the scene where the emitter in the air is rotating and sourcing water particles, where foam effects are implemented. In addition to the foam generated by the water-solid interaction shown earlier, the foam effect is also excellently produced in the emitter scene based on the refined projection map.

**Fig 15:**
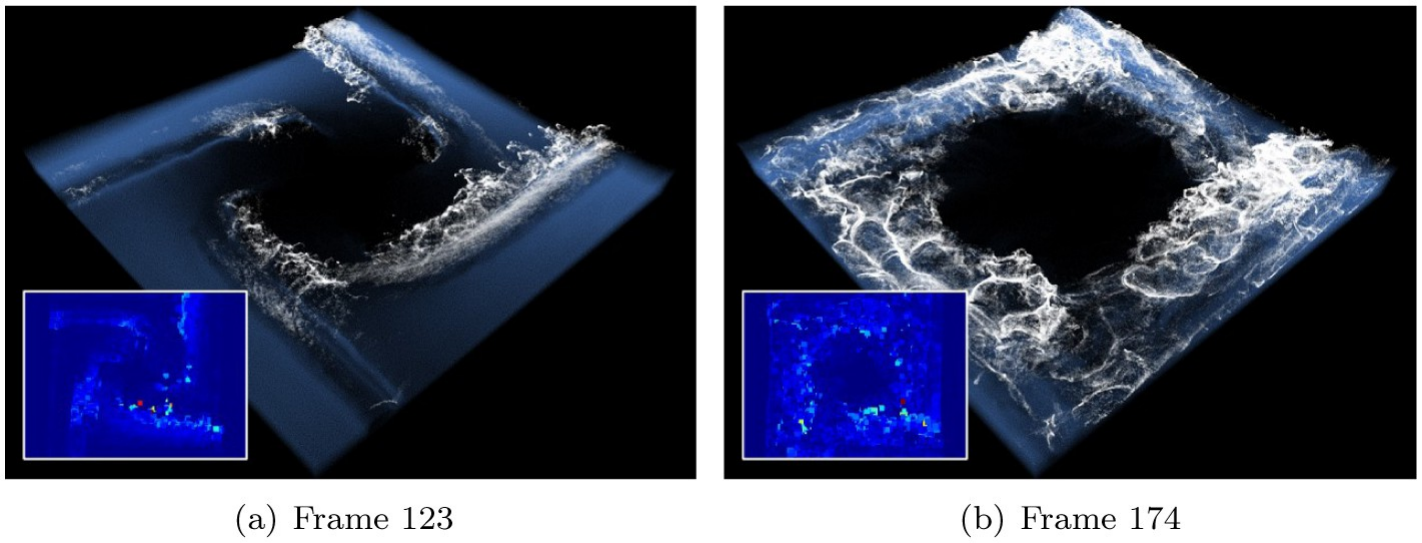
Foam effects with our method in tornado (inset image: Acceleration map). (a) Frame 123, (b) Frame 174.

Figs 13 and 14 show the foam effect generated by water-solid collision or impact between water particles expressed using the emitter. Conversely, Fig 15 is a scene in which foams are gradually generated. Similar to previous results, foam effects are stably expressed by the smoothly refined projection map.

**Fig 16:**
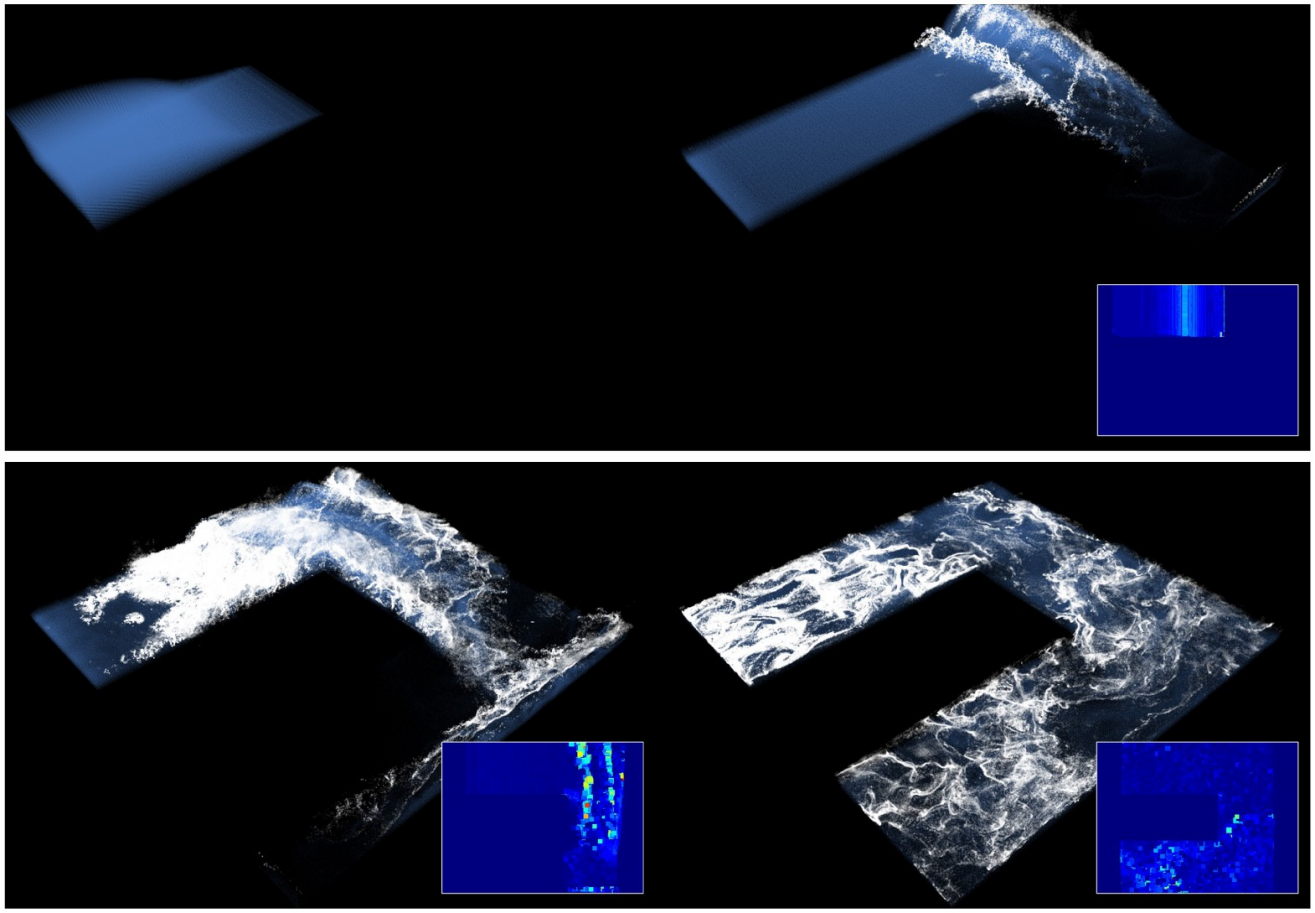
Foam effects by fluid-solid interaction using our method as the water flows along the U-shaped corridor (inset image: Acceleration map).

Fig 16 shows the foam effect expressed in the U-shaped corridor scene, where the foam is stably produced even in the collisions between particles and in the curved paths after the collisions. In particular, through the refined projection map, small-sized foam particles are not lost and are perfectly expressed.

**Fig 17:**
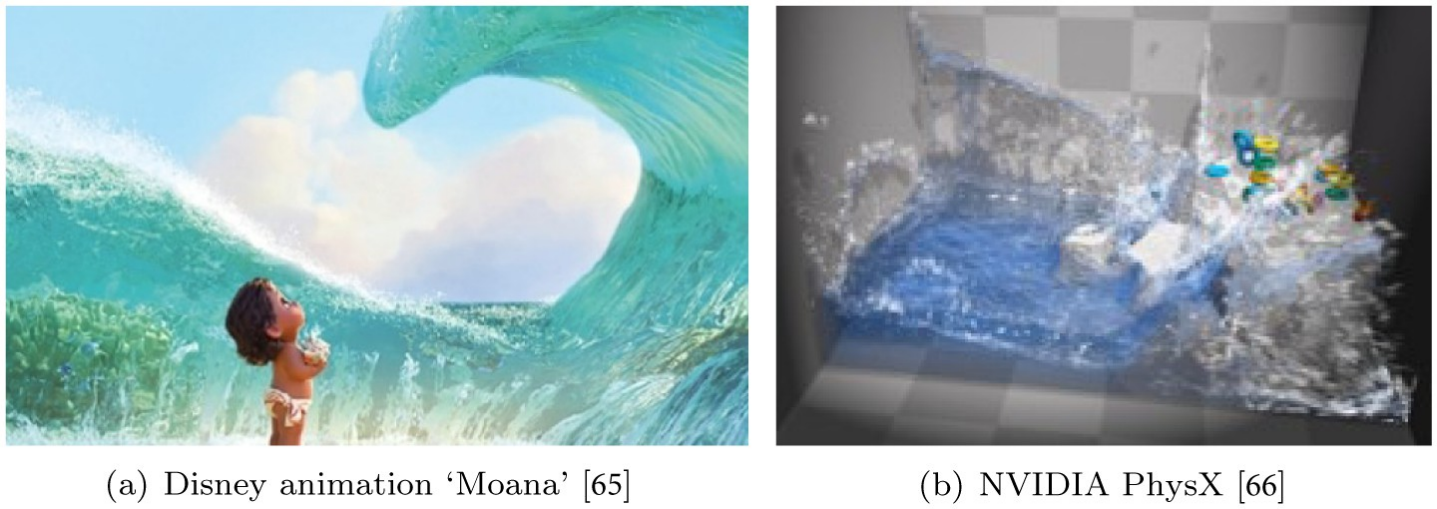
Examples used in film and game. (a) Disney animation ‘Moana’ [65], (b) NVIDIA PhysX [66].

Because the proposed method generates high-quality foam in a short time, it can be used for both high-quality content movie/animation VFX and visual effects used in games, which are real-time contents (see Fig 17). In addition, it is highly capable of being used for building physically based mixed reality in mixed reality environments and the metaverse. Fig 17A is the result showing the water and foam effects expressed in “Moana,” a Disney animation, and Fig 17B is the product of PhysX, a real-time physics engine provided by NAVIDA as open source software. It is used often, unlike Fig 17A, in games to provide real-time contents. Our method is highly applicable because its quality and computational speed are acceptable in both real-time and non-real-time contents. Our method can be used for immersive contents because it allows us to create not only VR/AR but also physically based visual special effects, game, and metaverse spaces based on physics. This shows that our method is expected to contribute to digital contents, mixed reality, the metaverse industry & market, and the content industry.

### Comparison with existing denoising networks

In this section, the method proposed in this study and the existing image digitizing techniques are compared. Because we created foam effect based on a projective approach, various image denoising techniques proposed in the field of computer vision can be applied. The application and implementation are simple; hence, the denoising stages for the projection map described above can be solely replaced with the previous methods. Variational denoising networks) [67] and dual adversarial network (DANet) [68] are compared. Learning is performed using the learning data and algorithms presented in each method, and denoising results are obtained by employing the projection map calculated using fluid particles in the test process as an input. The output denoising map is used to generate foam particles in 3D space through inverse-transformation as described above, and the final result of the foam is compared in this process.

#### Rotating two boxes in water

**Fig 18:**
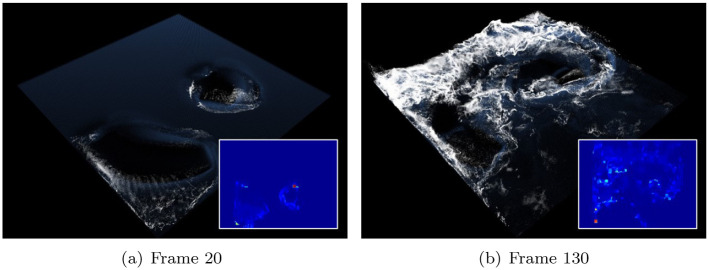
Foam effects with previous method and same scene as Fig 13 (inset image: Refined acceleration map with DnCNN [50]). (a) Frame 20, (b) Frame 130.

Fig 18 shows the results of applying the denoising CNN (DnCNN) [50], a network that performs image denoising based on Deep CNN, for foam simulation. Although noise is alleviated compared to the input acceleration map, the foam detail is damaged because most of the small-sized foams are lost.

**Fig 19:**
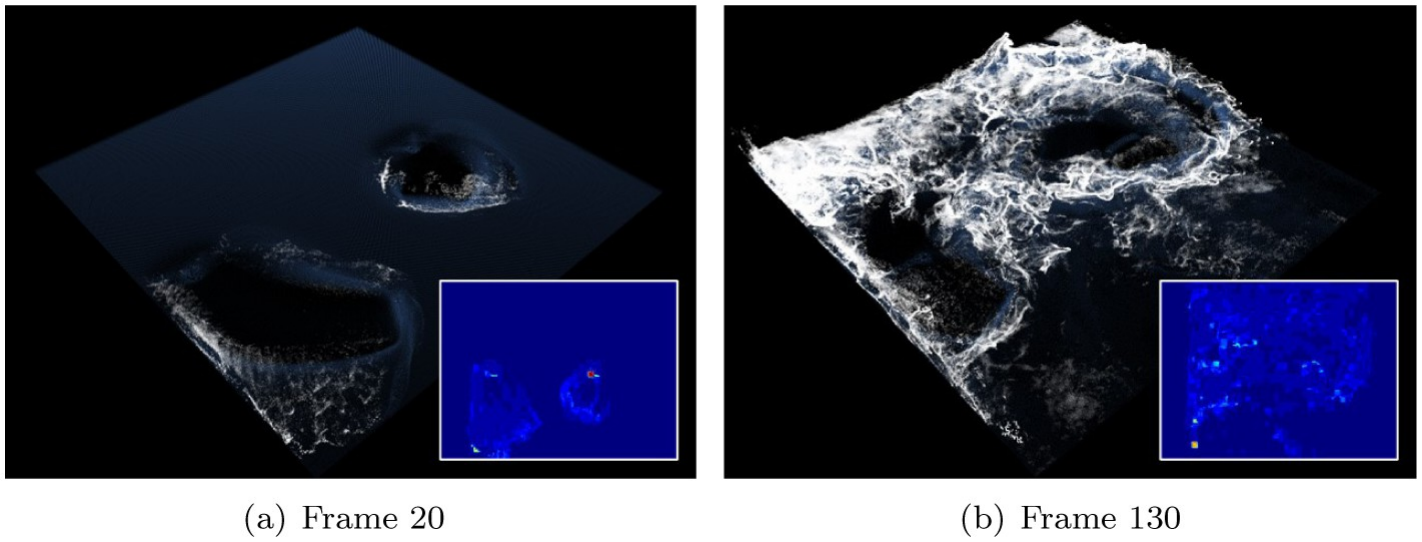
Foam effects with previous method and same scene as Fig 13 (inset image: Refined acceleration map with DANet [68]). (a) Frame 20, (b) Frame 130.

The dual adversarial network (DANet) [68], an image denoising technique, is applied to foam simulation and compared with the proposed method (see Fig 19). Similar to the previous results, foam loss is experienced.

**Fig 20:**
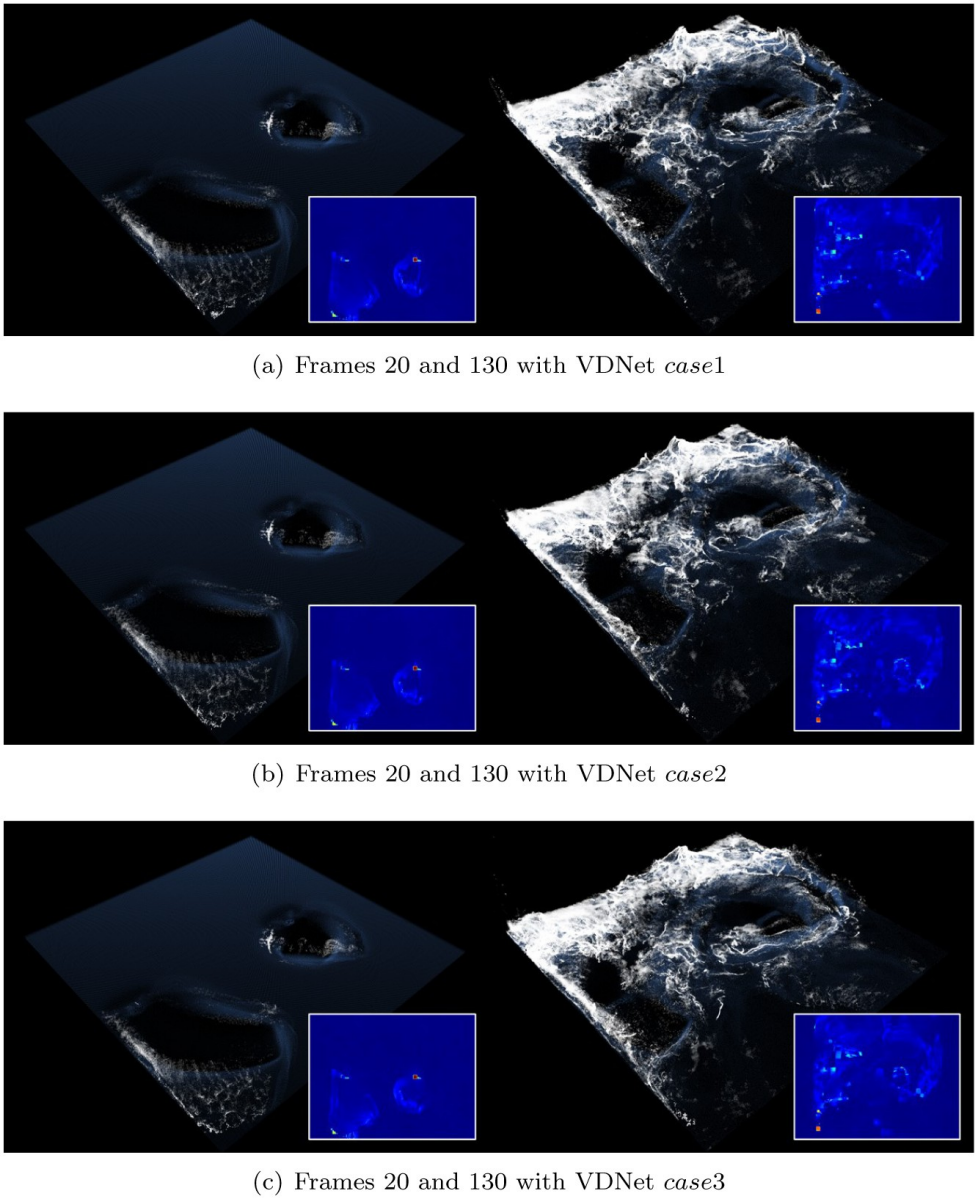
Foam effects with previous method and same scene as Fig 13 (inset image: Refined acceleration map with VDNet [67]). (a) Frames 20 and 130 with VDNet case1, (b) Frames 20 and 130 with VDNet case2, (c) Frames 20 and 130 with VDNet case3.

The last image denoising technique to be compared is variational denoising networks (VDNets) [67] (see Fig 20). This technique utilizes the variance map generated using a Gaussian kernel and performs denoising by applying three different variance maps. In this study, all experiments were conducted for denoising corresponding to *case*1 ∼ 3 for comparing the results of foam effects using VDNet. As mentioned in the original study on VDNet, denoising results were produced according to various test cases and there was no significant difference in foam effects, although a problem of dissipation in surface foam was noted.

**Fig 21:**
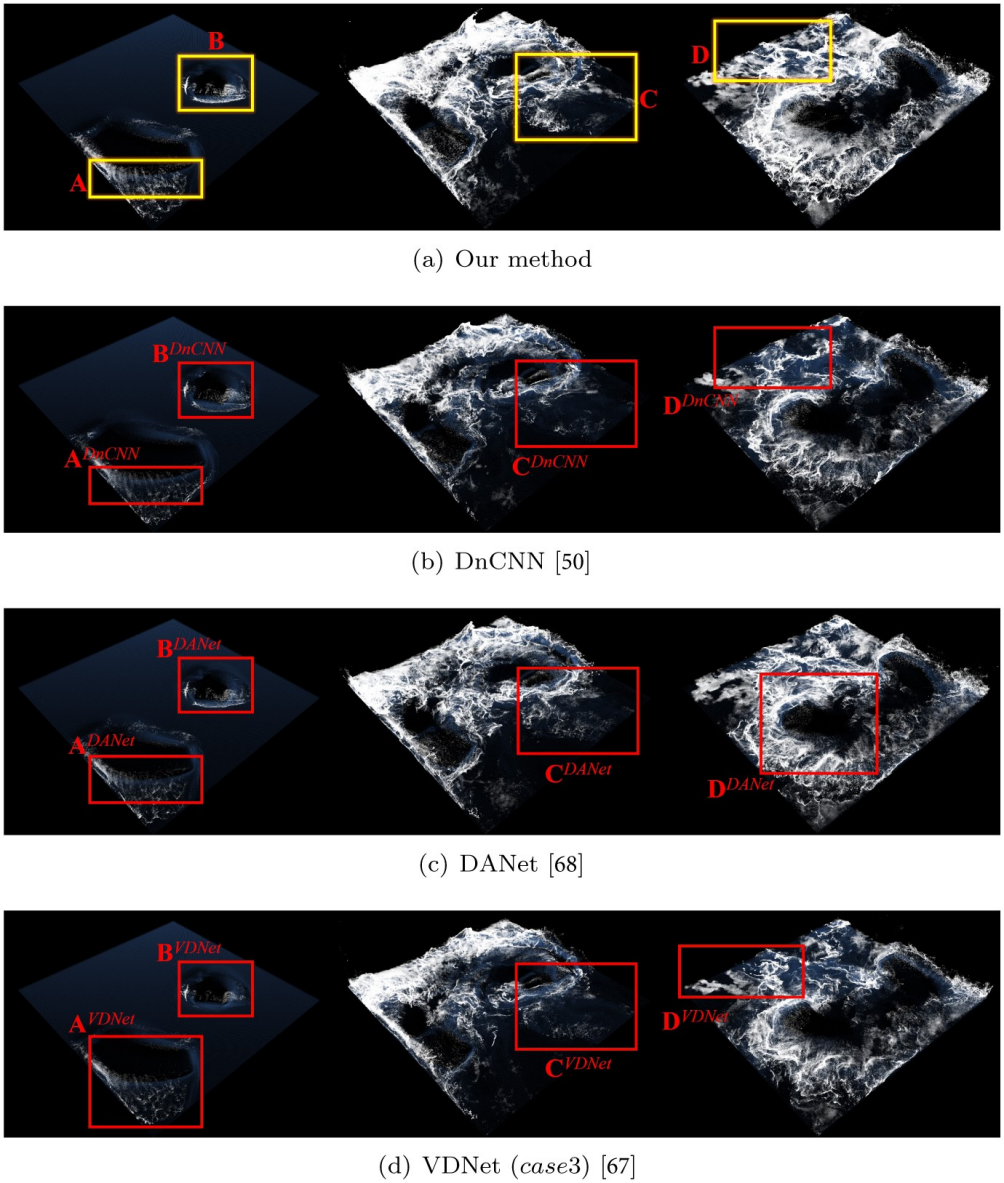
Comparison results with our method and previous approaches (*scene: Rotating two boxes in water*). (a) Our method, (b) DnCNN [50], (c) DANet [68], (d) VDNet (case3) [67].

Fig 21 shows a comparison by frame between the foam effects presented above. Our denoising network showed excellent overall performance without missing details even with small scale foams (see Fig 21A). In the foam effects generated by the initial frame of the box rotation, the proposed method showed intrinsic foam patterns correctly while previous approaches showed loss of foam detail (see **A**^*DnCNN*^, **A**^*DANet*^ and **A**^*VDNet*^ in Fig 21). Similarly, the previous methods in region **B** generated results with lost foam effects, particularly in **B**^*VDNet*^, which generated significant lost foam effects. *case*1 showed diminutive denoising effects in the VDNet experimental results, and *case*3 had a noticeable result. However, the foam effect was severely lost, as shown in **B**^*VDNet*^.

*case*1 showed diminutive denoising effects in the VDNet experimental results, and *case*3 showed a noticeable result. However, the foam effect was severely lost, as shown in **B**^*VDNet*^.

Similarly, the previous methods in region **B** generated results with lost foam effects, particularly in **B**^*VDNet*^, which generated significant lost foam effects.

Foam effects should be accurately visualized not only in the interaction between water particles and solids, but also in the wave crest formed by the interaction between water particles. The proposed method, as shown in **C** and **D**, expressed the foam effects in the wave crest excellently without loss, while most of the previous methods showed damaged foam effects.

**Fig 22:**
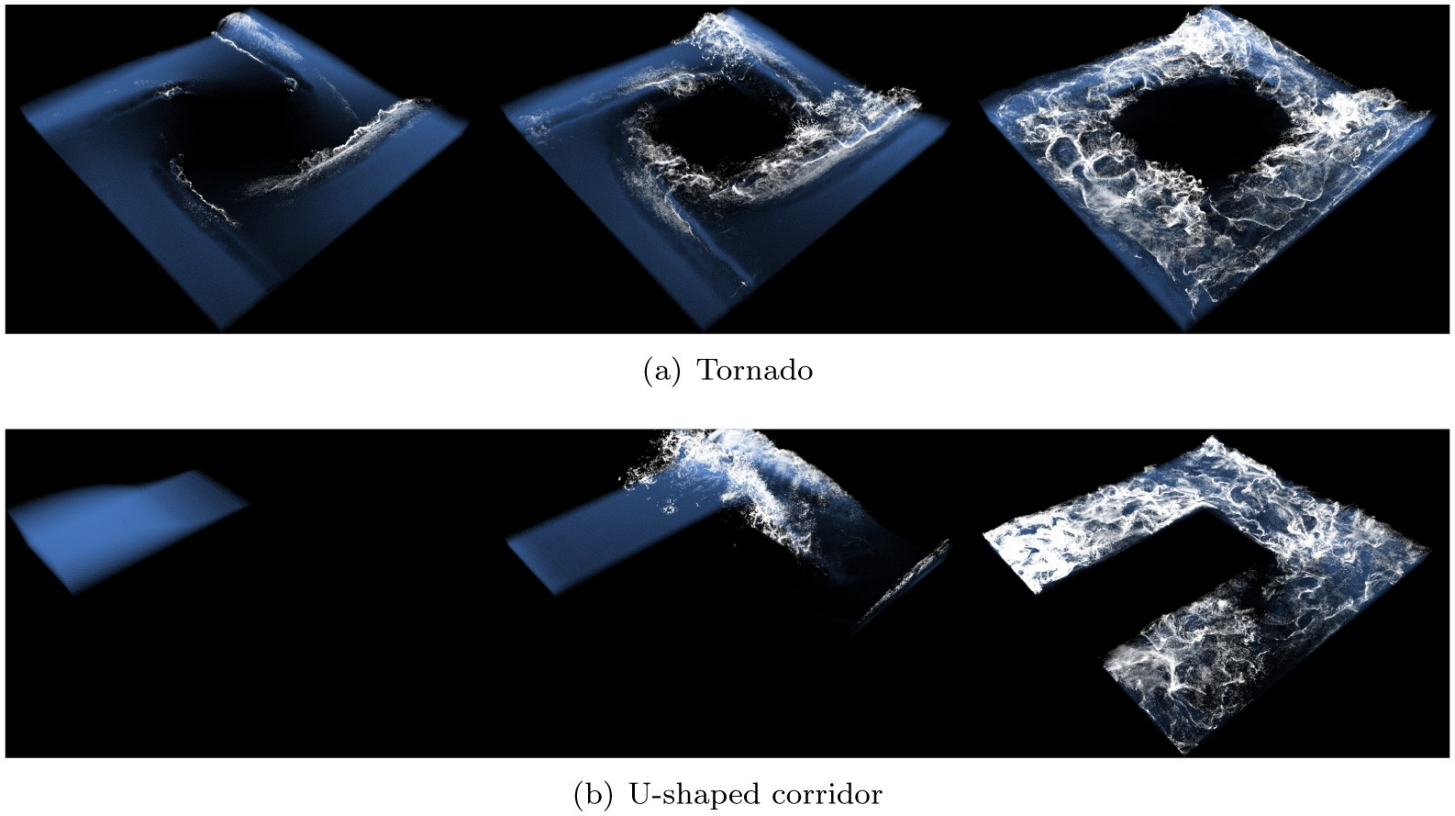
Foam effects with our method. (a) Tornado, (b) U-shaped corridor.

To evaluate the superiority of the proposed method in various scenes as well as the foam effects expressed by the interaction between water particles and solids, we tested foam effect with User-defined tornado external forces (see Fig 22A) and fluid-solid interaction scenes (see Fig 22B) In creating tornado scene, external force was applied as follows: $F\left(x,y,z\right)=\frac{\left(-y,x,0\right)}{{\left(x^{2}+y^{2}\right)}^{2}}$. As shown before, all the results are produced stably without loss of foam effects.

**Fig 23:**
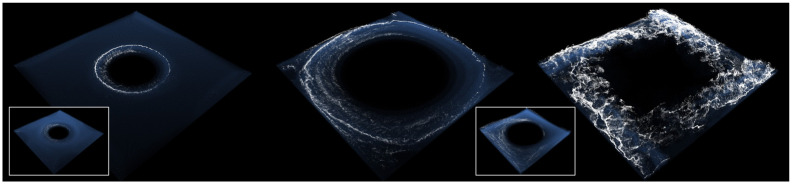
Experimental results by applying our method to the angular advection method of foam particles [69] (inset image: Without angular momentum based advection [22, 23]).

Fig 23 shows the recently introduced foam effects technique using angular advection [69]. The application of our proposed denoising networks to the angular advection technique, where rotation is expressed gradually, leads to foam effects being visualized without loss, even in band-shaped foams expressed as thin foam particles. Compared to the previous techniques in which viscous foam effects are obtained when only linear momentum is applied (see inset image in Fig 23), our proposed method produces foam effects with insignificant loss notwithstanding the denoising process.

Table 3 lists the parameters used to produce the experimental results in this study. The superscripted (*A*)∼(*C*) in Table 3 clearly shows the quantitative characteristics of the proposed method. As shown in the above-mentioned results, most of the previous techniques had the problem of loss of foam effects, and this characteristic was also observed in (*A*). Notwithstanding the same scene configuration, our proposed method contained the largest number of foam particles, compared to that in previous methods. This characteristic was also found in (*B*), showing that more dissipation problems occurs in previous methods. The dissipation problem was more serious in water motion with relatively less sloshing than those with large sloshing, and the water particles with small motion were more dissipated in the existing technique. The same result was also obtained in (*B*), and compared with the previous methods, the difference in the number of foam particles was larger than (*A*). (*C*) is the result of applying the proposed technique to the angular momentum-based foam advection technique [69], which, in the same tornado scene, produced fewer foam particles Fig 22A. This feature is due to the phase change of surface foam to wave foam due to angular advection, not dissipation due to our denoising networks. Nevertheless, our technique expressed the characteristics of angular momentum-based advection excellently and showed a more relaxed foam dissipation problem than the previous technique. The foam effect in the form of a circular band due to the external force on the tornado was also expressed excellently without dissipation.

**Table 3:** Size of our example scene (Water: Water particles, Foam: Foam particles, Solid: Triangles of the solid, Grid res.: Grid resolution).

Figure	Water	Foam	Solid	Grid res.	Projective space res.	Projective spacing
21	1.7 m	^(*A*)^ 21A: 0.6 m	48	150^3^	400×300	2.0
	1.7 m	21B: 0.8 m	48	150^3^	400×300	2.0
	1.7 m	21C: 0.5 m	48	150^3^	400×300	2.0
	1.7 m	21D: 0.6 m	48	150^3^	400×300	2.0
24	2.5 m	^(*B*)^ 24A: 1.1 m	–	150^3^	400×300	2.0
	2.5 m	24B: 1.8 m	–	150^3^	400×300	2.0
	2.5 m	24C: 1.4 m	–	150^3^	400×300	2.0
	2.5 m	24D: 1.1 m	–	150^3^	400×300	2.0
22A	1.7 m	21B: 0.7 m	–	150^3^	400×300	2.0
22B	1.2 m	21B: 1.6 m	70	150^3^	400×300	2.0
23	1.7 m	^(*C*)^ 21B: 1.2 m	–	150^3^	400×300	2.0

**Fig 24:**
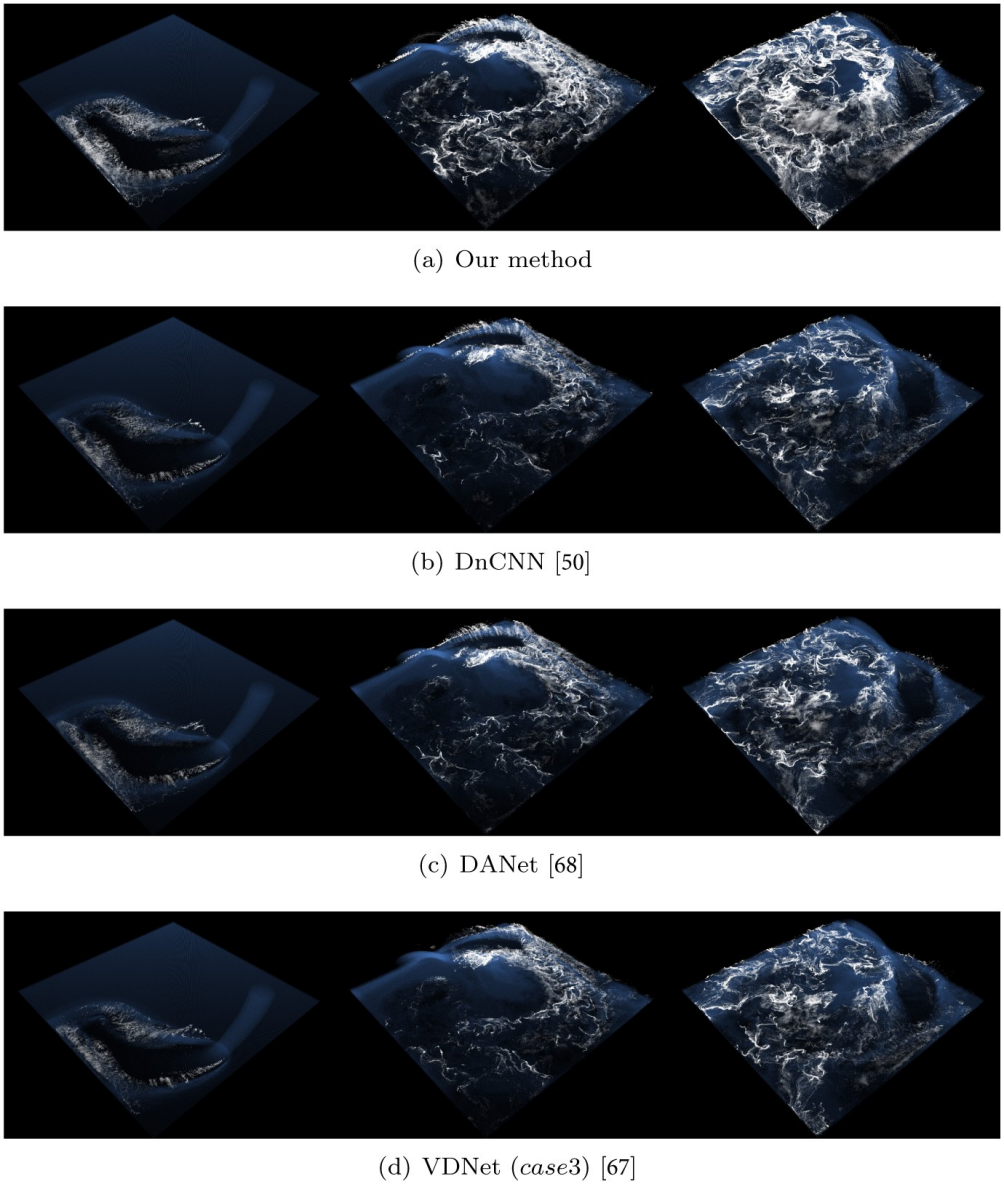
Comparison results with our method and previous approaches (*scene: Rotating*-*emitter in water*).

### Continuity and foam generation comparisons according to denoising

This section compares and analyzes the frequency of flickering by comparing the continuity of the acceleration map with time and the foam particle generation generated in a single frame.

#### Comparison of continuity of acceleration maps over time

As mentioned earlier, our method offered improvement by minimizing foam dissipation. However, because the animation data is similar to time series data, not only loss but also flickering problems occurred. This section compares the frequency of flicking problems in our method, DnCNN [50], DANet [68], and VDNet [67] approaches. To calculate the flickering level between frames, we simply measured the difference between frames as follows: $\frac{\|f_{t}-f_{t+\Delta t}\|}{\Delta t}$.

**Fig 25:**
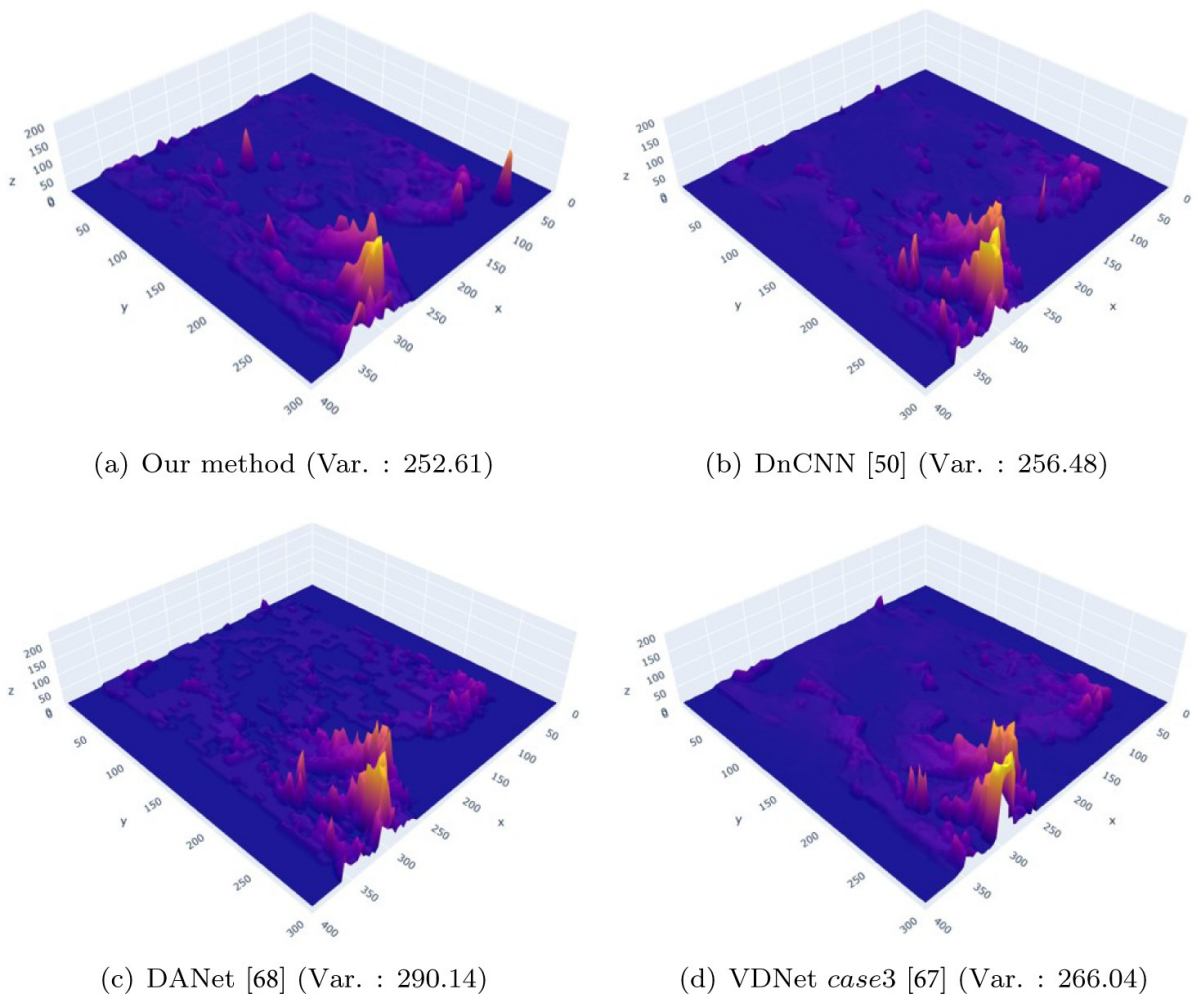
Difference in acceleration map between frames (at frame 160). The chart ranges for each method are as follows: our method, DnCNN, and VDNet→0(■)∼180(■), DANet→0(■)∼200(■)). (a) Our method (Var.: 252.61), (b) DnCNN [50] (Var.: 256.48), (c) DANet [68] (Var.: 290.14), (d) VDNet case3 [67] (Var.: 266.04).

Fig 25 shows the comparison results obtained by expressing the continuity between frames as a terrain mesh. DANet [68] demonstrated a wide range of values compared to other methods, and noisy patterns were found even in the violet color (see Fig 25C). In the case of DnCNN and VDNet, the violet color region appears to be relatively denoised; however, this is the result of over-smoothing, and this characteristic, described in the Result section, leads to loss of foam effects. By contrast, the proposed method not only excellently expressed the peak region in the orange color but also produced denoising results without over-smoothing in the violet color. This characteristic, described in the Result section, leads to complete foam effects without dissipation. In addition, variance was calculated to check the seriousness level of flickering in a single frame, and this result also confirmed that our method is the best variance (see Var. in Fig 25).

#### Comparison of foam generation in a single frame

**Fig 26:**
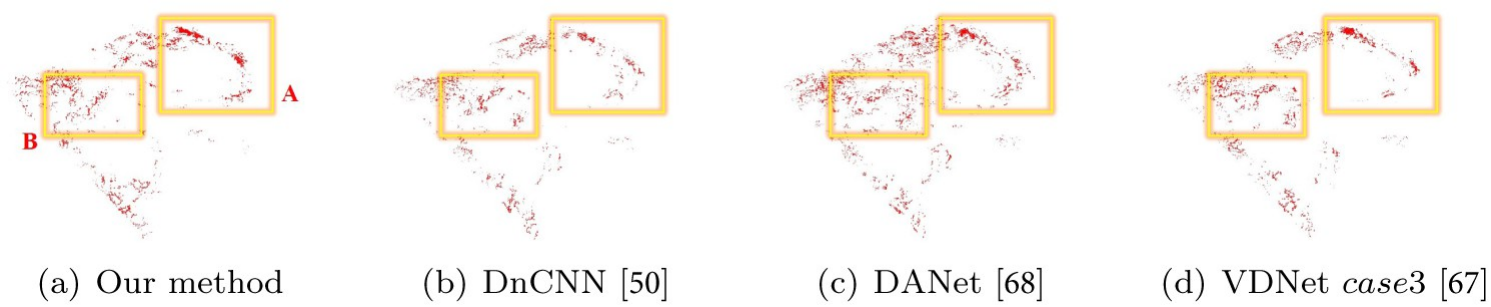
Comparison of foam particles generated in a single frame (at frame 136). (a) Our method, (b) DnCNN [50], (c) DANet [68], (d) VDNet case3 [67].

Fig 26 shows a comparison of the flickering frequency through analysis of foam particles generated in a single frame and density comparison of foam particles for the purpose of observing loss information of foam particles. Comparison results of foam particles generated in successive frames showed that the proposed method had fewer flickering problems with time than previous methods (see the resulting video submitted as S1 Video). Considering a single frame, there was no difference in the amount and location of foam particle generation between proposed method and VDNet (see **A** in Fig 26), and in DnCNN [50] and DANet [68]; dissipation also occurred in the single frame. Similar to the 3D chart shown above, DnCNN [50], in contrast to the proposed method, shows dissipation even in a single frame (see Figs 25B and 26B). In the case of DANet [68], although it seems there is no dissipation, at first glance (see Fig 26C), almost no denoising effects are applied, so noise is expressed without mitigation, and this pattern is also observed in Fig 25C.

**Fig 27:**
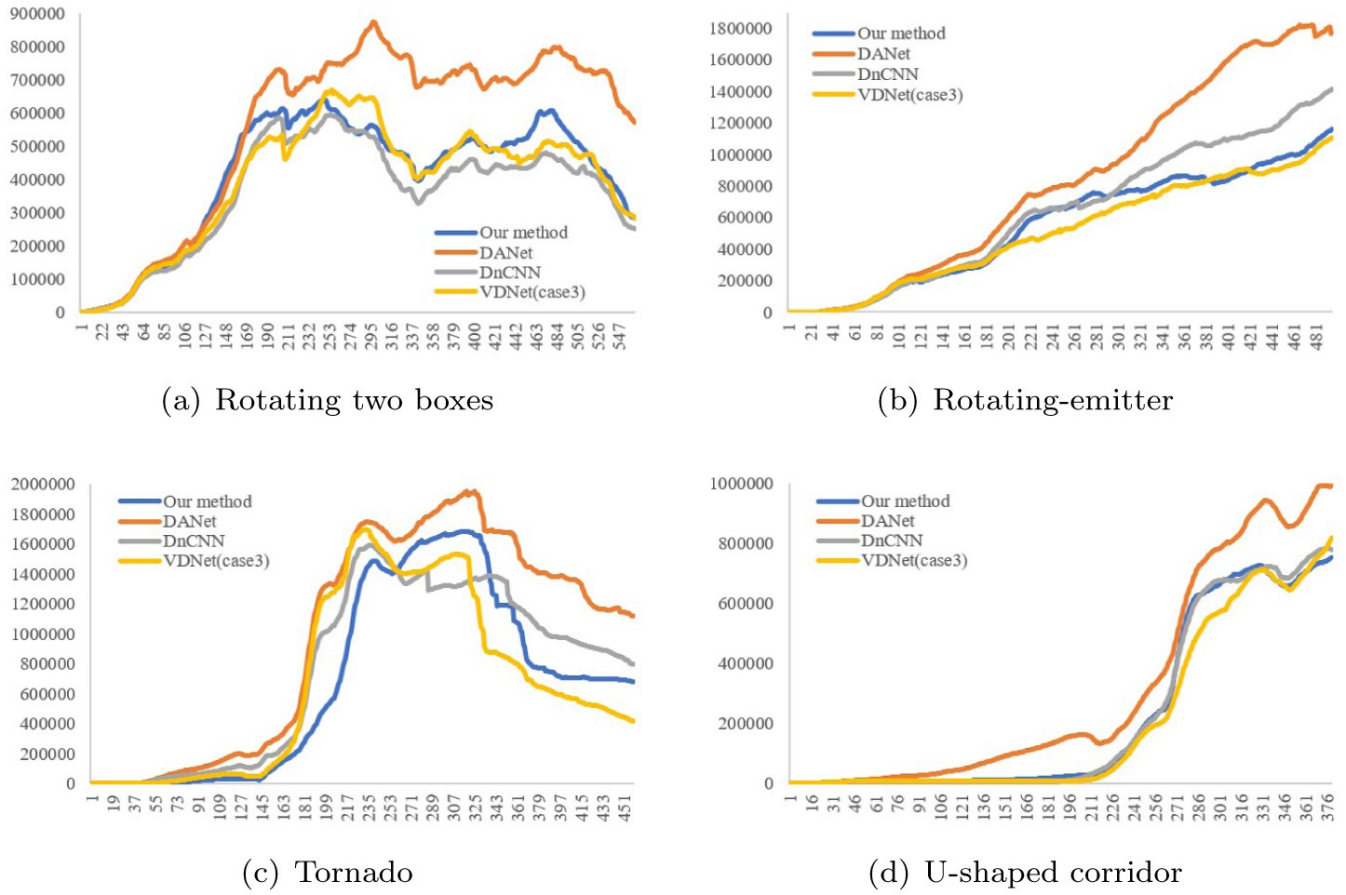
Comparison of foam particles (number) between the previous methods and our method. (a) Rotating two boxes, (b) Rotating-emitter, (c) Tornado, (d) U-shaped corridor.

Fig 27 shows the results of comparing the number of generated foam particles by frame between previous methods and the proposed method. The proposed method produced more foam particles compared to VDNet [67] because it removes only noisy particles, not all particles. This result indicates the effect of the proposed denoising solver on the number of foam particles, and shows clearly that it generates foam effects in more detail than previous methods (see Figs 20 & 23).

Table 4 compares the numbers of foam particles generated in each scene. Max denotes the maximum number of foam particles in that frame and Avg. denotes the average number in each frame. As shown in the table, the number of foam particles in the proposed method is higher or lower than that of previous methods because it removes only noisy foam particles. The pattern of this trend varies by the complexity of the scenes.

**Table 4:** Comparison of foam particles (number).

Figure	Type	Our method	DANet	DnCNN	VDNet
21	Max	639,024	873,029	593,352	667,256
	Avg.	414,053	558,953	360,519	395,710
24	Max	639,024	873,029	593,352	667,256
	Avg.	414,053	558,953	360,519	395,710
22A	Max	753,895	994,551	783,797	817,981
	Avg.	221,483	311,377	221,850	202,904
22B	Max	1,683,396	1,950,685	1,590,905	1,696,093
	Avg.	674,976	977,552	753,513	662,590

### Integration and extensibility with screen projection methods

This study adopted the screen-space method among various foam generation algorithms, and this section describes the reason and possibility of extension. Most foam particle generation methods require access to all grid or particle momentum to analyze the motion of the underlying fluids. This process is also necessary for the Eulerian and Lagrangian approaches. Considering that the foam has feature of being generated on the fluid surface, compared to bubble formed in water, this study used a technique to generate foam particles by projecting fluid flow onto the screen-space [22, 23]. In this method, the foam quality is determined by the quality and resolution of the 2D projection map, making it to be compatible with the proposed method. Although the goal in designing algorithm was foam generation, we expect it to be applicable to games and real-time applications that mainly use screen-space rendering techniques [20, 32, 70, 71].

Image denoising is still receiving active academic interest. Recently, Xu et al. used class-specific convolution to efficiently solve the denoising problem based on deep learning [72]. In addition, Wang et al. proposed a method to practically process image denoising in mobile devices [73]. Their proposed method can be used in the 2D projective space approach of this study because it alleviates the noise problem from a practical point of view rather than detail. Recently, Lin et al. dealt with the image denoising problem using adaptive & overlapped average filtering [74]. This method is similar to ours as it calculates a non-noisy map from an image based on the adaptive filtering method and the small-scale details are preserved in most of the results. However, the image denoising implemented in the 2D image is insufficient for a complete foam denoising solution, as discussed in this study. However, the utilization of overlapped average filtering is expected to improve the robustness of the proposed method. There are various types of foam, all with various characteristics, such as wave foam that oscillates strongly according to the underlying motion and surface foam that floats steadily. The adaptive and overlapped average filter can be an excellent candidate to classify and stably learn these characteristics. As Lin et al. demonstrated that the performance of their method is sufficient based on images, their method is expected to be further improved through integration with our method in the future.

## Conclusions and future work

This study proposed a screen projection method and denoising network architecture that efficiently express foam generation in water simulations. To refine the projection map, data were collected in the preprocessing process, and a method to mitigate noise using a residual-based network was introduced. Using the proposed network, Z*, D*, and F* that affect foam formation were newly calculated.

We obtained and analyzed the results in terms of visual aspects to minimize foam loss and evaluated the denoising performance. For physics-based simulations in computer graphics, the visual quality is as important as numerical accuracy. As in the Problem Statement, where the problem is defined in the visual aspect and a solution is presented to solve it, we focused on comparison in terms of visual aspect in the Result analysis and Discussion sections. We believe that the comparison of projective spaces (Fig 25), instead of quantitative comparison of the numbers of foam particles, is a reliable method to analyze the results because the foam particles in this study are generated by momentum projection onto the projective space. In addition, the visualization of foam particles generated in a specific frame shows that denoising is more stable and the expression of foam is more detailed in our method than in conventional methods.

Based on experiments in various scenes, it was confirmed that the proposed network method is superior to the sole application of a separable binomial filter in terms of denoising. Existing techniques for image denoising are unable to fully express small-sized bubbles before they are lost. Contrarily, the proposed method in this study can effectively visualize diminutive bubbles stably without dissipation. The proposed method improved the foam quality and efficiency using a network that removes the noise generated in the process of projecting water particles onto the screen. However, it has a limitation in that the 3D momentum is not accumulated accurately in 2D format because only water particles close to the screen are considered in the projection process. We plan to expand the research to include a nonlinear mapping method that reflects the 3D momentum in 2D maps. The successful outcome of this research is expected to be the generation of foam effects even in occlusion regions, which is a weakness of screen space.

The technical contribution of this paper lies not in the discovery of a new network architecture but in the discovery of a new noise problem found in the process of projecting foam effects in 3D onto the projective space and solving it using an image denoising approach. This is not a network-based re-visitation of the previously solved problem, but a new problem raised first in physically based simulation. In addition, as this is the first time representation learning is being performed using the artificial neural network method, we addressed various issues, from how to build a dataset to training/testing in detail. This resulted in superior performance compared with conventional methods. However, from a network point of view, this is not a completely new method because it uses the previous convolutional neural network architecture. The reasons for selecting this type of network for this study include its history of application in many fields, and thus, algorithm integration stability and ease of reproduction by readers are expected. We believe that the proposed method did not completely achieve denoising in foam simulation. There are many types of foam effects depending on the position or motion generated in the underlying fluids. If we fully represent these various foam particles, we believe that the network architecture should be redesigned. In the future, the feature points of the foam particles will be redefined, and a new artificial neural network design is planned.

The VGG-like model has already been used in representation learning. Although our model has a relatively shallow architecture, it was sufficient to achieve the purpose of this study. Our approach can be applied directly to ResNet, a deeper structure, or lightweight EfficientNet; however, there will be almost no difference in performance. In the future, it will be possible to use the popular Transformer-based model or a GNN-based model by focusing on the movement of particles. In future work, we aim to expand this study to include a method to refine the projection map according to the flow level using an adaptive and anisotropic data structure.

## Supporting information

S1 Video Supplementary result data. Related to Figs 21∼24.
(AVI)

S1 Data Training data. The training datas are presented in the Supporting Information.
(ZIP)
