# Supplementary figures and images for: Efficient learning representation of noise-reduced foam effects with convolutional denoising networks (part 2 of 2)
Source: PLoS One. 2022 Oct 10;17(10):e0275117. doi: 10.1371/journal.pone.0275117 (PMC9551625; doi:10.1371/journal.pone.0275117)

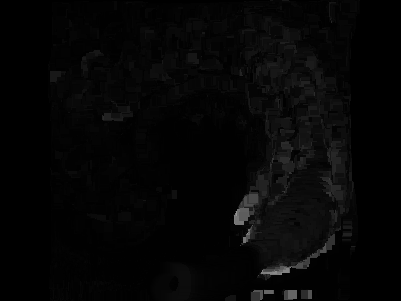

Supplement: S1 Data — The training datas are presented in the Supporting Information. (ZIP) [file pone.0275117.s002.zip › Rotating emitter/with filtering/originAcc-189.bmp]

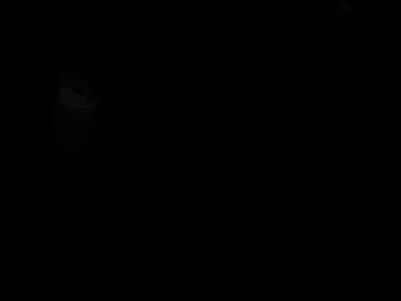

Supplement: S1 Data — The training datas are presented in the Supporting Information. (ZIP) [file pone.0275117.s002.zip › Rotating emitter/with filtering/originAcc-19.bmp]

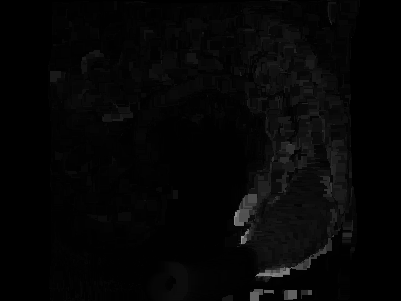

Supplement: S1 Data — The training datas are presented in the Supporting Information. (ZIP) [file pone.0275117.s002.zip › Rotating emitter/with filtering/originAcc-190.bmp]

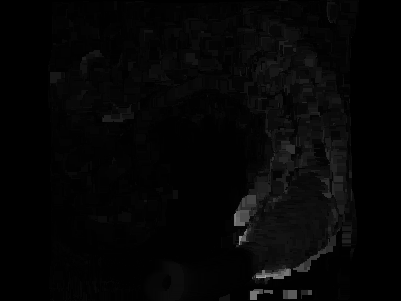

Supplement: S1 Data — The training datas are presented in the Supporting Information. (ZIP) [file pone.0275117.s002.zip › Rotating emitter/with filtering/originAcc-191.bmp]

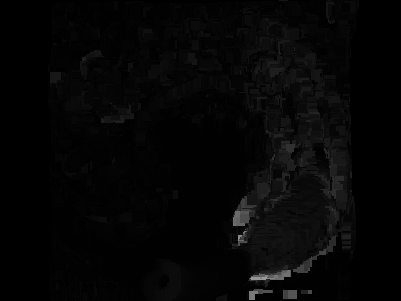

Supplement: S1 Data — The training datas are presented in the Supporting Information. (ZIP) [file pone.0275117.s002.zip › Rotating emitter/with filtering/originAcc-192.bmp]

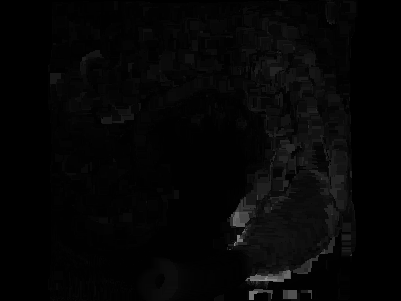

Supplement: S1 Data — The training datas are presented in the Supporting Information. (ZIP) [file pone.0275117.s002.zip › Rotating emitter/with filtering/originAcc-193.bmp]

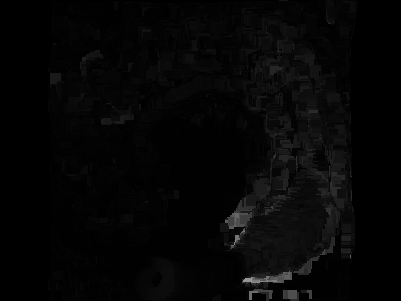

Supplement: S1 Data — The training datas are presented in the Supporting Information. (ZIP) [file pone.0275117.s002.zip › Rotating emitter/with filtering/originAcc-194.bmp]

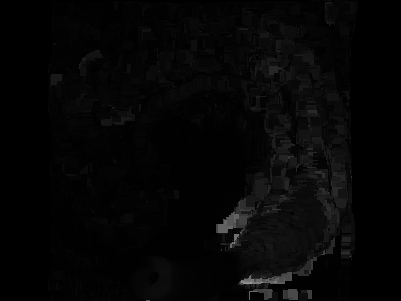

Supplement: S1 Data — The training datas are presented in the Supporting Information. (ZIP) [file pone.0275117.s002.zip › Rotating emitter/with filtering/originAcc-195.bmp]

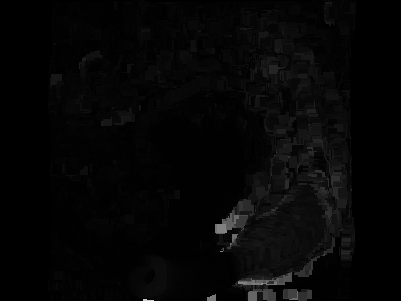

Supplement: S1 Data — The training datas are presented in the Supporting Information. (ZIP) [file pone.0275117.s002.zip › Rotating emitter/with filtering/originAcc-196.bmp]

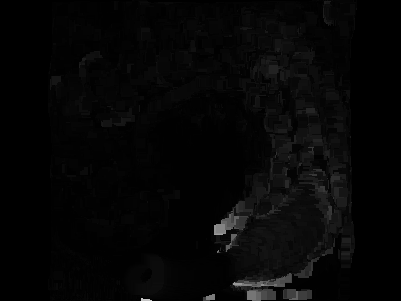

Supplement: S1 Data — The training datas are presented in the Supporting Information. (ZIP) [file pone.0275117.s002.zip › Rotating emitter/with filtering/originAcc-197.bmp]

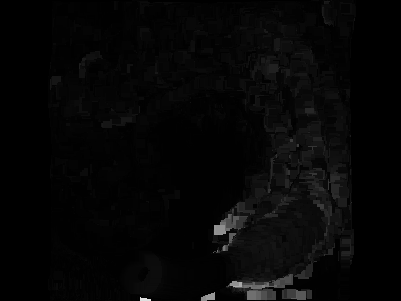

Supplement: S1 Data — The training datas are presented in the Supporting Information. (ZIP) [file pone.0275117.s002.zip › Rotating emitter/with filtering/originAcc-198.bmp]

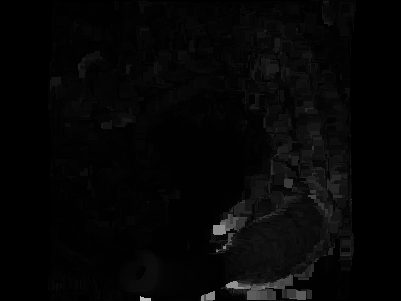

Supplement: S1 Data — The training datas are presented in the Supporting Information. (ZIP) [file pone.0275117.s002.zip › Rotating emitter/with filtering/originAcc-199.bmp]

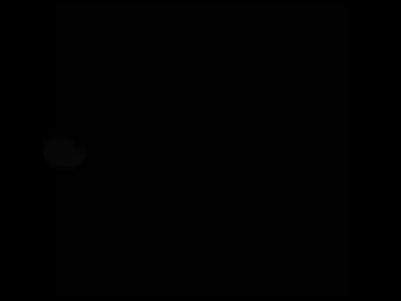

Supplement: S1 Data — The training datas are presented in the Supporting Information. (ZIP) [file pone.0275117.s002.zip › Rotating emitter/with filtering/originAcc-2.bmp]

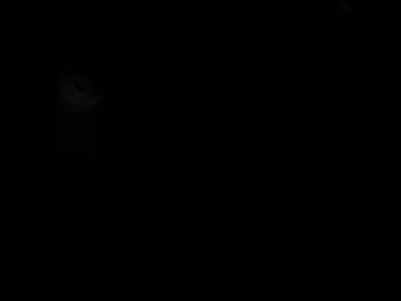

Supplement: S1 Data — The training datas are presented in the Supporting Information. (ZIP) [file pone.0275117.s002.zip › Rotating emitter/with filtering/originAcc-20.bmp]

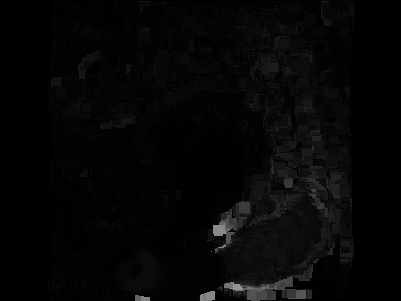

Supplement: S1 Data — The training datas are presented in the Supporting Information. (ZIP) [file pone.0275117.s002.zip › Rotating emitter/with filtering/originAcc-200.bmp]

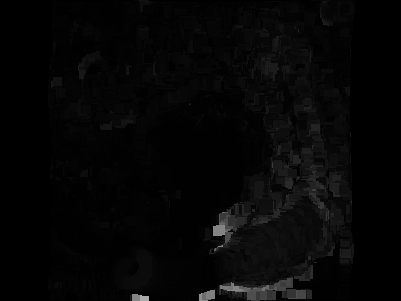

Supplement: S1 Data — The training datas are presented in the Supporting Information. (ZIP) [file pone.0275117.s002.zip › Rotating emitter/with filtering/originAcc-201.bmp]

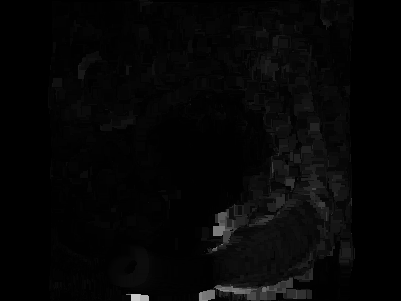

Supplement: S1 Data — The training datas are presented in the Supporting Information. (ZIP) [file pone.0275117.s002.zip › Rotating emitter/with filtering/originAcc-202.bmp]

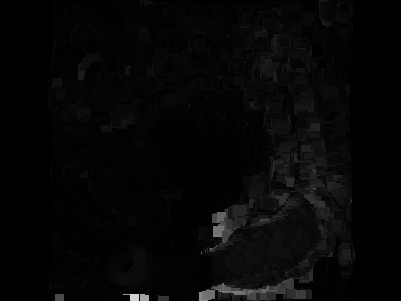

Supplement: S1 Data — The training datas are presented in the Supporting Information. (ZIP) [file pone.0275117.s002.zip › Rotating emitter/with filtering/originAcc-203.bmp]

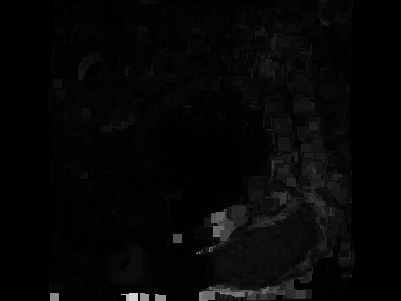

Supplement: S1 Data — The training datas are presented in the Supporting Information. (ZIP) [file pone.0275117.s002.zip › Rotating emitter/with filtering/originAcc-204.bmp]

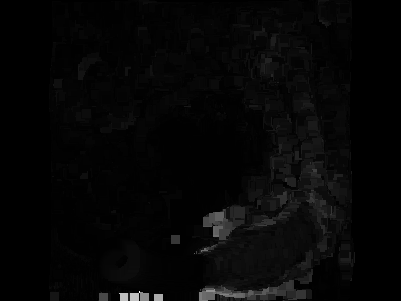

Supplement: S1 Data — The training datas are presented in the Supporting Information. (ZIP) [file pone.0275117.s002.zip › Rotating emitter/with filtering/originAcc-205.bmp]

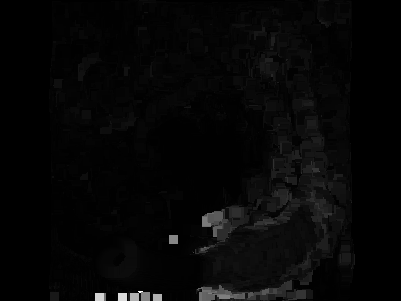

Supplement: S1 Data — The training datas are presented in the Supporting Information. (ZIP) [file pone.0275117.s002.zip › Rotating emitter/with filtering/originAcc-206.bmp]

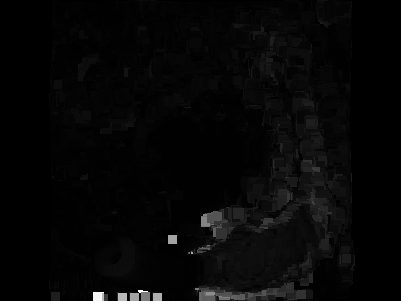

Supplement: S1 Data — The training datas are presented in the Supporting Information. (ZIP) [file pone.0275117.s002.zip › Rotating emitter/with filtering/originAcc-207.bmp]

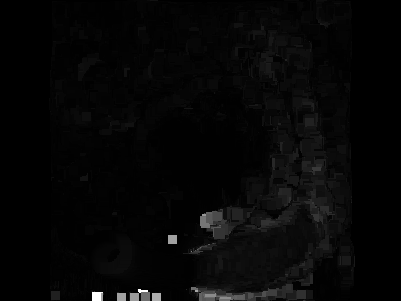

Supplement: S1 Data — The training datas are presented in the Supporting Information. (ZIP) [file pone.0275117.s002.zip › Rotating emitter/with filtering/originAcc-208.bmp]

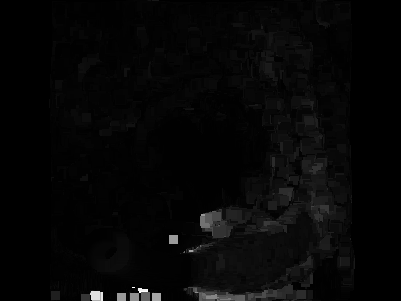

Supplement: S1 Data — The training datas are presented in the Supporting Information. (ZIP) [file pone.0275117.s002.zip › Rotating emitter/with filtering/originAcc-209.bmp]

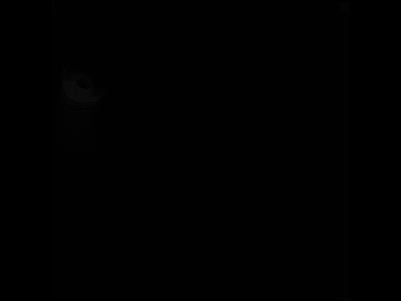

Supplement: S1 Data — The training datas are presented in the Supporting Information. (ZIP) [file pone.0275117.s002.zip › Rotating emitter/with filtering/originAcc-21.bmp]

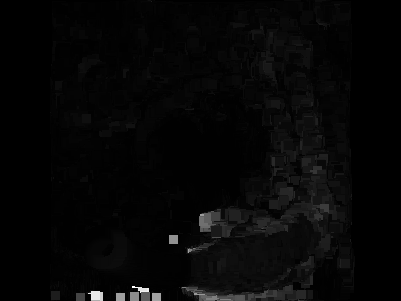

Supplement: S1 Data — The training datas are presented in the Supporting Information. (ZIP) [file pone.0275117.s002.zip › Rotating emitter/with filtering/originAcc-210.bmp]

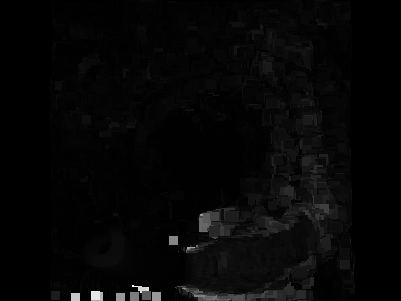

Supplement: S1 Data — The training datas are presented in the Supporting Information. (ZIP) [file pone.0275117.s002.zip › Rotating emitter/with filtering/originAcc-211.bmp]

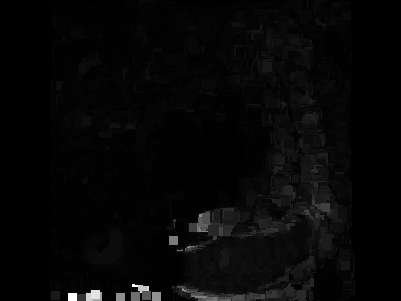

Supplement: S1 Data — The training datas are presented in the Supporting Information. (ZIP) [file pone.0275117.s002.zip › Rotating emitter/with filtering/originAcc-212.bmp]

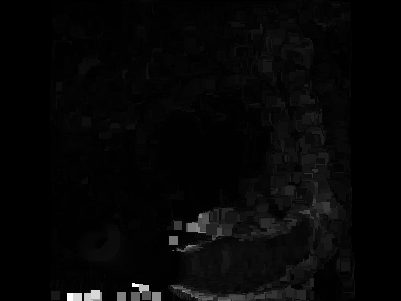

Supplement: S1 Data — The training datas are presented in the Supporting Information. (ZIP) [file pone.0275117.s002.zip › Rotating emitter/with filtering/originAcc-213.bmp]

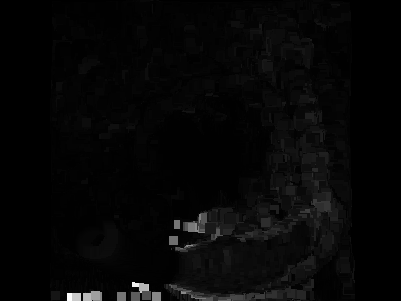

Supplement: S1 Data — The training datas are presented in the Supporting Information. (ZIP) [file pone.0275117.s002.zip › Rotating emitter/with filtering/originAcc-214.bmp]

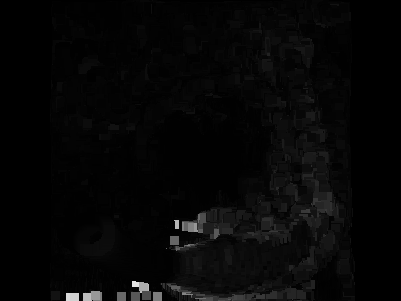

Supplement: S1 Data — The training datas are presented in the Supporting Information. (ZIP) [file pone.0275117.s002.zip › Rotating emitter/with filtering/originAcc-215.bmp]

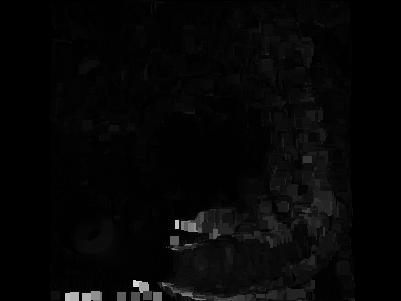

Supplement: S1 Data — The training datas are presented in the Supporting Information. (ZIP) [file pone.0275117.s002.zip › Rotating emitter/with filtering/originAcc-216.bmp]

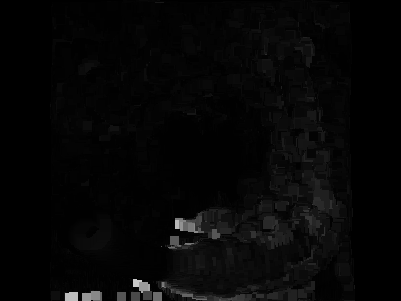

Supplement: S1 Data — The training datas are presented in the Supporting Information. (ZIP) [file pone.0275117.s002.zip › Rotating emitter/with filtering/originAcc-217.bmp]

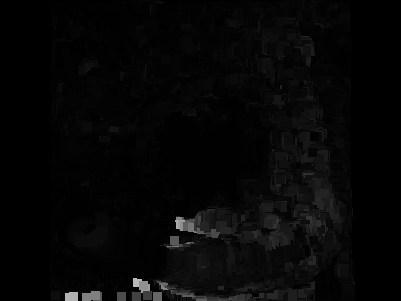

Supplement: S1 Data — The training datas are presented in the Supporting Information. (ZIP) [file pone.0275117.s002.zip › Rotating emitter/with filtering/originAcc-218.bmp]

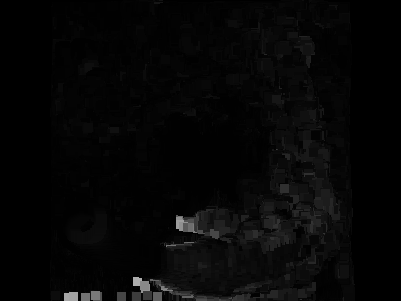

Supplement: S1 Data — The training datas are presented in the Supporting Information. (ZIP) [file pone.0275117.s002.zip › Rotating emitter/with filtering/originAcc-219.bmp]

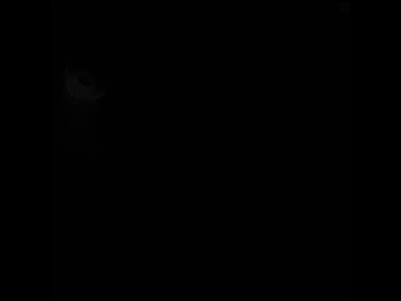

Supplement: S1 Data — The training datas are presented in the Supporting Information. (ZIP) [file pone.0275117.s002.zip › Rotating emitter/with filtering/originAcc-22.bmp]

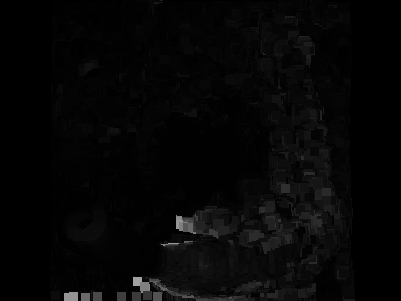

Supplement: S1 Data — The training datas are presented in the Supporting Information. (ZIP) [file pone.0275117.s002.zip › Rotating emitter/with filtering/originAcc-220.bmp]

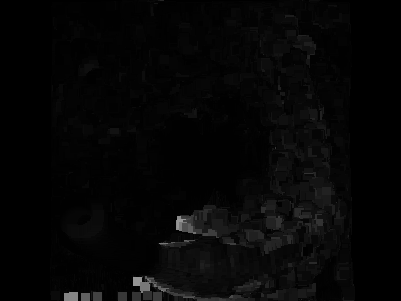

Supplement: S1 Data — The training datas are presented in the Supporting Information. (ZIP) [file pone.0275117.s002.zip › Rotating emitter/with filtering/originAcc-221.bmp]

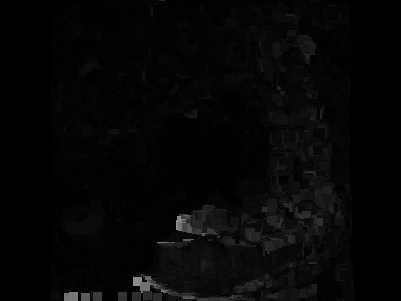

Supplement: S1 Data — The training datas are presented in the Supporting Information. (ZIP) [file pone.0275117.s002.zip › Rotating emitter/with filtering/originAcc-222.bmp]

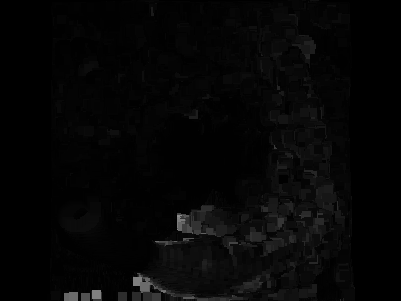

Supplement: S1 Data — The training datas are presented in the Supporting Information. (ZIP) [file pone.0275117.s002.zip › Rotating emitter/with filtering/originAcc-223.bmp]

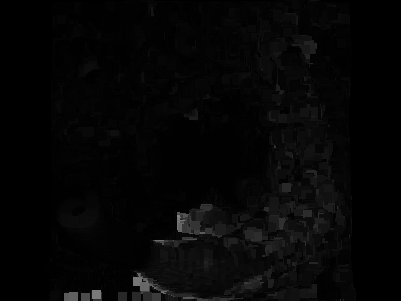

Supplement: S1 Data — The training datas are presented in the Supporting Information. (ZIP) [file pone.0275117.s002.zip › Rotating emitter/with filtering/originAcc-224.bmp]

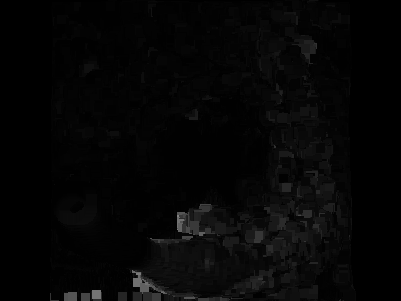

Supplement: S1 Data — The training datas are presented in the Supporting Information. (ZIP) [file pone.0275117.s002.zip › Rotating emitter/with filtering/originAcc-225.bmp]

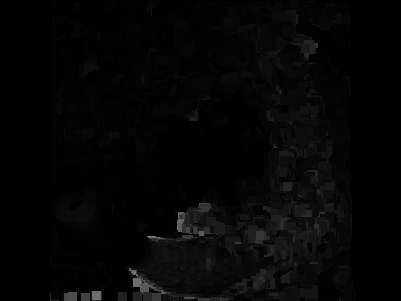

Supplement: S1 Data — The training datas are presented in the Supporting Information. (ZIP) [file pone.0275117.s002.zip › Rotating emitter/with filtering/originAcc-226.bmp]

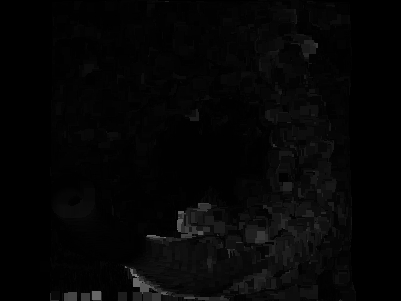

Supplement: S1 Data — The training datas are presented in the Supporting Information. (ZIP) [file pone.0275117.s002.zip › Rotating emitter/with filtering/originAcc-227.bmp]

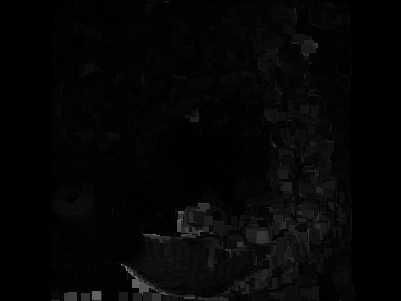

Supplement: S1 Data — The training datas are presented in the Supporting Information. (ZIP) [file pone.0275117.s002.zip › Rotating emitter/with filtering/originAcc-228.bmp]

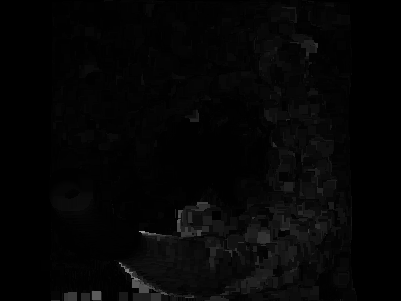

Supplement: S1 Data — The training datas are presented in the Supporting Information. (ZIP) [file pone.0275117.s002.zip › Rotating emitter/with filtering/originAcc-229.bmp]

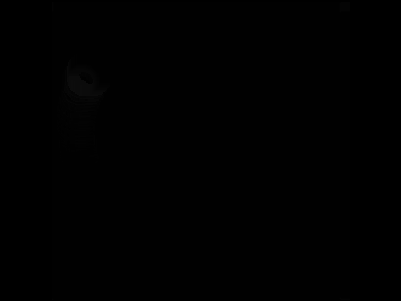

Supplement: S1 Data — The training datas are presented in the Supporting Information. (ZIP) [file pone.0275117.s002.zip › Rotating emitter/with filtering/originAcc-23.bmp]

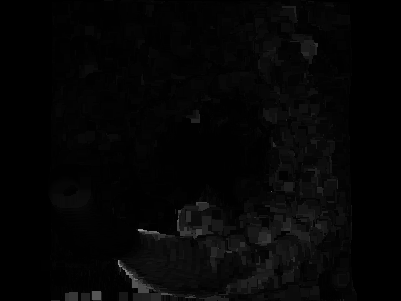

Supplement: S1 Data — The training datas are presented in the Supporting Information. (ZIP) [file pone.0275117.s002.zip › Rotating emitter/with filtering/originAcc-230.bmp]

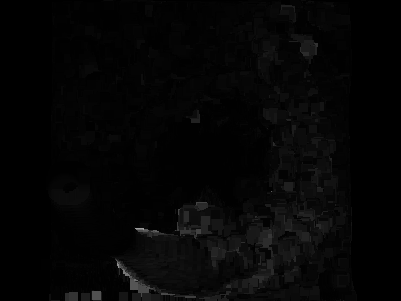

Supplement: S1 Data — The training datas are presented in the Supporting Information. (ZIP) [file pone.0275117.s002.zip › Rotating emitter/with filtering/originAcc-231.bmp]

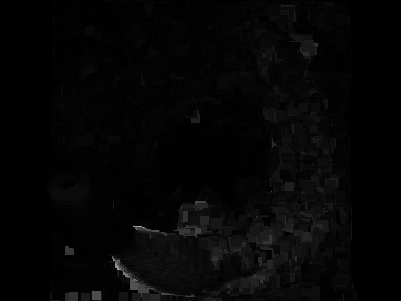

Supplement: S1 Data — The training datas are presented in the Supporting Information. (ZIP) [file pone.0275117.s002.zip › Rotating emitter/with filtering/originAcc-232.bmp]

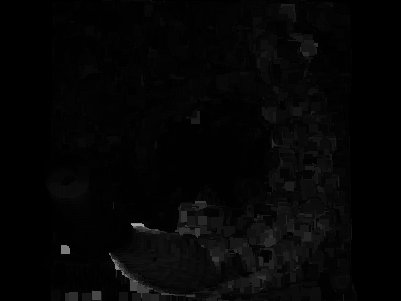

Supplement: S1 Data — The training datas are presented in the Supporting Information. (ZIP) [file pone.0275117.s002.zip › Rotating emitter/with filtering/originAcc-233.bmp]

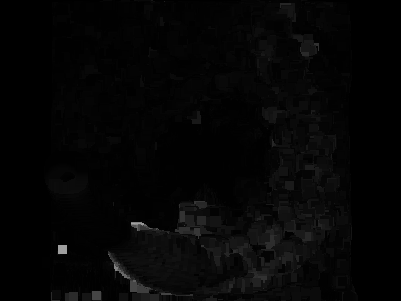

Supplement: S1 Data — The training datas are presented in the Supporting Information. (ZIP) [file pone.0275117.s002.zip › Rotating emitter/with filtering/originAcc-234.bmp]

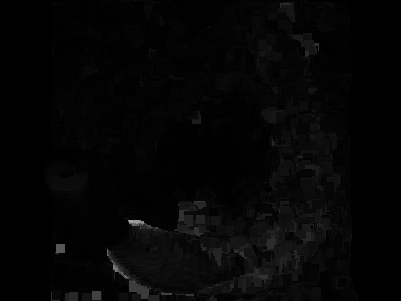

Supplement: S1 Data — The training datas are presented in the Supporting Information. (ZIP) [file pone.0275117.s002.zip › Rotating emitter/with filtering/originAcc-235.bmp]

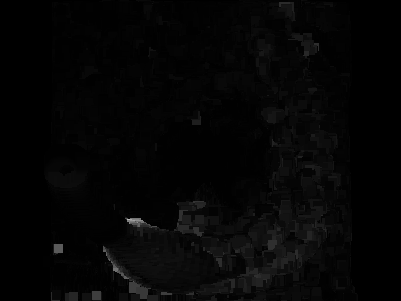

Supplement: S1 Data — The training datas are presented in the Supporting Information. (ZIP) [file pone.0275117.s002.zip › Rotating emitter/with filtering/originAcc-236.bmp]

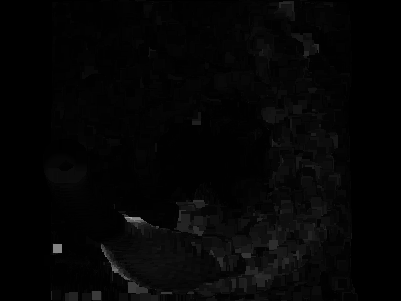

Supplement: S1 Data — The training datas are presented in the Supporting Information. (ZIP) [file pone.0275117.s002.zip › Rotating emitter/with filtering/originAcc-237.bmp]

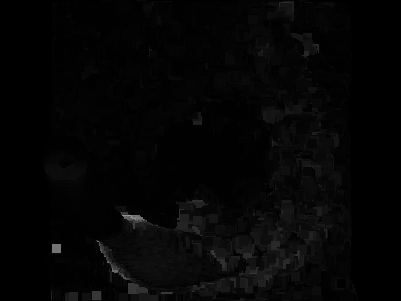

Supplement: S1 Data — The training datas are presented in the Supporting Information. (ZIP) [file pone.0275117.s002.zip › Rotating emitter/with filtering/originAcc-238.bmp]

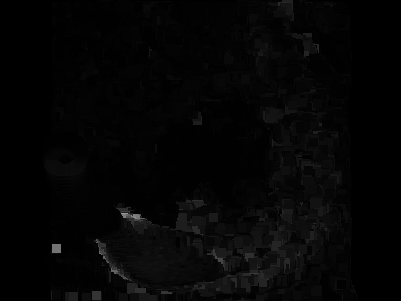

Supplement: S1 Data — The training datas are presented in the Supporting Information. (ZIP) [file pone.0275117.s002.zip › Rotating emitter/with filtering/originAcc-239.bmp]

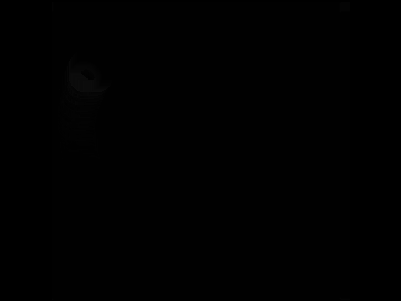

Supplement: S1 Data — The training datas are presented in the Supporting Information. (ZIP) [file pone.0275117.s002.zip › Rotating emitter/with filtering/originAcc-24.bmp]

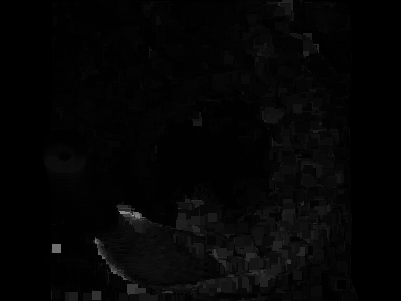

Supplement: S1 Data — The training datas are presented in the Supporting Information. (ZIP) [file pone.0275117.s002.zip › Rotating emitter/with filtering/originAcc-240.bmp]

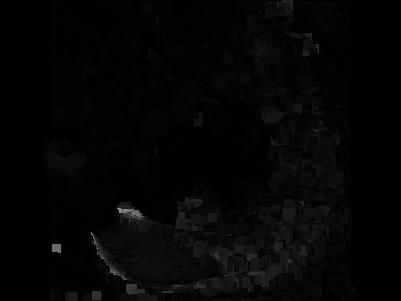

Supplement: S1 Data — The training datas are presented in the Supporting Information. (ZIP) [file pone.0275117.s002.zip › Rotating emitter/with filtering/originAcc-241.bmp]

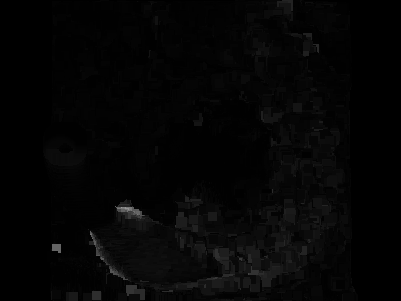

Supplement: S1 Data — The training datas are presented in the Supporting Information. (ZIP) [file pone.0275117.s002.zip › Rotating emitter/with filtering/originAcc-242.bmp]

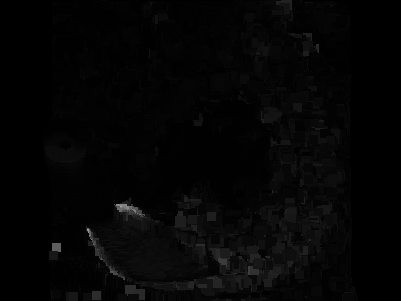

Supplement: S1 Data — The training datas are presented in the Supporting Information. (ZIP) [file pone.0275117.s002.zip › Rotating emitter/with filtering/originAcc-243.bmp]

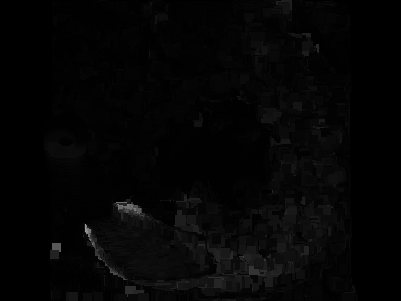

Supplement: S1 Data — The training datas are presented in the Supporting Information. (ZIP) [file pone.0275117.s002.zip › Rotating emitter/with filtering/originAcc-244.bmp]

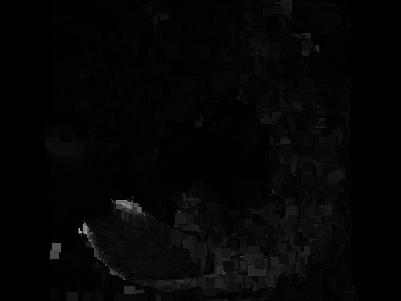

Supplement: S1 Data — The training datas are presented in the Supporting Information. (ZIP) [file pone.0275117.s002.zip › Rotating emitter/with filtering/originAcc-245.bmp]

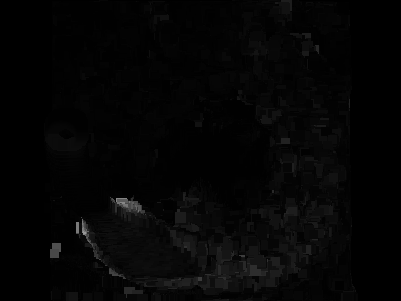

Supplement: S1 Data — The training datas are presented in the Supporting Information. (ZIP) [file pone.0275117.s002.zip › Rotating emitter/with filtering/originAcc-246.bmp]

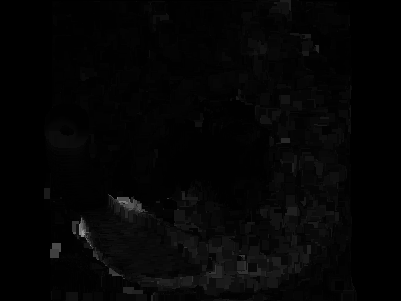

Supplement: S1 Data — The training datas are presented in the Supporting Information. (ZIP) [file pone.0275117.s002.zip › Rotating emitter/with filtering/originAcc-247.bmp]

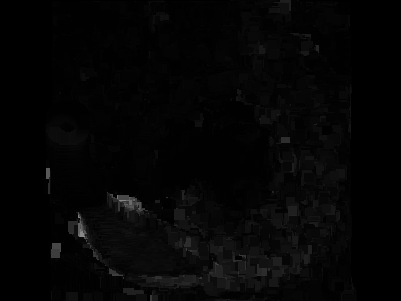

Supplement: S1 Data — The training datas are presented in the Supporting Information. (ZIP) [file pone.0275117.s002.zip › Rotating emitter/with filtering/originAcc-248.bmp]

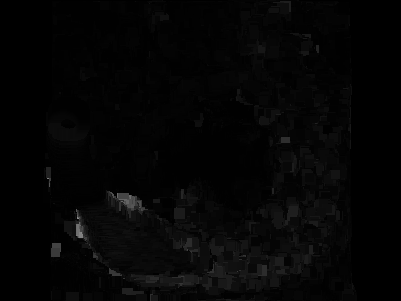

Supplement: S1 Data — The training datas are presented in the Supporting Information. (ZIP) [file pone.0275117.s002.zip › Rotating emitter/with filtering/originAcc-249.bmp]

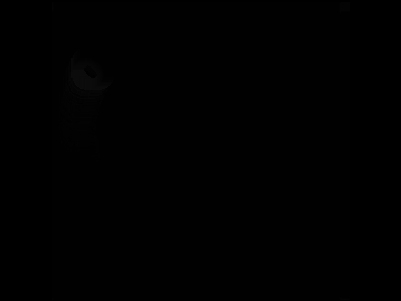

Supplement: S1 Data — The training datas are presented in the Supporting Information. (ZIP) [file pone.0275117.s002.zip › Rotating emitter/with filtering/originAcc-25.bmp]

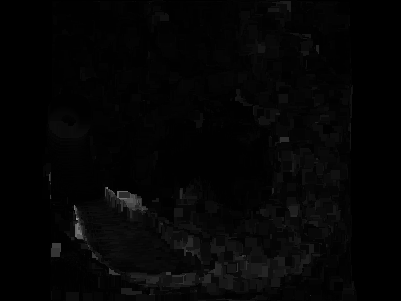

Supplement: S1 Data — The training datas are presented in the Supporting Information. (ZIP) [file pone.0275117.s002.zip › Rotating emitter/with filtering/originAcc-250.bmp]

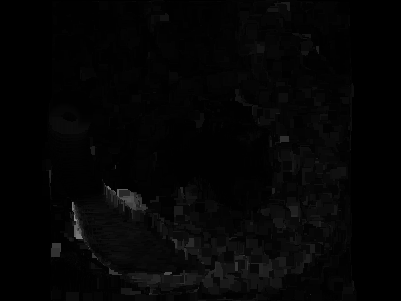

Supplement: S1 Data — The training datas are presented in the Supporting Information. (ZIP) [file pone.0275117.s002.zip › Rotating emitter/with filtering/originAcc-251.bmp]

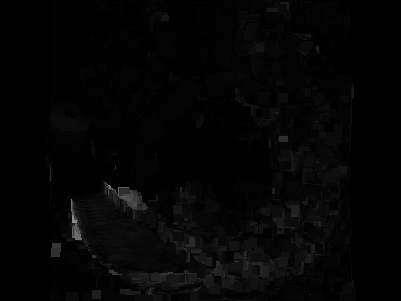

Supplement: S1 Data — The training datas are presented in the Supporting Information. (ZIP) [file pone.0275117.s002.zip › Rotating emitter/with filtering/originAcc-252.bmp]

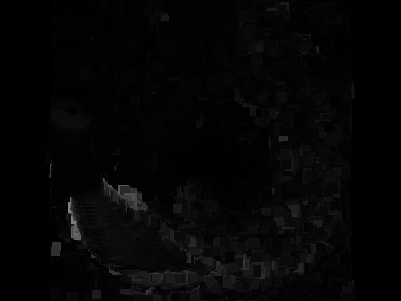

Supplement: S1 Data — The training datas are presented in the Supporting Information. (ZIP) [file pone.0275117.s002.zip › Rotating emitter/with filtering/originAcc-253.bmp]

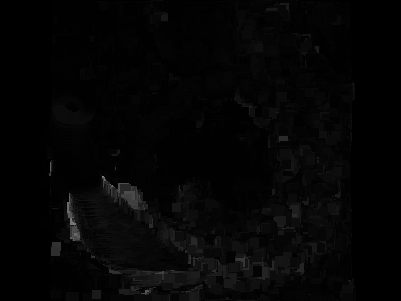

Supplement: S1 Data — The training datas are presented in the Supporting Information. (ZIP) [file pone.0275117.s002.zip › Rotating emitter/with filtering/originAcc-254.bmp]

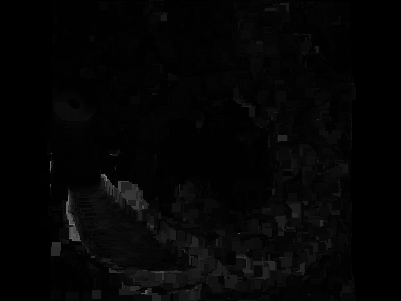

Supplement: S1 Data — The training datas are presented in the Supporting Information. (ZIP) [file pone.0275117.s002.zip › Rotating emitter/with filtering/originAcc-255.bmp]

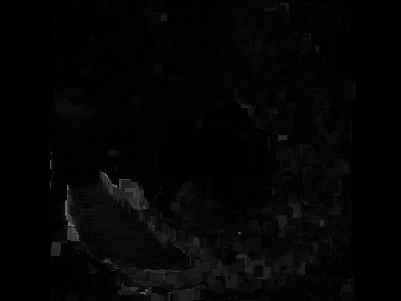

Supplement: S1 Data — The training datas are presented in the Supporting Information. (ZIP) [file pone.0275117.s002.zip › Rotating emitter/with filtering/originAcc-256.bmp]

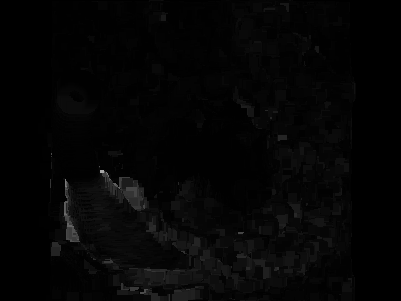

Supplement: S1 Data — The training datas are presented in the Supporting Information. (ZIP) [file pone.0275117.s002.zip › Rotating emitter/with filtering/originAcc-257.bmp]

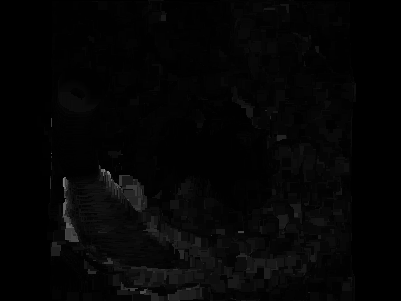

Supplement: S1 Data — The training datas are presented in the Supporting Information. (ZIP) [file pone.0275117.s002.zip › Rotating emitter/with filtering/originAcc-258.bmp]

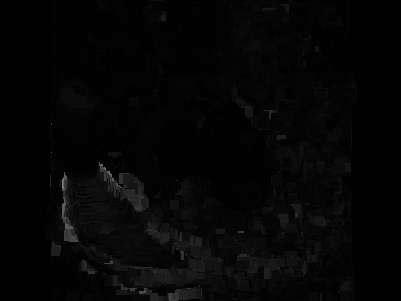

Supplement: S1 Data — The training datas are presented in the Supporting Information. (ZIP) [file pone.0275117.s002.zip › Rotating emitter/with filtering/originAcc-259.bmp]

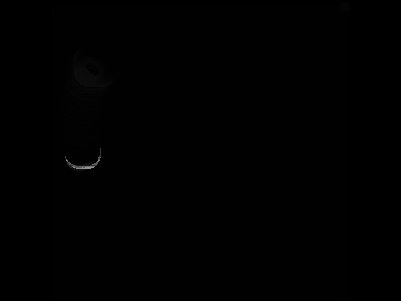

Supplement: S1 Data — The training datas are presented in the Supporting Information. (ZIP) [file pone.0275117.s002.zip › Rotating emitter/with filtering/originAcc-26.bmp]

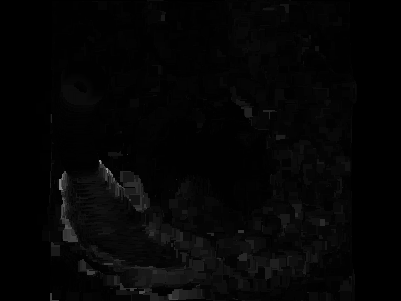

Supplement: S1 Data — The training datas are presented in the Supporting Information. (ZIP) [file pone.0275117.s002.zip › Rotating emitter/with filtering/originAcc-260.bmp]

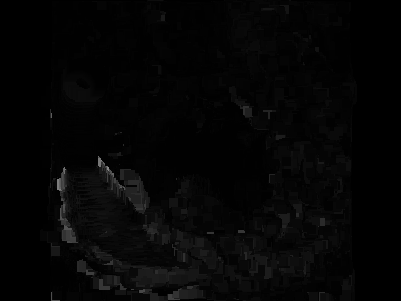

Supplement: S1 Data — The training datas are presented in the Supporting Information. (ZIP) [file pone.0275117.s002.zip › Rotating emitter/with filtering/originAcc-261.bmp]

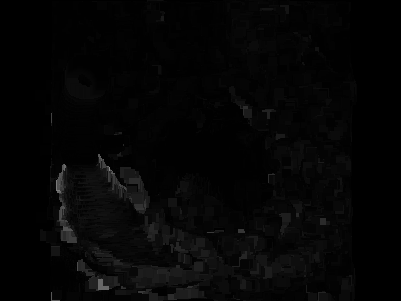

Supplement: S1 Data — The training datas are presented in the Supporting Information. (ZIP) [file pone.0275117.s002.zip › Rotating emitter/with filtering/originAcc-262.bmp]

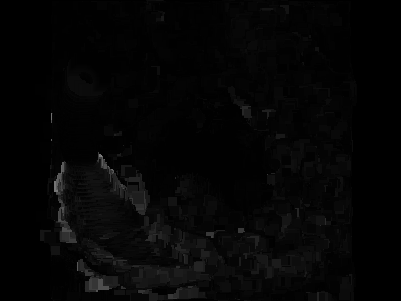

Supplement: S1 Data — The training datas are presented in the Supporting Information. (ZIP) [file pone.0275117.s002.zip › Rotating emitter/with filtering/originAcc-263.bmp]

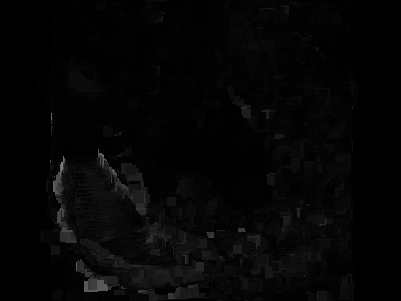

Supplement: S1 Data — The training datas are presented in the Supporting Information. (ZIP) [file pone.0275117.s002.zip › Rotating emitter/with filtering/originAcc-264.bmp]

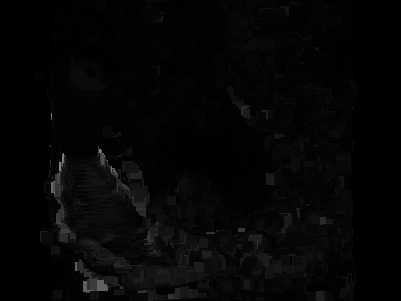

Supplement: S1 Data — The training datas are presented in the Supporting Information. (ZIP) [file pone.0275117.s002.zip › Rotating emitter/with filtering/originAcc-265.bmp]

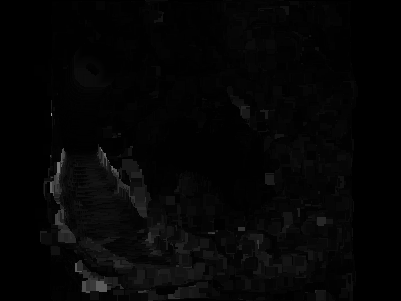

Supplement: S1 Data — The training datas are presented in the Supporting Information. (ZIP) [file pone.0275117.s002.zip › Rotating emitter/with filtering/originAcc-266.bmp]

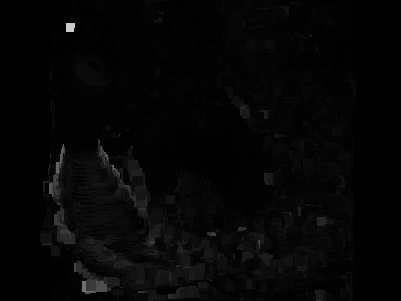

Supplement: S1 Data — The training datas are presented in the Supporting Information. (ZIP) [file pone.0275117.s002.zip › Rotating emitter/with filtering/originAcc-267.bmp]

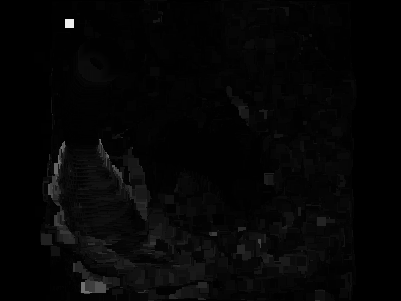

Supplement: S1 Data — The training datas are presented in the Supporting Information. (ZIP) [file pone.0275117.s002.zip › Rotating emitter/with filtering/originAcc-268.bmp]

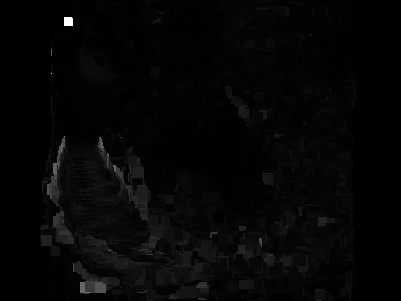

Supplement: S1 Data — The training datas are presented in the Supporting Information. (ZIP) [file pone.0275117.s002.zip › Rotating emitter/with filtering/originAcc-269.bmp]

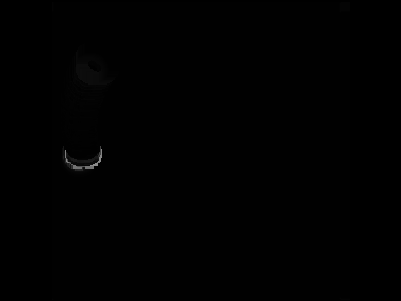

Supplement: S1 Data — The training datas are presented in the Supporting Information. (ZIP) [file pone.0275117.s002.zip › Rotating emitter/with filtering/originAcc-27.bmp]

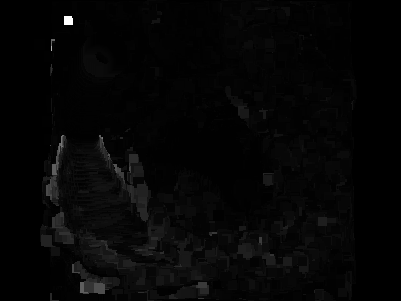

Supplement: S1 Data — The training datas are presented in the Supporting Information. (ZIP) [file pone.0275117.s002.zip › Rotating emitter/with filtering/originAcc-270.bmp]

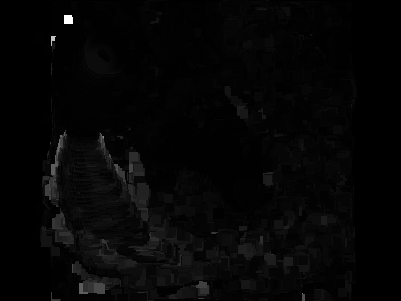

Supplement: S1 Data — The training datas are presented in the Supporting Information. (ZIP) [file pone.0275117.s002.zip › Rotating emitter/with filtering/originAcc-271.bmp]

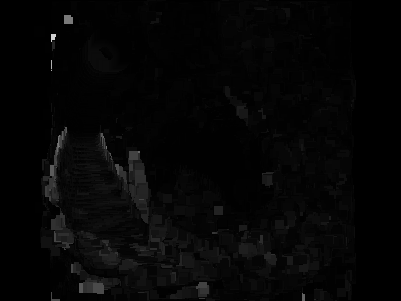

Supplement: S1 Data — The training datas are presented in the Supporting Information. (ZIP) [file pone.0275117.s002.zip › Rotating emitter/with filtering/originAcc-272.bmp]

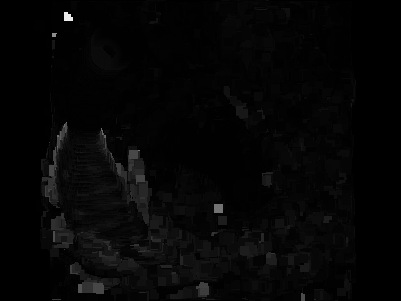

Supplement: S1 Data — The training datas are presented in the Supporting Information. (ZIP) [file pone.0275117.s002.zip › Rotating emitter/with filtering/originAcc-273.bmp]

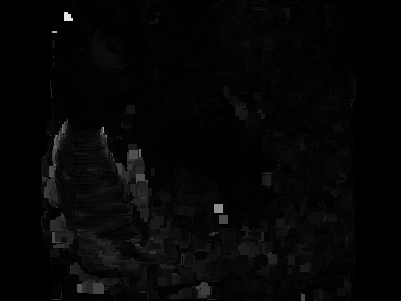

Supplement: S1 Data — The training datas are presented in the Supporting Information. (ZIP) [file pone.0275117.s002.zip › Rotating emitter/with filtering/originAcc-274.bmp]

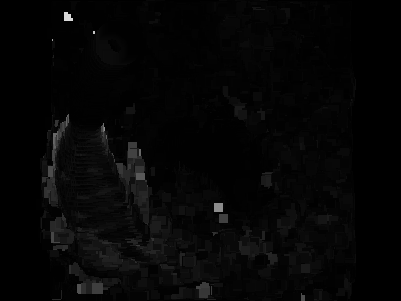

Supplement: S1 Data — The training datas are presented in the Supporting Information. (ZIP) [file pone.0275117.s002.zip › Rotating emitter/with filtering/originAcc-275.bmp]

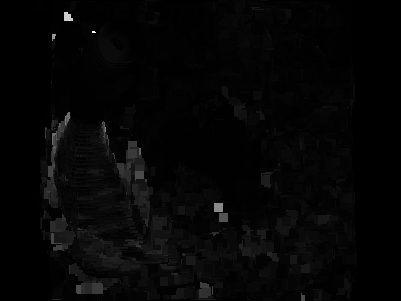

Supplement: S1 Data — The training datas are presented in the Supporting Information. (ZIP) [file pone.0275117.s002.zip › Rotating emitter/with filtering/originAcc-276.bmp]

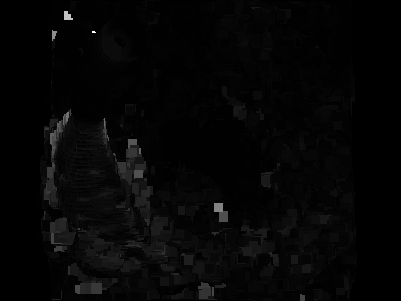

Supplement: S1 Data — The training datas are presented in the Supporting Information. (ZIP) [file pone.0275117.s002.zip › Rotating emitter/with filtering/originAcc-277.bmp]

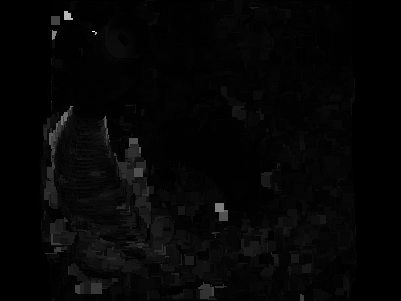

Supplement: S1 Data — The training datas are presented in the Supporting Information. (ZIP) [file pone.0275117.s002.zip › Rotating emitter/with filtering/originAcc-278.bmp]
