# Supplementary figures and images for: Efficient learning representation of noise-reduced foam effects with convolutional denoising networks (part 1 of 2)
Source: PLoS One. 2022 Oct 10;17(10):e0275117. doi: 10.1371/journal.pone.0275117 (PMC9551625; doi:10.1371/journal.pone.0275117)

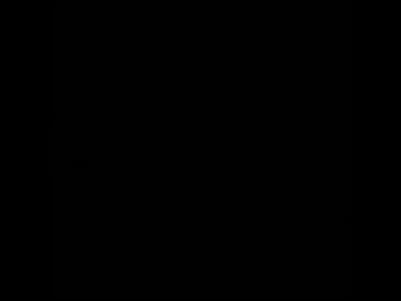

Supplement: S1 Data — The training datas are presented in the Supporting Information. (ZIP) [file pone.0275117.s002.zip › Rotating emitter/with filtering/originAcc-0.bmp]

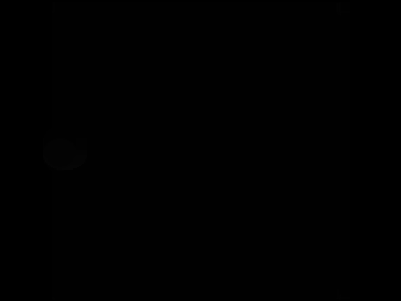

Supplement: S1 Data — The training datas are presented in the Supporting Information. (ZIP) [file pone.0275117.s002.zip › Rotating emitter/with filtering/originAcc-1.bmp]

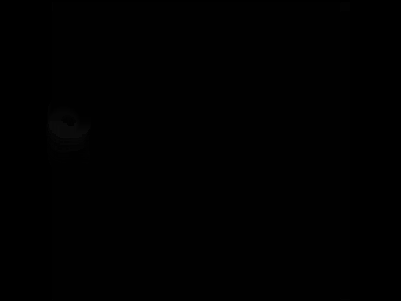

Supplement: S1 Data — The training datas are presented in the Supporting Information. (ZIP) [file pone.0275117.s002.zip › Rotating emitter/with filtering/originAcc-10.bmp]

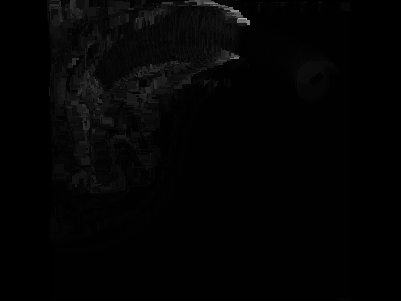

Supplement: S1 Data — The training datas are presented in the Supporting Information. (ZIP) [file pone.0275117.s002.zip › Rotating emitter/with filtering/originAcc-100.bmp]

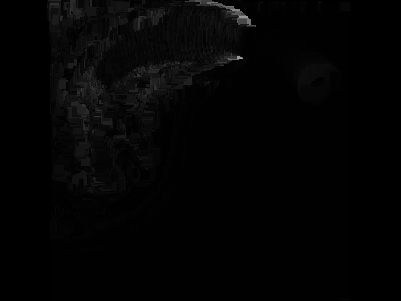

Supplement: S1 Data — The training datas are presented in the Supporting Information. (ZIP) [file pone.0275117.s002.zip › Rotating emitter/with filtering/originAcc-101.bmp]

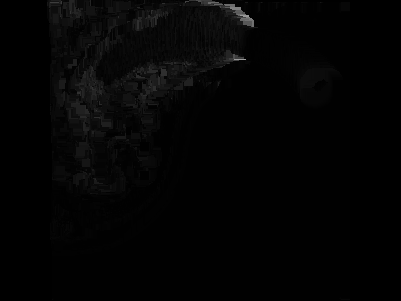

Supplement: S1 Data — The training datas are presented in the Supporting Information. (ZIP) [file pone.0275117.s002.zip › Rotating emitter/with filtering/originAcc-102.bmp]

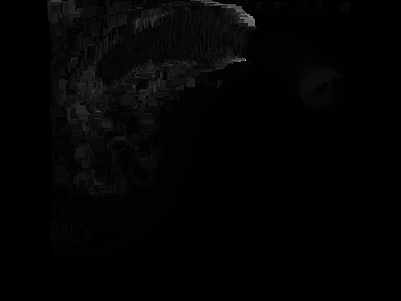

Supplement: S1 Data — The training datas are presented in the Supporting Information. (ZIP) [file pone.0275117.s002.zip › Rotating emitter/with filtering/originAcc-103.bmp]

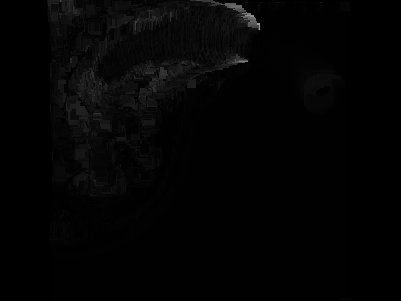

Supplement: S1 Data — The training datas are presented in the Supporting Information. (ZIP) [file pone.0275117.s002.zip › Rotating emitter/with filtering/originAcc-104.bmp]

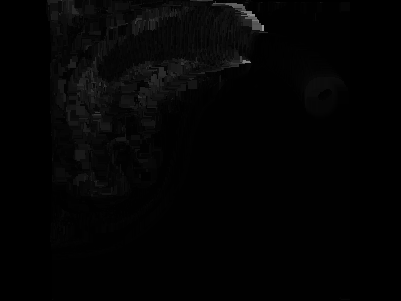

Supplement: S1 Data — The training datas are presented in the Supporting Information. (ZIP) [file pone.0275117.s002.zip › Rotating emitter/with filtering/originAcc-105.bmp]

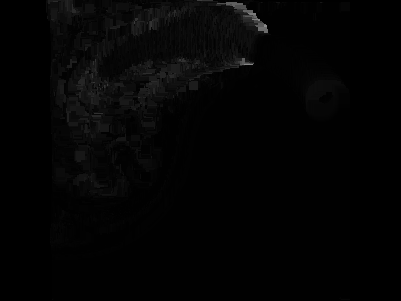

Supplement: S1 Data — The training datas are presented in the Supporting Information. (ZIP) [file pone.0275117.s002.zip › Rotating emitter/with filtering/originAcc-106.bmp]

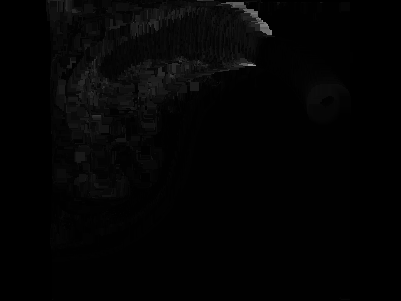

Supplement: S1 Data — The training datas are presented in the Supporting Information. (ZIP) [file pone.0275117.s002.zip › Rotating emitter/with filtering/originAcc-107.bmp]

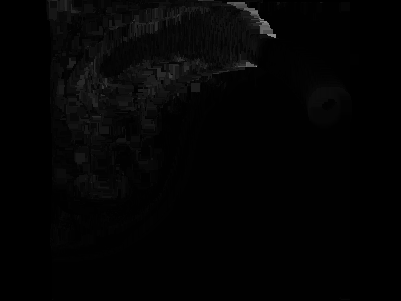

Supplement: S1 Data — The training datas are presented in the Supporting Information. (ZIP) [file pone.0275117.s002.zip › Rotating emitter/with filtering/originAcc-108.bmp]

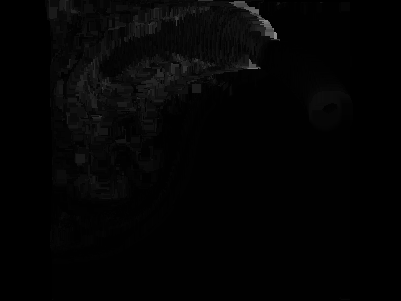

Supplement: S1 Data — The training datas are presented in the Supporting Information. (ZIP) [file pone.0275117.s002.zip › Rotating emitter/with filtering/originAcc-109.bmp]

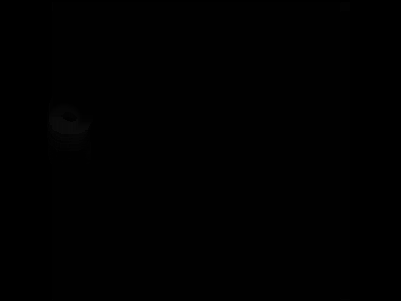

Supplement: S1 Data — The training datas are presented in the Supporting Information. (ZIP) [file pone.0275117.s002.zip › Rotating emitter/with filtering/originAcc-11.bmp]

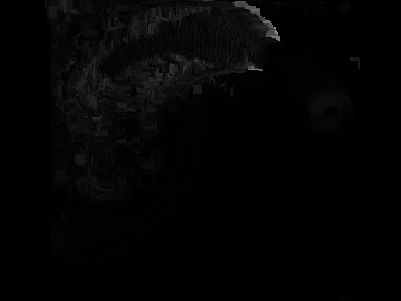

Supplement: S1 Data — The training datas are presented in the Supporting Information. (ZIP) [file pone.0275117.s002.zip › Rotating emitter/with filtering/originAcc-110.bmp]

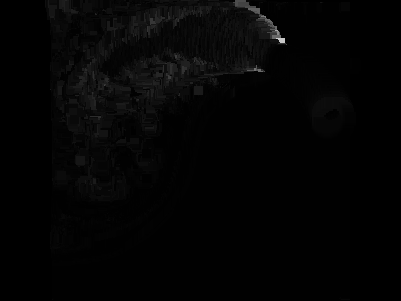

Supplement: S1 Data — The training datas are presented in the Supporting Information. (ZIP) [file pone.0275117.s002.zip › Rotating emitter/with filtering/originAcc-111.bmp]

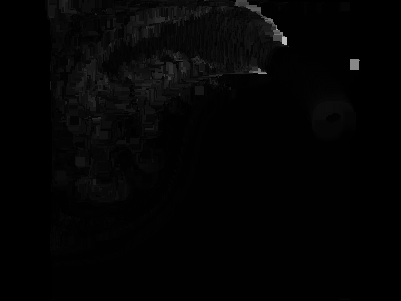

Supplement: S1 Data — The training datas are presented in the Supporting Information. (ZIP) [file pone.0275117.s002.zip › Rotating emitter/with filtering/originAcc-112.bmp]

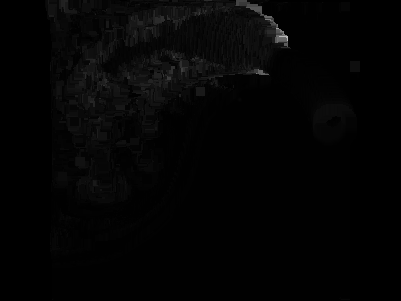

Supplement: S1 Data — The training datas are presented in the Supporting Information. (ZIP) [file pone.0275117.s002.zip › Rotating emitter/with filtering/originAcc-113.bmp]

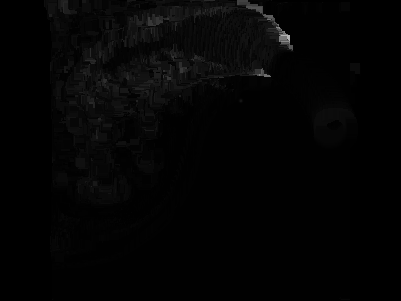

Supplement: S1 Data — The training datas are presented in the Supporting Information. (ZIP) [file pone.0275117.s002.zip › Rotating emitter/with filtering/originAcc-114.bmp]

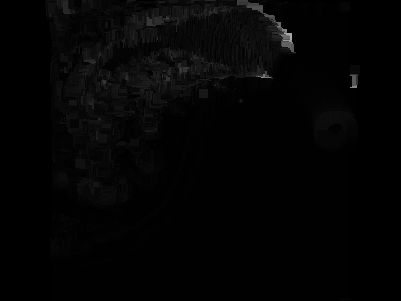

Supplement: S1 Data — The training datas are presented in the Supporting Information. (ZIP) [file pone.0275117.s002.zip › Rotating emitter/with filtering/originAcc-115.bmp]

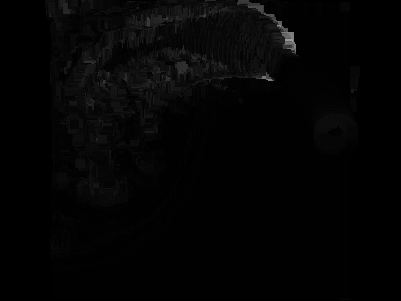

Supplement: S1 Data — The training datas are presented in the Supporting Information. (ZIP) [file pone.0275117.s002.zip › Rotating emitter/with filtering/originAcc-116.bmp]

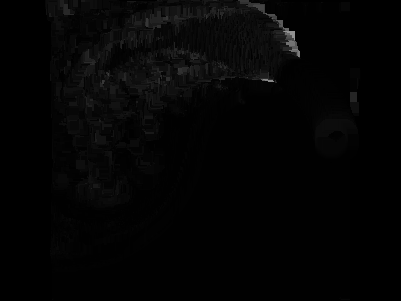

Supplement: S1 Data — The training datas are presented in the Supporting Information. (ZIP) [file pone.0275117.s002.zip › Rotating emitter/with filtering/originAcc-117.bmp]

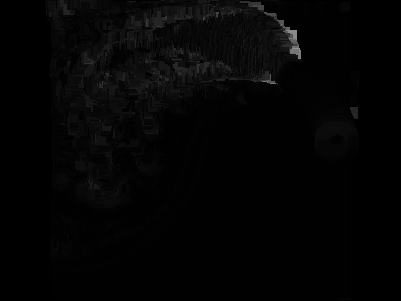

Supplement: S1 Data — The training datas are presented in the Supporting Information. (ZIP) [file pone.0275117.s002.zip › Rotating emitter/with filtering/originAcc-118.bmp]

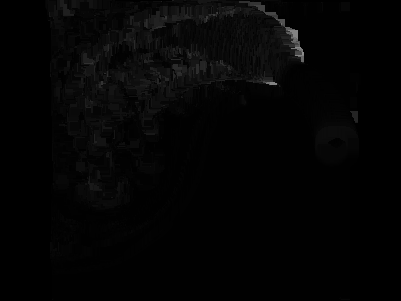

Supplement: S1 Data — The training datas are presented in the Supporting Information. (ZIP) [file pone.0275117.s002.zip › Rotating emitter/with filtering/originAcc-119.bmp]

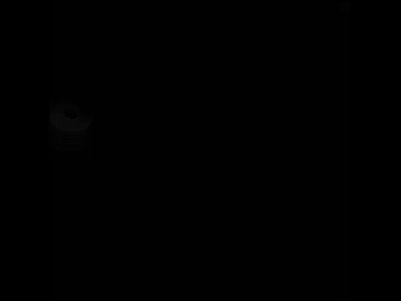

Supplement: S1 Data — The training datas are presented in the Supporting Information. (ZIP) [file pone.0275117.s002.zip › Rotating emitter/with filtering/originAcc-12.bmp]

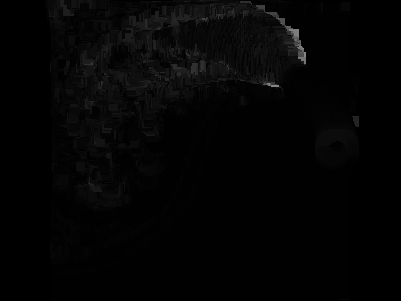

Supplement: S1 Data — The training datas are presented in the Supporting Information. (ZIP) [file pone.0275117.s002.zip › Rotating emitter/with filtering/originAcc-120.bmp]

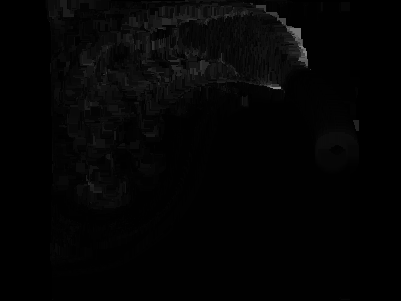

Supplement: S1 Data — The training datas are presented in the Supporting Information. (ZIP) [file pone.0275117.s002.zip › Rotating emitter/with filtering/originAcc-121.bmp]

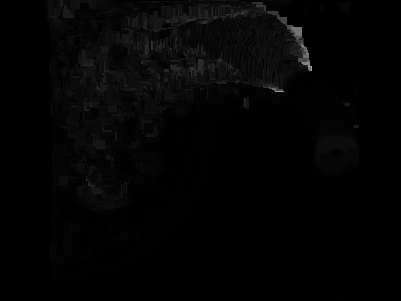

Supplement: S1 Data — The training datas are presented in the Supporting Information. (ZIP) [file pone.0275117.s002.zip › Rotating emitter/with filtering/originAcc-122.bmp]

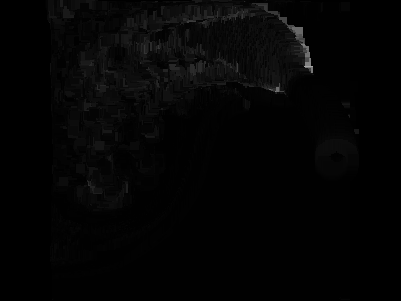

Supplement: S1 Data — The training datas are presented in the Supporting Information. (ZIP) [file pone.0275117.s002.zip › Rotating emitter/with filtering/originAcc-123.bmp]

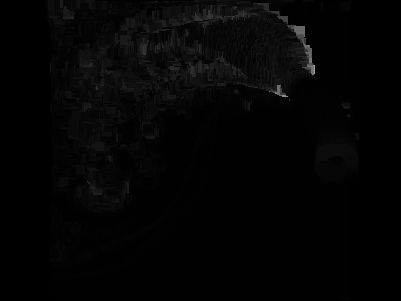

Supplement: S1 Data — The training datas are presented in the Supporting Information. (ZIP) [file pone.0275117.s002.zip › Rotating emitter/with filtering/originAcc-124.bmp]

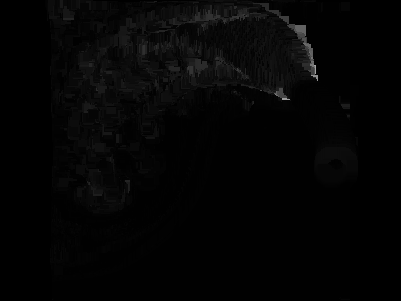

Supplement: S1 Data — The training datas are presented in the Supporting Information. (ZIP) [file pone.0275117.s002.zip › Rotating emitter/with filtering/originAcc-125.bmp]

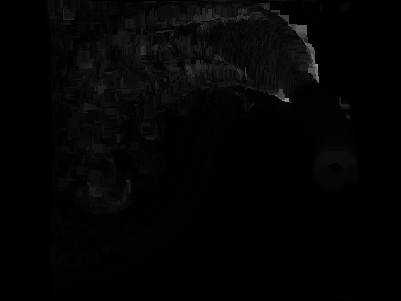

Supplement: S1 Data — The training datas are presented in the Supporting Information. (ZIP) [file pone.0275117.s002.zip › Rotating emitter/with filtering/originAcc-126.bmp]

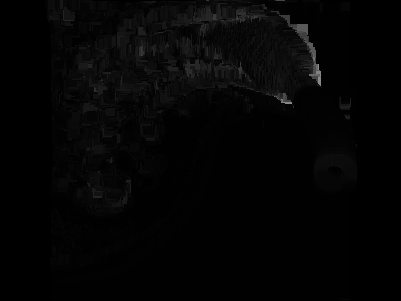

Supplement: S1 Data — The training datas are presented in the Supporting Information. (ZIP) [file pone.0275117.s002.zip › Rotating emitter/with filtering/originAcc-127.bmp]

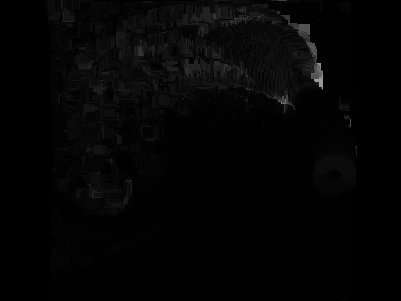

Supplement: S1 Data — The training datas are presented in the Supporting Information. (ZIP) [file pone.0275117.s002.zip › Rotating emitter/with filtering/originAcc-128.bmp]

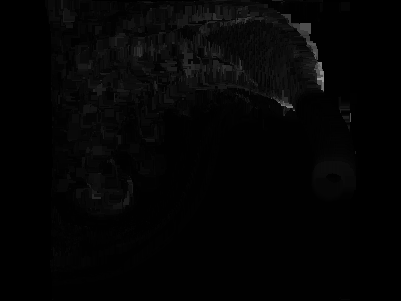

Supplement: S1 Data — The training datas are presented in the Supporting Information. (ZIP) [file pone.0275117.s002.zip › Rotating emitter/with filtering/originAcc-129.bmp]

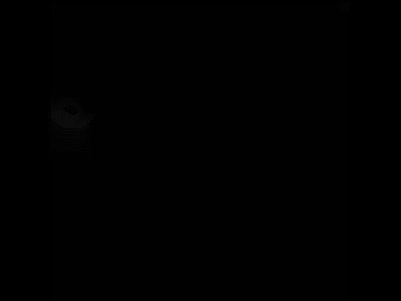

Supplement: S1 Data — The training datas are presented in the Supporting Information. (ZIP) [file pone.0275117.s002.zip › Rotating emitter/with filtering/originAcc-13.bmp]

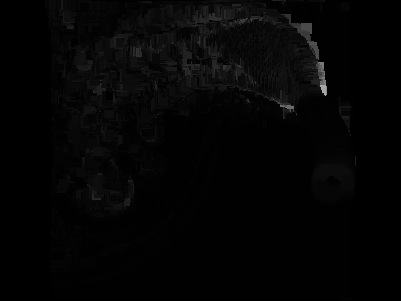

Supplement: S1 Data — The training datas are presented in the Supporting Information. (ZIP) [file pone.0275117.s002.zip › Rotating emitter/with filtering/originAcc-130.bmp]

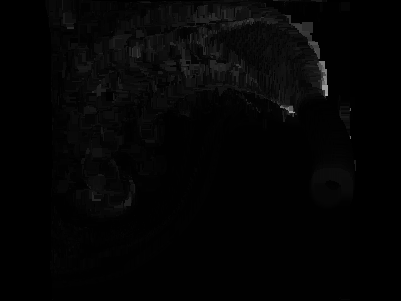

Supplement: S1 Data — The training datas are presented in the Supporting Information. (ZIP) [file pone.0275117.s002.zip › Rotating emitter/with filtering/originAcc-131.bmp]

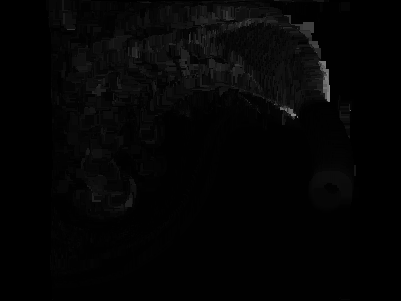

Supplement: S1 Data — The training datas are presented in the Supporting Information. (ZIP) [file pone.0275117.s002.zip › Rotating emitter/with filtering/originAcc-132.bmp]

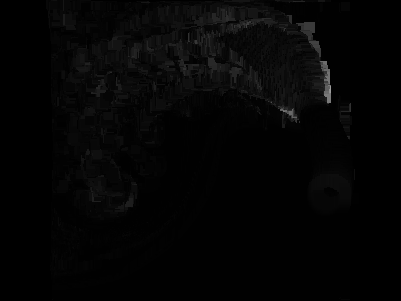

Supplement: S1 Data — The training datas are presented in the Supporting Information. (ZIP) [file pone.0275117.s002.zip › Rotating emitter/with filtering/originAcc-133.bmp]

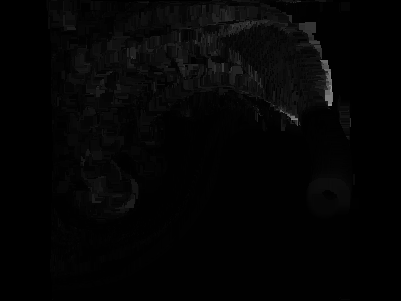

Supplement: S1 Data — The training datas are presented in the Supporting Information. (ZIP) [file pone.0275117.s002.zip › Rotating emitter/with filtering/originAcc-134.bmp]

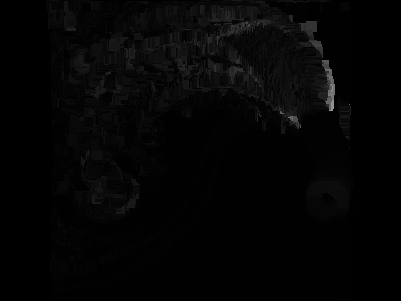

Supplement: S1 Data — The training datas are presented in the Supporting Information. (ZIP) [file pone.0275117.s002.zip › Rotating emitter/with filtering/originAcc-135.bmp]

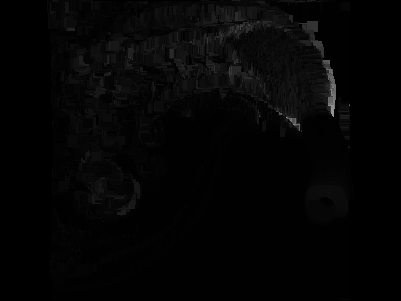

Supplement: S1 Data — The training datas are presented in the Supporting Information. (ZIP) [file pone.0275117.s002.zip › Rotating emitter/with filtering/originAcc-136.bmp]

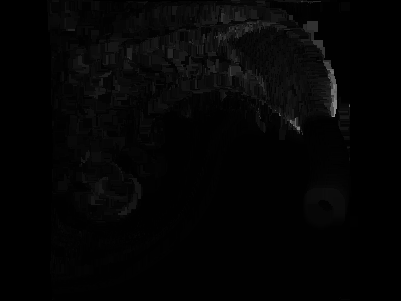

Supplement: S1 Data — The training datas are presented in the Supporting Information. (ZIP) [file pone.0275117.s002.zip › Rotating emitter/with filtering/originAcc-137.bmp]

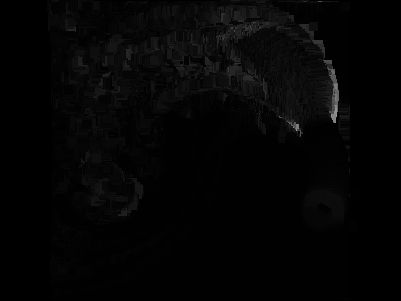

Supplement: S1 Data — The training datas are presented in the Supporting Information. (ZIP) [file pone.0275117.s002.zip › Rotating emitter/with filtering/originAcc-138.bmp]

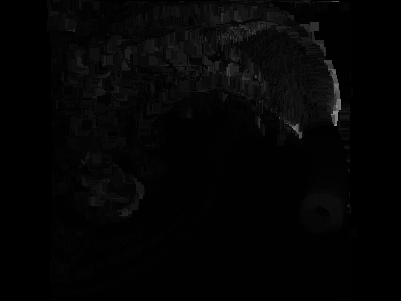

Supplement: S1 Data — The training datas are presented in the Supporting Information. (ZIP) [file pone.0275117.s002.zip › Rotating emitter/with filtering/originAcc-139.bmp]

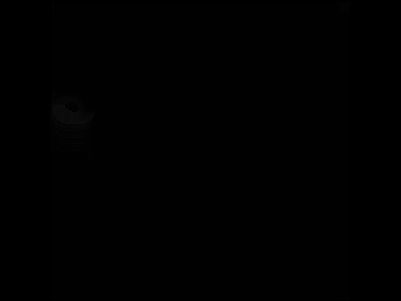

Supplement: S1 Data — The training datas are presented in the Supporting Information. (ZIP) [file pone.0275117.s002.zip › Rotating emitter/with filtering/originAcc-14.bmp]

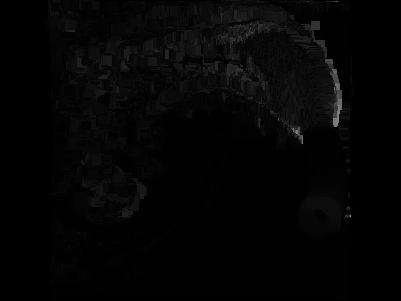

Supplement: S1 Data — The training datas are presented in the Supporting Information. (ZIP) [file pone.0275117.s002.zip › Rotating emitter/with filtering/originAcc-140.bmp]

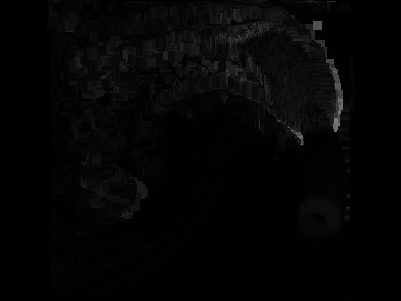

Supplement: S1 Data — The training datas are presented in the Supporting Information. (ZIP) [file pone.0275117.s002.zip › Rotating emitter/with filtering/originAcc-141.bmp]

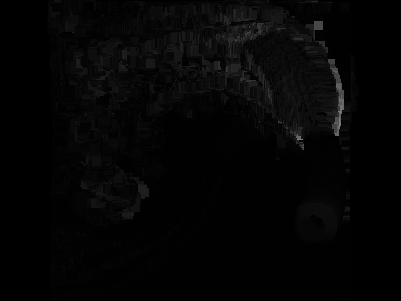

Supplement: S1 Data — The training datas are presented in the Supporting Information. (ZIP) [file pone.0275117.s002.zip › Rotating emitter/with filtering/originAcc-142.bmp]

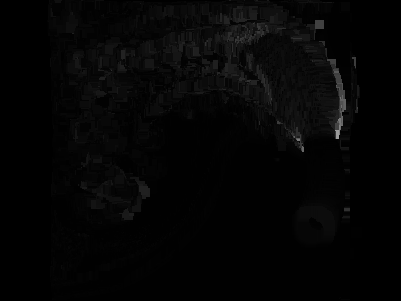

Supplement: S1 Data — The training datas are presented in the Supporting Information. (ZIP) [file pone.0275117.s002.zip › Rotating emitter/with filtering/originAcc-143.bmp]

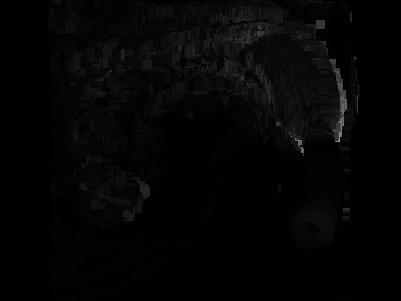

Supplement: S1 Data — The training datas are presented in the Supporting Information. (ZIP) [file pone.0275117.s002.zip › Rotating emitter/with filtering/originAcc-144.bmp]

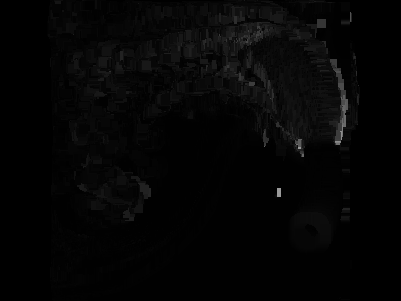

Supplement: S1 Data — The training datas are presented in the Supporting Information. (ZIP) [file pone.0275117.s002.zip › Rotating emitter/with filtering/originAcc-145.bmp]

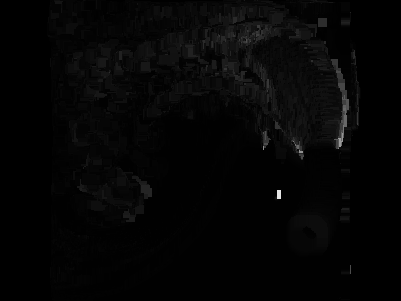

Supplement: S1 Data — The training datas are presented in the Supporting Information. (ZIP) [file pone.0275117.s002.zip › Rotating emitter/with filtering/originAcc-146.bmp]

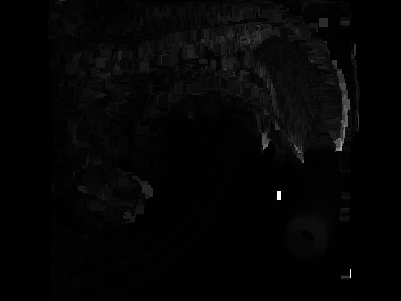

Supplement: S1 Data — The training datas are presented in the Supporting Information. (ZIP) [file pone.0275117.s002.zip › Rotating emitter/with filtering/originAcc-147.bmp]

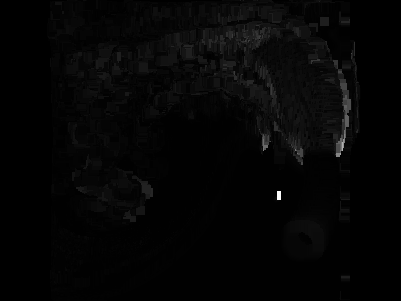

Supplement: S1 Data — The training datas are presented in the Supporting Information. (ZIP) [file pone.0275117.s002.zip › Rotating emitter/with filtering/originAcc-148.bmp]

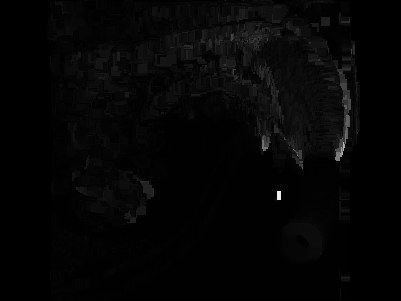

Supplement: S1 Data — The training datas are presented in the Supporting Information. (ZIP) [file pone.0275117.s002.zip › Rotating emitter/with filtering/originAcc-149.bmp]

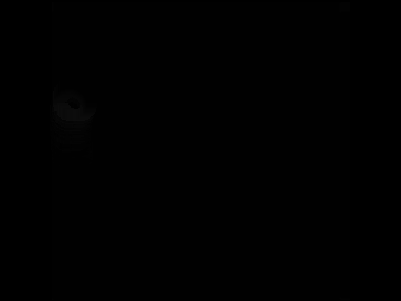

Supplement: S1 Data — The training datas are presented in the Supporting Information. (ZIP) [file pone.0275117.s002.zip › Rotating emitter/with filtering/originAcc-15.bmp]

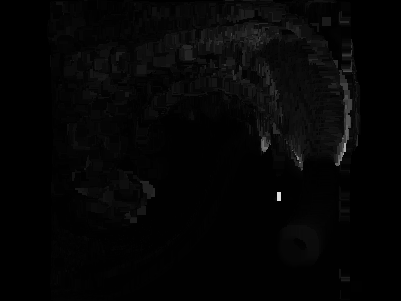

Supplement: S1 Data — The training datas are presented in the Supporting Information. (ZIP) [file pone.0275117.s002.zip › Rotating emitter/with filtering/originAcc-150.bmp]

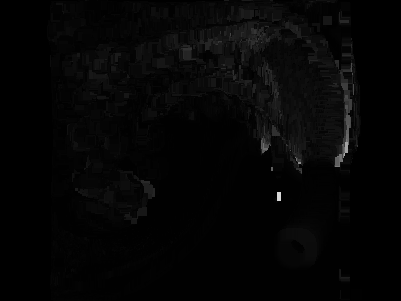

Supplement: S1 Data — The training datas are presented in the Supporting Information. (ZIP) [file pone.0275117.s002.zip › Rotating emitter/with filtering/originAcc-151.bmp]

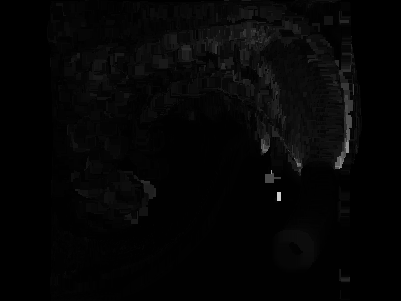

Supplement: S1 Data — The training datas are presented in the Supporting Information. (ZIP) [file pone.0275117.s002.zip › Rotating emitter/with filtering/originAcc-152.bmp]

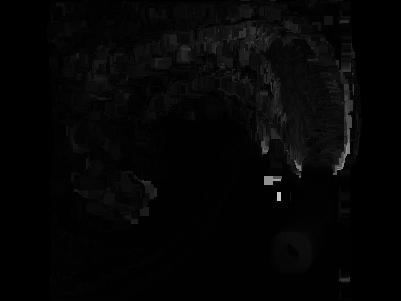

Supplement: S1 Data — The training datas are presented in the Supporting Information. (ZIP) [file pone.0275117.s002.zip › Rotating emitter/with filtering/originAcc-153.bmp]

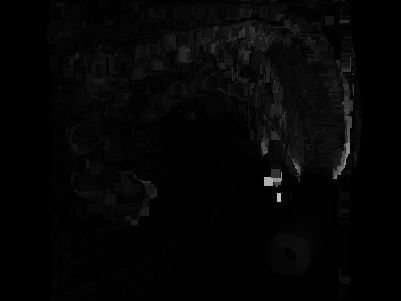

Supplement: S1 Data — The training datas are presented in the Supporting Information. (ZIP) [file pone.0275117.s002.zip › Rotating emitter/with filtering/originAcc-154.bmp]

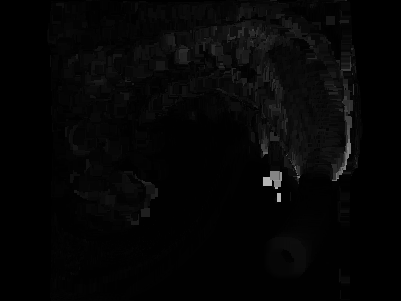

Supplement: S1 Data — The training datas are presented in the Supporting Information. (ZIP) [file pone.0275117.s002.zip › Rotating emitter/with filtering/originAcc-155.bmp]

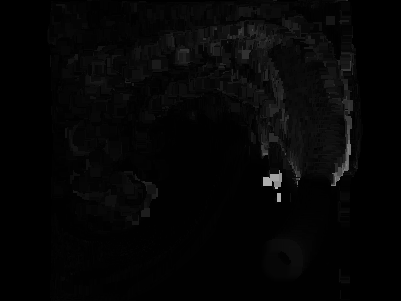

Supplement: S1 Data — The training datas are presented in the Supporting Information. (ZIP) [file pone.0275117.s002.zip › Rotating emitter/with filtering/originAcc-156.bmp]

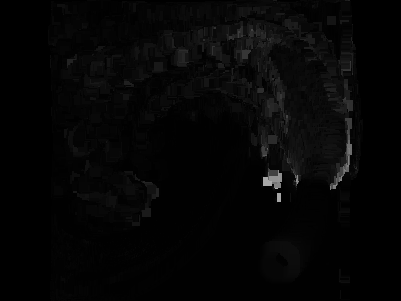

Supplement: S1 Data — The training datas are presented in the Supporting Information. (ZIP) [file pone.0275117.s002.zip › Rotating emitter/with filtering/originAcc-157.bmp]

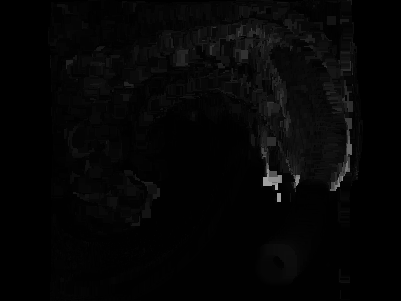

Supplement: S1 Data — The training datas are presented in the Supporting Information. (ZIP) [file pone.0275117.s002.zip › Rotating emitter/with filtering/originAcc-158.bmp]

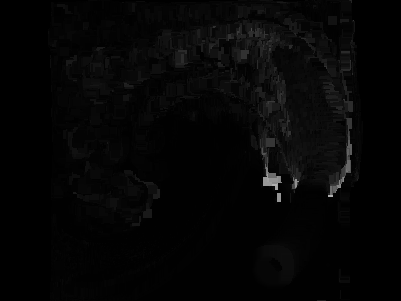

Supplement: S1 Data — The training datas are presented in the Supporting Information. (ZIP) [file pone.0275117.s002.zip › Rotating emitter/with filtering/originAcc-159.bmp]

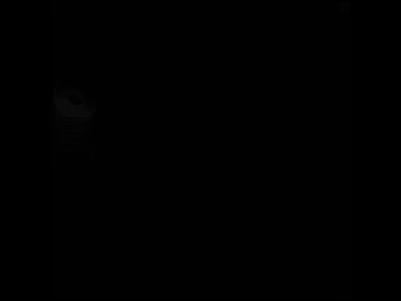

Supplement: S1 Data — The training datas are presented in the Supporting Information. (ZIP) [file pone.0275117.s002.zip › Rotating emitter/with filtering/originAcc-16.bmp]

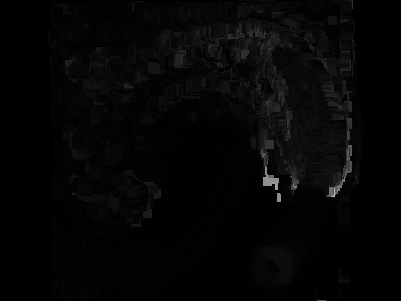

Supplement: S1 Data — The training datas are presented in the Supporting Information. (ZIP) [file pone.0275117.s002.zip › Rotating emitter/with filtering/originAcc-160.bmp]

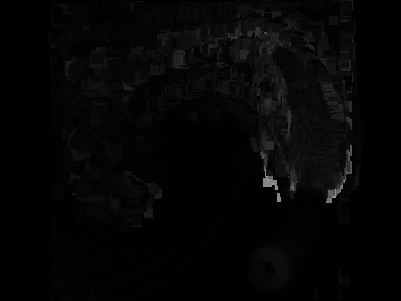

Supplement: S1 Data — The training datas are presented in the Supporting Information. (ZIP) [file pone.0275117.s002.zip › Rotating emitter/with filtering/originAcc-161.bmp]

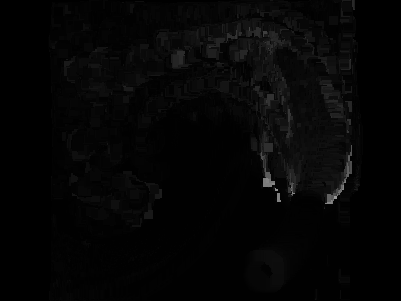

Supplement: S1 Data — The training datas are presented in the Supporting Information. (ZIP) [file pone.0275117.s002.zip › Rotating emitter/with filtering/originAcc-162.bmp]

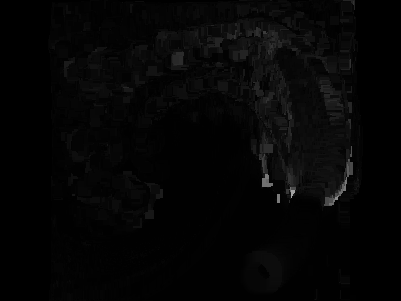

Supplement: S1 Data — The training datas are presented in the Supporting Information. (ZIP) [file pone.0275117.s002.zip › Rotating emitter/with filtering/originAcc-163.bmp]

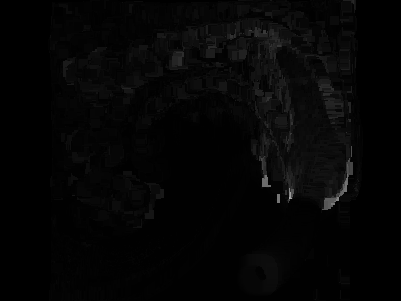

Supplement: S1 Data — The training datas are presented in the Supporting Information. (ZIP) [file pone.0275117.s002.zip › Rotating emitter/with filtering/originAcc-164.bmp]

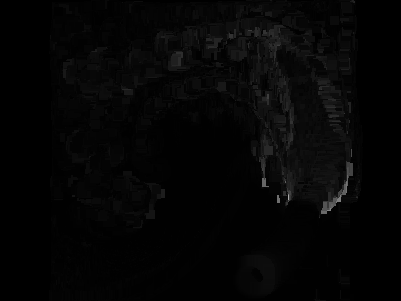

Supplement: S1 Data — The training datas are presented in the Supporting Information. (ZIP) [file pone.0275117.s002.zip › Rotating emitter/with filtering/originAcc-165.bmp]

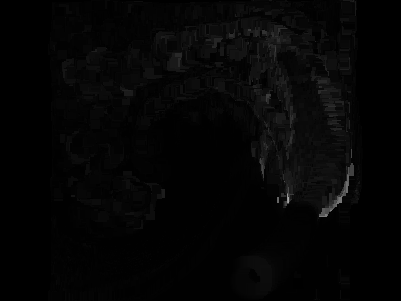

Supplement: S1 Data — The training datas are presented in the Supporting Information. (ZIP) [file pone.0275117.s002.zip › Rotating emitter/with filtering/originAcc-166.bmp]

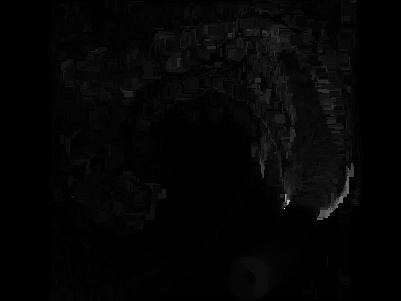

Supplement: S1 Data — The training datas are presented in the Supporting Information. (ZIP) [file pone.0275117.s002.zip › Rotating emitter/with filtering/originAcc-167.bmp]

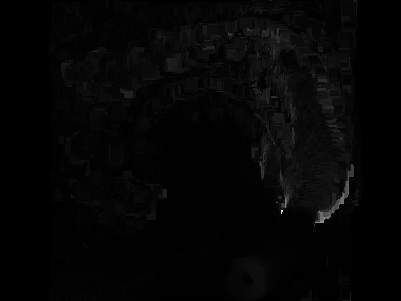

Supplement: S1 Data — The training datas are presented in the Supporting Information. (ZIP) [file pone.0275117.s002.zip › Rotating emitter/with filtering/originAcc-168.bmp]

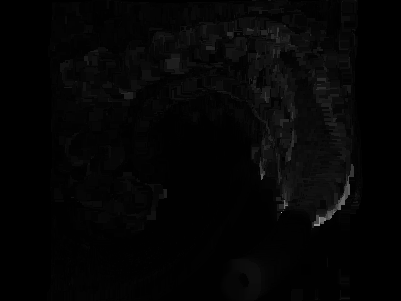

Supplement: S1 Data — The training datas are presented in the Supporting Information. (ZIP) [file pone.0275117.s002.zip › Rotating emitter/with filtering/originAcc-169.bmp]

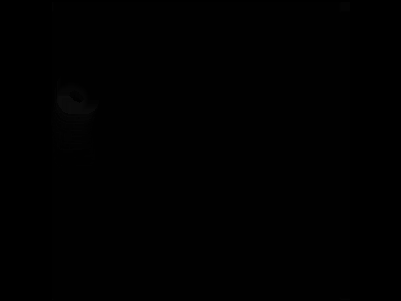

Supplement: S1 Data — The training datas are presented in the Supporting Information. (ZIP) [file pone.0275117.s002.zip › Rotating emitter/with filtering/originAcc-17.bmp]

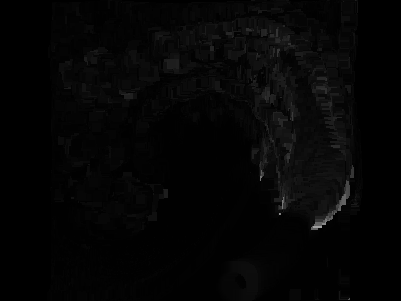

Supplement: S1 Data — The training datas are presented in the Supporting Information. (ZIP) [file pone.0275117.s002.zip › Rotating emitter/with filtering/originAcc-170.bmp]

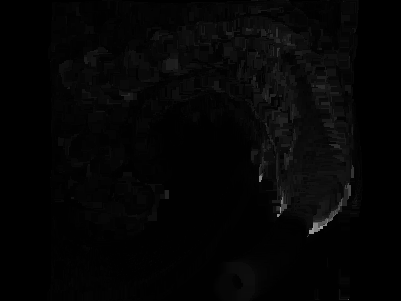

Supplement: S1 Data — The training datas are presented in the Supporting Information. (ZIP) [file pone.0275117.s002.zip › Rotating emitter/with filtering/originAcc-171.bmp]

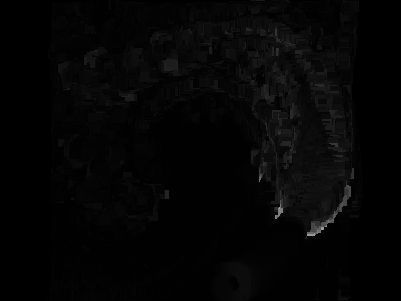

Supplement: S1 Data — The training datas are presented in the Supporting Information. (ZIP) [file pone.0275117.s002.zip › Rotating emitter/with filtering/originAcc-172.bmp]

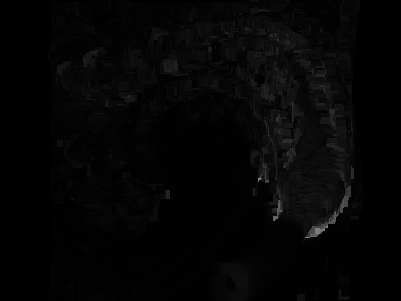

Supplement: S1 Data — The training datas are presented in the Supporting Information. (ZIP) [file pone.0275117.s002.zip › Rotating emitter/with filtering/originAcc-173.bmp]

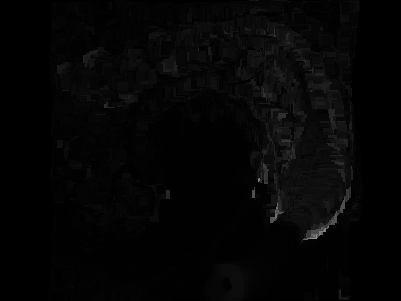

Supplement: S1 Data — The training datas are presented in the Supporting Information. (ZIP) [file pone.0275117.s002.zip › Rotating emitter/with filtering/originAcc-174.bmp]

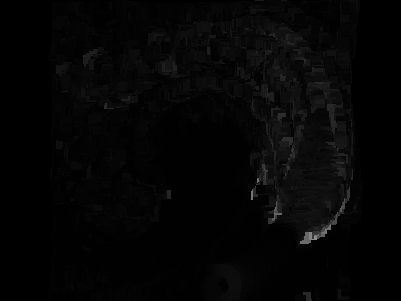

Supplement: S1 Data — The training datas are presented in the Supporting Information. (ZIP) [file pone.0275117.s002.zip › Rotating emitter/with filtering/originAcc-175.bmp]

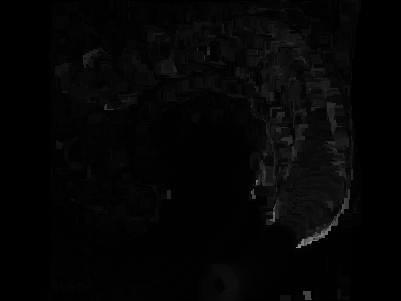

Supplement: S1 Data — The training datas are presented in the Supporting Information. (ZIP) [file pone.0275117.s002.zip › Rotating emitter/with filtering/originAcc-176.bmp]

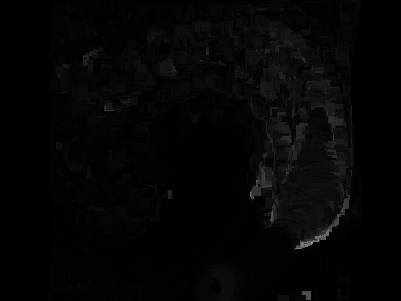

Supplement: S1 Data — The training datas are presented in the Supporting Information. (ZIP) [file pone.0275117.s002.zip › Rotating emitter/with filtering/originAcc-177.bmp]

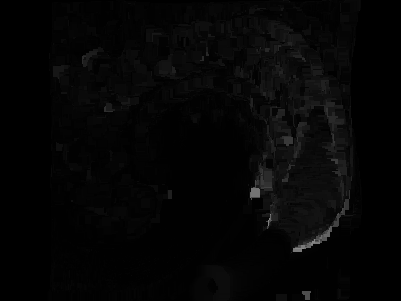

Supplement: S1 Data — The training datas are presented in the Supporting Information. (ZIP) [file pone.0275117.s002.zip › Rotating emitter/with filtering/originAcc-178.bmp]

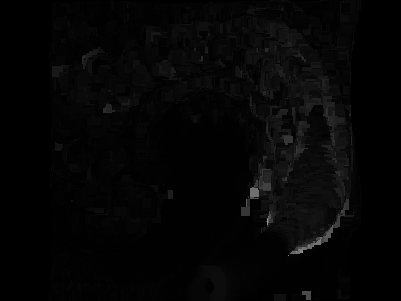

Supplement: S1 Data — The training datas are presented in the Supporting Information. (ZIP) [file pone.0275117.s002.zip › Rotating emitter/with filtering/originAcc-179.bmp]

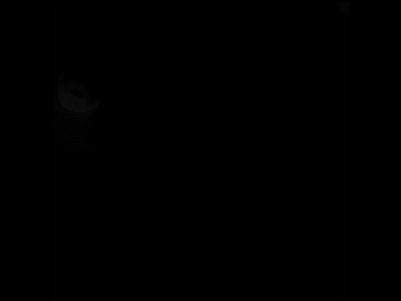

Supplement: S1 Data — The training datas are presented in the Supporting Information. (ZIP) [file pone.0275117.s002.zip › Rotating emitter/with filtering/originAcc-18.bmp]

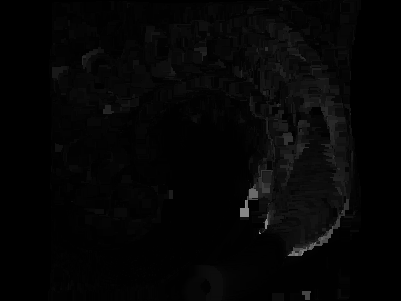

Supplement: S1 Data — The training datas are presented in the Supporting Information. (ZIP) [file pone.0275117.s002.zip › Rotating emitter/with filtering/originAcc-180.bmp]

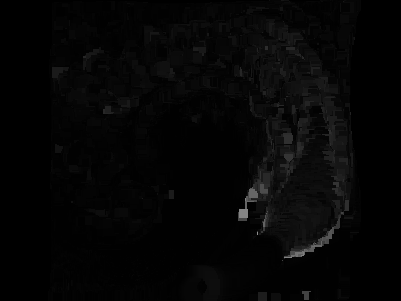

Supplement: S1 Data — The training datas are presented in the Supporting Information. (ZIP) [file pone.0275117.s002.zip › Rotating emitter/with filtering/originAcc-181.bmp]

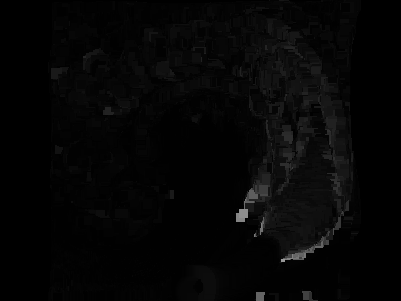

Supplement: S1 Data — The training datas are presented in the Supporting Information. (ZIP) [file pone.0275117.s002.zip › Rotating emitter/with filtering/originAcc-182.bmp]

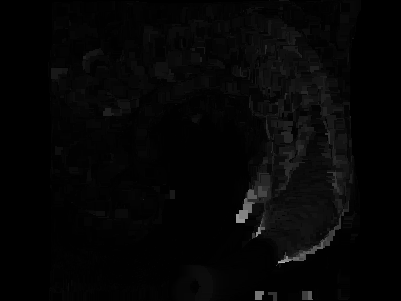

Supplement: S1 Data — The training datas are presented in the Supporting Information. (ZIP) [file pone.0275117.s002.zip › Rotating emitter/with filtering/originAcc-183.bmp]

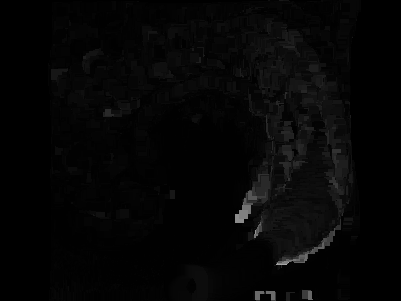

Supplement: S1 Data — The training datas are presented in the Supporting Information. (ZIP) [file pone.0275117.s002.zip › Rotating emitter/with filtering/originAcc-184.bmp]

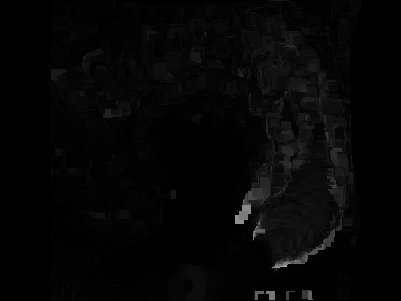

Supplement: S1 Data — The training datas are presented in the Supporting Information. (ZIP) [file pone.0275117.s002.zip › Rotating emitter/with filtering/originAcc-185.bmp]

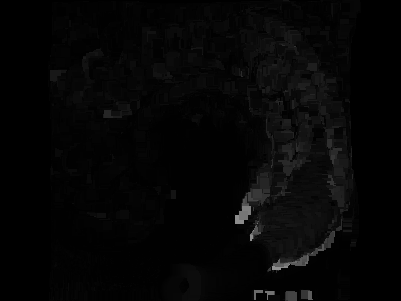

Supplement: S1 Data — The training datas are presented in the Supporting Information. (ZIP) [file pone.0275117.s002.zip › Rotating emitter/with filtering/originAcc-186.bmp]

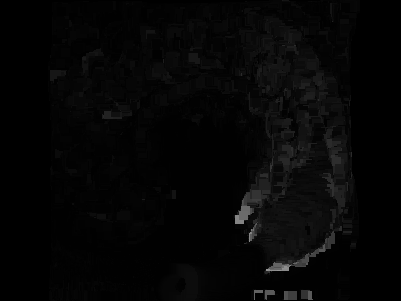

Supplement: S1 Data — The training datas are presented in the Supporting Information. (ZIP) [file pone.0275117.s002.zip › Rotating emitter/with filtering/originAcc-187.bmp]

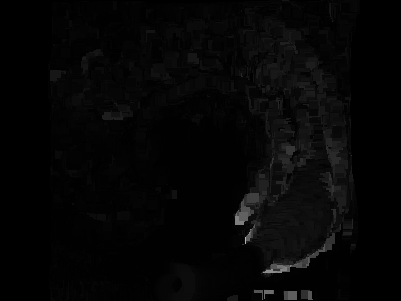

Supplement: S1 Data — The training datas are presented in the Supporting Information. (ZIP) [file pone.0275117.s002.zip › Rotating emitter/with filtering/originAcc-188.bmp]
